# Heterogeneity in proportional cardiovascular co-listing on cancer death certificates in the United States: a national multiple cause-of-death analysis, 1999–2020

**DOI:** 10.1186/s40959-026-00525-8

**Published:** 2026-06-12

**Authors:** Faizan Ahmed, Tehmasp Rehman Mirza, Yusra Junaid, Hassaan Abid, Haris Bin Tahir, Areehah Zafar Masood, Zaima Afzaal, Gabriel Benjamen, Brijesh Patel

**Affiliations:** 1 Department of Internal Medicine, Hackensack Meridian Health, Shore University Medical Center, Neptune, NJ USA; 2 Shalamar Medical & Dental College, Shalamar Institute of Health Sciences, Lahore, Punjab Pakistan; 3 Dow Medical College, Dow University of Health and Sciences, Karachi, Sindh Pakistan; 4 Department of Internal Medicine, IU Health Ball Memorial Hospital, Muncie, IN USA; 5 Lahore General Hospital, Lahore, Pakistan; 6 Department of Medicine, Ziauddin Medical College, Karachi, Sindh Pakistan; 7 Ameer-ud-din Medical College, Lahore, Pakistan; 8 School of Medicine, St. George’s University, St. George’s, Grenada; 9 Department of Cardiology, Indiana University School of Medicine, Indianapolis, IN USA

## Abstract

**Background:**

Cardiovascular disease (CVD) and cancer share common risk factors, and cancer survivors experience elevated long-term cardiotoxicity. Traditional risk-based metrics require person-time denominators that are unavailable in aggregate public health databases. This study introduces a proportional co-listing framework to detect population-level cardiovascular surveillance signals on death certificates across cancer types and demographic strata.

**Methods:**

We conducted a national cross-sectional surveillance analysis using CDC WONDER Multiple Cause-of-Death data from 1999 to 2020 for decedents aged ≥ 15 years. Utilizing an “any-mention” framework, we calculated the Proportional Mortality Ratio (PMR), defined as the ratio of CVD co-listing on cancer-related death certificates to CVD listing on non-cancer certificates. Temporal trends were evaluated using joinpoint regression to estimate the average annual percent change (AAPC). Analyses were stratified by age, sex, race/ethnicity, geography, and index cancer subtypes (lung, colon, and leukaemia).

**Results:**

Of 56,014,102 death certificates analyzed, 13,917,133 (24.8%) listed a malignant neoplasm, of which 3,997,238 (28.7%) co-listed CVD. Across the lifespan, the overall PMR followed a reverse J-shaped curve. Proportional co-listing was highest in the youngest cohort (ages 15–24: PMR 1.828, 95% CI: 1.778–1.879), declined to a nadir at ages 55–64 (PMR 0.336, 95% CI: 0.335–0.337), and rose modestly in older adulthood. From 1999 to 2020, PMRs increased significantly across all adult age groups, peaking at ages 55–64 (AAPC 1.69%, 95% CI: 1.52–1.83, *p* < 0.001). Young-adult PMR elevations were consistently higher in male, metropolitan, and Hispanic/Latino decedents. Among subtypes, leukaemia demonstrated the highest relative CVD co-listing in young adults (PMR 2.174), whereas colon cancer showed no statistically significant elevation (PMR 1.173, 95% CI: 0.951–1.447).

**Conclusions:**

Cardiovascular co-listing on US cancer death certificates is highly patterned by age, demographics, and cancer subtype. While elevated PMRs in young adults are consistent with patterns previously reported in cardio-oncology literature, sub-unity PMRs in middle-aged and older adults may reflect under-documentation of cardiovascular comorbidities in oncology settings alongside denominator effects arising from frequent CVD listing on non-cancer certificates. Improving comorbidity documentation on death certificates may enhance the utility of national administrative data for cardio-oncology surveillance and research.

**Supplementary Information:**

The online version contains supplementary material available at https://doi.org/10.1186/s40959-026-00525-8.

## Introduction

Cardiovascular disease (CVD) and cancer are the two leading causes of death in the United States, with similar risk factors, biological mechanisms, and a substantial demographic overlap [1]. Improving cancer survival has made late cardiovascular toxicity one of the central problems in cardio-oncology. A meta-analysis found cancer survivors die from CVD at approximately 1.55 times the rate of the general population (pooled SMR 1.55, 95% CI 1.40–1.72), though this ranges widely, from an SMR of 1.14 in testicular cancer survivors to 2.82 in brain tumour survivors [2]. Across 7.5 million cancer patients in a SEER-based study, the fatal heart disease SMR was 2.24 (95% CI 2.23–2.25), with risk increasing with time after diagnosis for almost all cancer types [3]. The excess is largest in younger patients: cardiac mortality SMRs among five-year cancer survivors diagnosed at age 15–19 years reach 4.2 (95% CI 3.4–5.2) and fall progressively to 1.2 (95% CI 1.1–1.3) for those diagnosed at 35–39 years [4, 5]. To examine whether this cardiovascular signal is detectable at a population level across cancer types, age groups, and demographic strata, we turned to the Centers for Disease Control (CDC) Wide-ranging ONline Data for Epidemiologic Research (WONDER) access tool to query Multiple Cause-of-Death (MCD) death certificates [6].

CDC WONDER provides aggregate cross-sectional death counts without person-time denominators, individual follow-up, treatment records, or information on surviving populations, precluding estimation of standardised mortality ratios, incidence rate ratios, or competing-risk models. Thus, a proportional mortality framework is more appropriate than traditional risk-based epidemiologic measures for evaluating cardiovascular co-listing within death-certificate records. Specifically, we apply a proportional co-listing ratio comparing the proportion of cancer-related death certificates listing CVD with the corresponding proportion among non-cancer certificates. It is conceptually related to the Proportional Mortality Ratio (PMR) used in occupational and disease-surveillance epidemiology for several decades [7–9]. However, unlike the classical occupational PMR, which compares observed-to-expected deaths against an external reference population’s cause-specific rates, this measure uses the non-cancer decedent population within the same dataset as its internal reference, making it appropriate for aggregate death-certificate data where person-time denominators are unavailable. The full construction and justification of this measure are described in the Methods. For brevity, this measure is referred to throughout as the PMR. Accordingly, the resulting measure should be interpreted as a measure of proportional representation within death-certificate records rather than an estimate of cardiovascular mortality risk, incidence, or hazard among cancer patients.

To our knowledge, this is the first study to apply this proportional co-listing framework to the full US Multiple Cause-of-Death record across cancer types, demographic strata, and the 1999–2020 period. The findings describe a surveillance signal rather than a risk estimate and should be interpreted as hypothesis-generating observations intended to inform future prospective cardio-oncology research.

## Methods

### Study design and data source

We performed a national proportional mortality surveillance analysis using CDC WONDER Multiple Cause-of-Death Public Use data, which contains de-identified, ICD-10-coded death certificates from all 50 US states and the District of Columbia [10]. Records from 1999 to 2020 for individuals aged 15 years or older were included. The data are publicly available and de-identified; institutional review board approval was not required. Reporting follows the STROBE guidelines [11].

#### Rationale for multiple-cause analysis

CDC WONDER’s multiple-cause files record every condition on the death certificate, not just the underlying cause. This matters here because when cancer is selected as the underlying cause, CVD listed as a contributing or antecedent condition would be invisible in a single-cause analysis. Restriction to underlying-cause data alone underestimates disease-associated mortality; prior analyses of US national mortality records found that multiple-cause approaches capture substantially more disease involvement at death than single-cause analyses [12, 13]. Multiple-cause analysis is particularly appropriate for multimorbidity surveillance because death certificates frequently contain clinically relevant contributing conditions that are not selected as the underlying cause of death [14]. We therefore use an any-mention framework throughout [15].

### Case definitions

Cardiovascular disease was defined by ICD-10 codes I00–I99; malignant neoplasms by C00–C97; lung cancer by C33–C34; leukaemia by C91–C95; and colon cancer by C18.

### Variable construction

Four quantities were extracted from CDC WONDER for each stratum:

- *A* = deaths listing both cancer (or the relevant cancer subtype) and CVD anywhere on the certificate;
- *B* = all deaths listing cancer (or relevant subtype) anywhere on the certificate;
- *C* = all deaths in the total population listing CVD anywhere on the certificate; and
- *D* = all-cause deaths in the total population.

The non-cancer reference quantities were (C − A) CVD deaths without cancer, and (D − B) all non-cancer deaths. A 2 × 2 contingency table with a worked numerical example is provided in Supplementary Material.

### Primary measure: proportional mortality ratio

The primary measure was the Proportional Mortality Ratio (PMR), defined as:

$$\begin{aligned} \mathrm{PMR} = \left(\mathrm{A} / \mathrm{B}\right) \div [\left(\mathrm{C}-\mathrm{A}\right) / \left(\mathrm{D}-\mathrm{B}\right)] \end{aligned}$$

A/B is the CVD co-listing proportion in cancer death certificates; (C − A)/(D − B) is the corresponding proportion in non-cancer certificates. PMR is a measure of proportional representation within death certificates and should not be interpreted as a measure of incidence, mortality risk, or hazard. A PMR above 1 indicates that CVD is proportionally more frequently co-listed on cancer-related certificates than on non-cancer certificates; a PMR below 1 indicates the reverse.

### Confidence intervals

Ninety-five percent confidence intervals used the log-scale delta method. The standard error of ln (PMR) was:

SE = $$\:\sqrt{\frac{1}{A}+\frac{1}{C-A}+\frac{1}{B}+\frac{1}{D-B}}$$ 

and the 95% CI was $$\begin{aligned} \mathrm{exp}[1\mathrm{n} \left(\mathrm{PMR}\right) \pm 1.96 \times \mathrm{SE}] \end{aligned}$$. PMR calculations and confidence intervals were performed in Microsoft Excel (Microsoft Corporation, Redmond, WA).

### Stratification

PMRs were computed for overall malignant neoplasms and for lung cancer, leukaemia, and colon cancer separately. Stratification was by age band (15–24, 25–34, 35–44, 45–54, 55–64, 65–74, 75–84, ≥ 85 years), sex, race/ethnicity (Non-Hispanic (NH) White; NH Black or African American; Hispanic or Latino; NH American Indian or Alaska Native; NH Asian or Pacific Islander), US Census region (Northeast, Midwest, South, West), urbanisation level (large central metropolitan to non-core), and place of death. Cells with fewer than 10 deaths were suppressed per CDC WONDER policy and excluded from stratum-specific analyses.

### Temporal-trend analysis

Annual PMR values from 1999 to 2020, with corresponding standard errors, were analysed using the Joinpoint Regression Program (version 5.3.0.0, National Cancer Institute) [16]. Log-linear models estimated annual percent change (APC) within each segment and average annual percent change (AAPC) across the full period, each with 95% CIs. Model selection used the Weighted Bayesian Information Criterion (WBIC), with a maximum of four joinpoints. Confidence intervals for APC and AAPC estimates were derived using the empirical quantile method based on 5,001 resamples. A two-sided P-value below 0.05 was considered statistically significant.

## Results

### Overall pattern across age groups

**Fig. 1:**
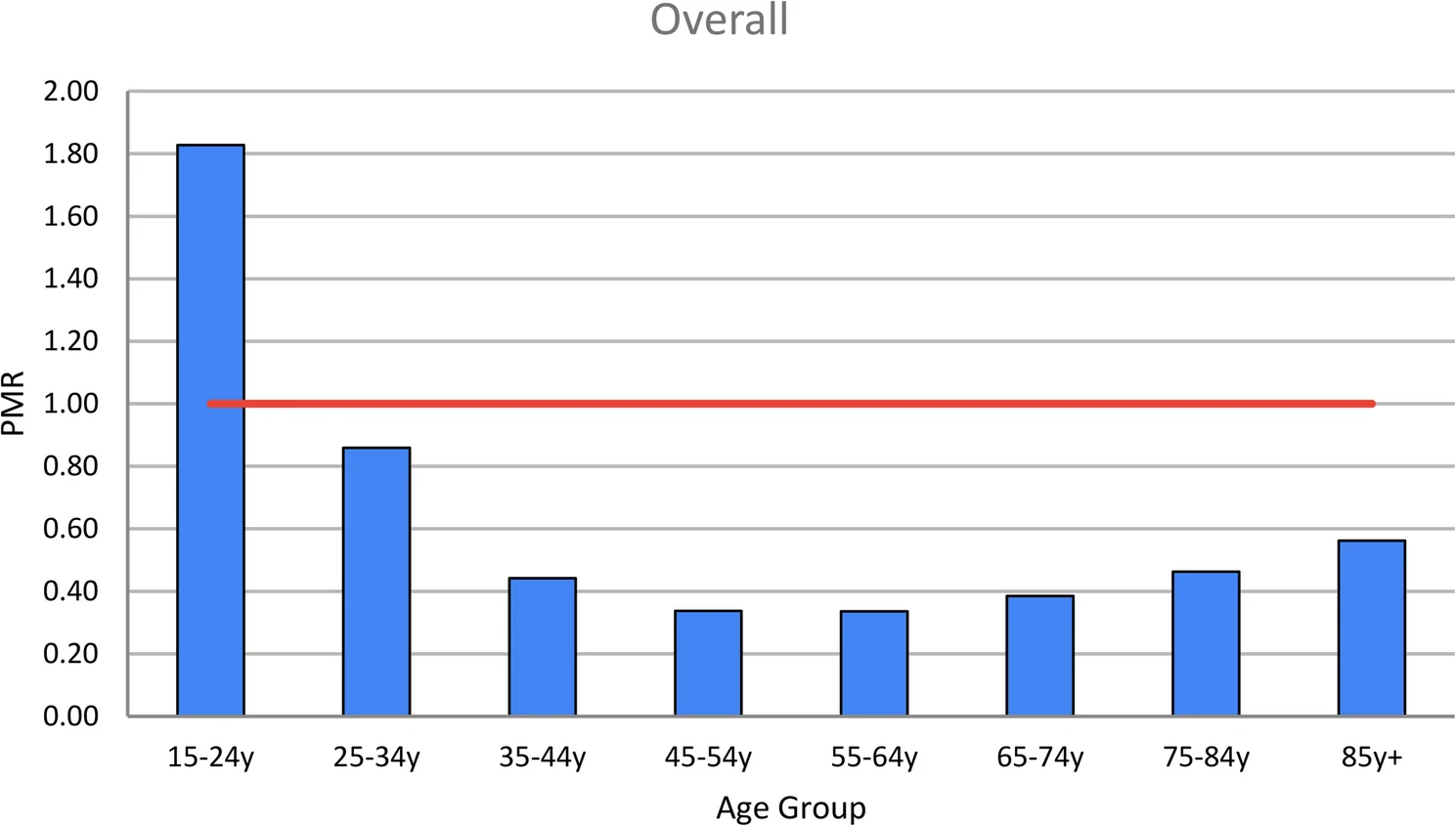
Proportional Mortality Ratio for cardiovascular co-listing on malignant neoplasm death certificates, by age group in the United States, 1999–2020

Between 1999 and 2020, 56,014,102 death certificates were analysed for individuals aged 15 years or older, of which 13,917,133 (24.8%) listed a malignant neoplasm and 3,997,238 (28.7% of cancer deaths) listed both a malignant neoplasm and cardiovascular disease anywhere on the certificate. CVD co-listing on cancer-related death certificates relative to non-cancer death certificates followed a reverse J-shaped pattern across the lifespan (Fig. 1; Supplementary Table 1): PMR was highest in the youngest decedents (15–24 years: 1.828, 95% CI: 1.778–1.879), declined progressively through middle age to a nadir at 55–64 years (0.336, 95% CI: 0.335–0.337), and rose modestly through older adulthood (65–74 years: 0.385; 75–84 years: 0.463; ≥85 years: 0.562). This pattern was accompanied by a substantially steeper rise in CVD co-listing among non-cancer decedents (approximately seven-fold between the youngest and oldest age groups) than among cancer decedents (roughly doubled) (Supplementary Table 3).

Temporal analyses demonstrated that PMR increased significantly across all adult age groups over the study period, with the sole exception of the 15–24-year group (AAPC 0.43%, 95% CI: −0.19 to 1.07, *p* = 0.15) (Supplementary Fig. 1; Supplementary Table 2). The steepest annual increase was in the 55–64-year group (AAPC 1.69%, 95% CI: 1.52–1.83, *p* < 0.001), driven by a simultaneous rise in CVD co-listing on cancer death certificates (A/B AAPC 0.96%, 95% CI: 0.85–1.07) and a decline in CVD co-listing on non-cancer death certificates (C/D AAPC − 0.67%, 95% CI: −0.72 to − 0.63) over the study period (Supplementary Table 2). Significant upward trends were present at all other adult age groups, ranging from an AAPC of 1.47% at 45–54 years (95% CI: 1.34–1.61) to 0.66% at ≥ 85 years (95% CI: 0.52–0.76).

### Sex stratification

**Fig. 2:**
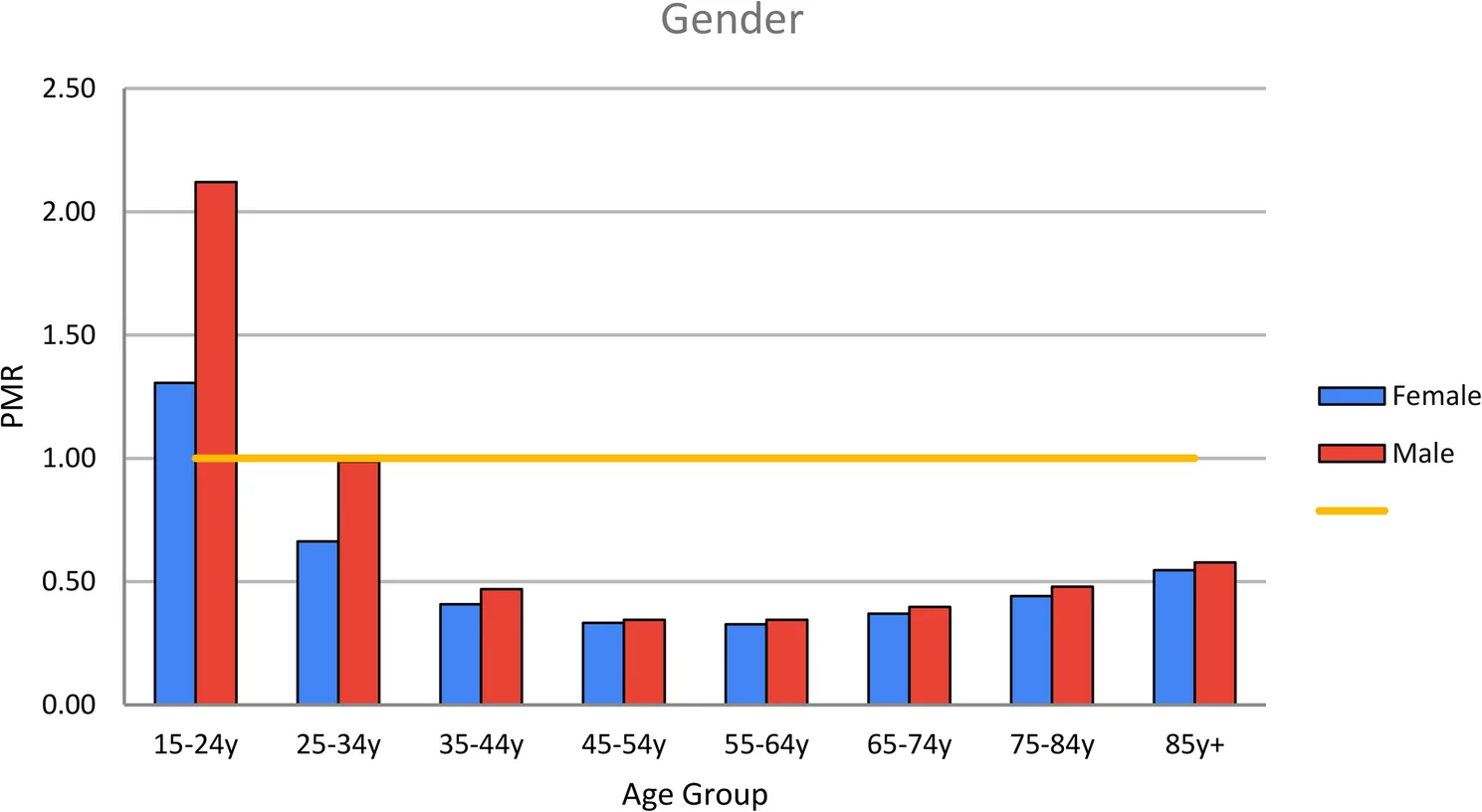
Proportional Mortality Ratio for cardiovascular co-listing on malignant neoplasm death certificates, stratified by sex and age group in the United States, 1999–2020

Male decedents had consistently higher PMRs than female decedents across all age groups (Fig. 2; Supplementary Table 4). The sex difference was widest in young adults: at 15–24 years, PMR was 2.120 (95% CI: 2.047–2.196) in males and 1.306 (95% CI: 1.249–1.364) in females. The gap narrowed with age and was smallest in the oldest group (≥ 85 years: males 0.578, 95% CI: 0.576–0.580; females 0.546, 95% CI: 0.544–0.548). Temporal analyses showed significant positive AAPCs in both sexes across all adult age groups; 55–64 years had the highest rise, with AAPC of 1.61% (95% CI: 1.48–1.75) in males and 1.69% (95% CI: 1.50–1.85) in females (Supplementary Table 2).

### Regional and urbanisation stratification

**Fig. 3:**
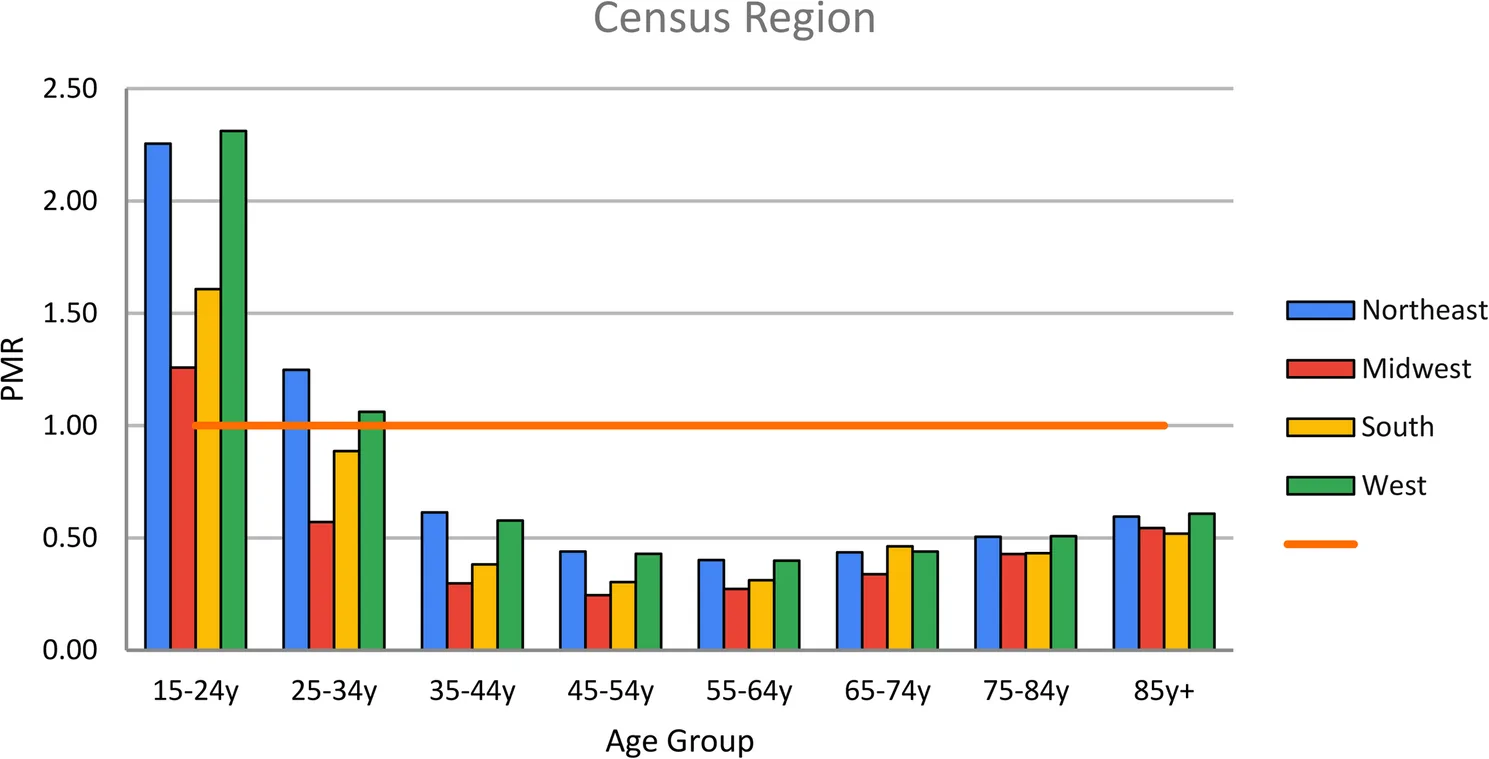
Proportional Mortality Ratio for cardiovascular co-listing on malignant neoplasm death certificates, stratified by Census region and age group in the United States, 1999–2020

At 15–24 years, the West and Northeast census regions had the highest proportional CVD representation (West: 2.311, 95% CI: 2.196–2.433; Northeast: 2.255, 95% CI: 2.122–2.396), followed by the South (1.607, 95% CI: 1.533–1.684) and Midwest (1.258, 95% CI: 1.171–1.351) (Fig. 3; Supplementary Table 5). By 55–64 years, regional ordering was broadly preserved but the absolute spread contracted (Northeast: 0.401; West: 0.399; South: 0.312; Midwest: 0.273). Temporal analyses demonstrated the steepest positive AAPCs in the Midwest at 35–44 years (2.59%, 95% CI 1.96–3.27) and in the South and West at 55–64 years (South: 2.02%, 95% CI: 1.87–2.18; West: 1.92%, 95% CI: 1.62–2.13) (Supplementary Table 2). In the 15–24-year group, PMRs declined significantly in the Midwest (AAPC − 2.52%, 95% CI: −3.73 to − 1.10) and Northeast (AAPC − 1.36%, 95% CI: −2.52 to − 0.40, *p* = 0.009). 

**Fig. 4:**
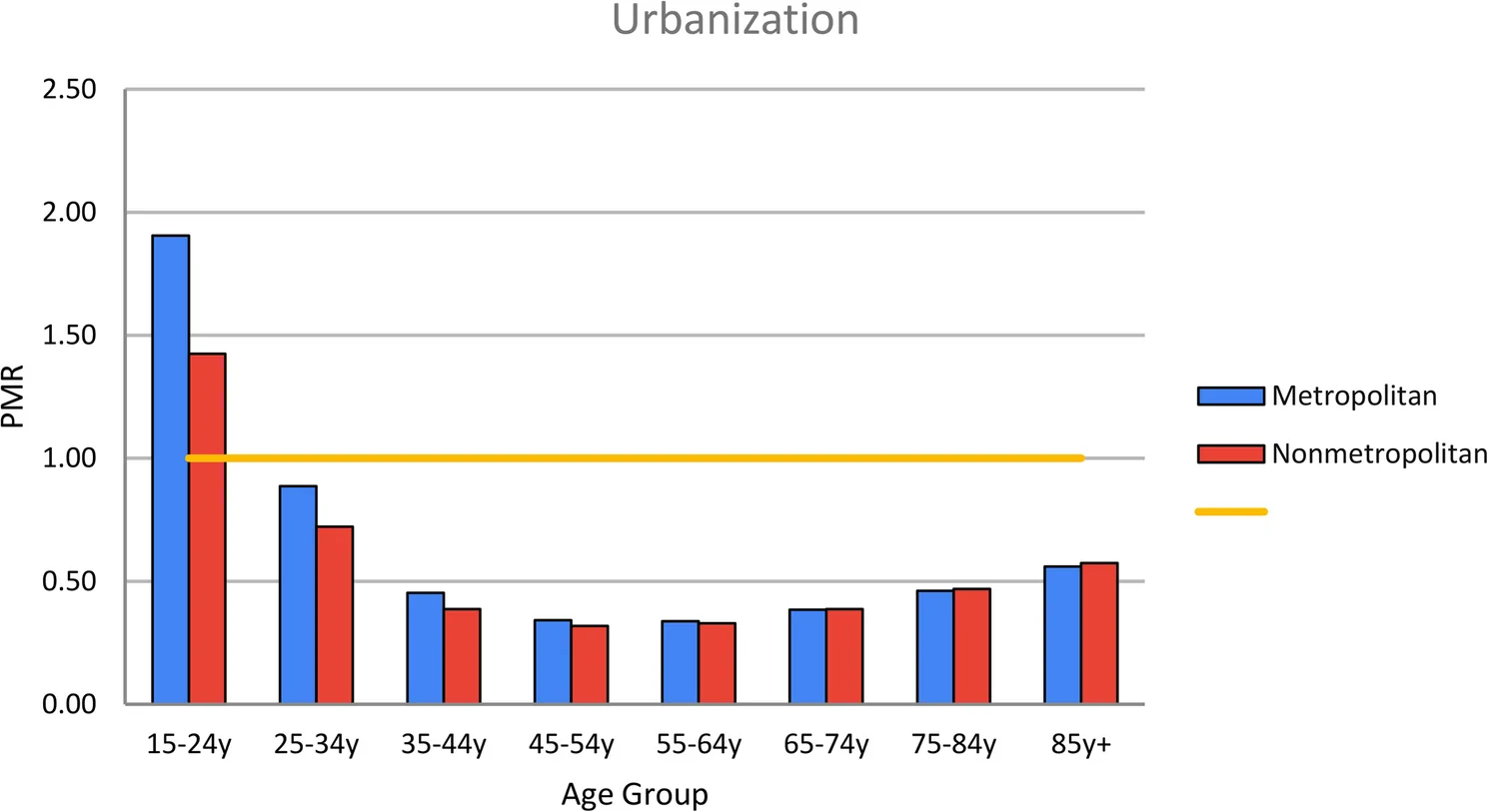
Proportional Mortality Ratio for cardiovascular co-listing on malignant neoplasm death certificates, stratified by urbanisation level and age group in the United States, 1999–2020

Metropolitan-area decedents had higher PMRs than non-metropolitan decedents at young ages (15–24 years: metropolitan 1.905, 95% CI: 1.850–1.963; non-metropolitan 1.425, 95% CI: 1.320–1.538) (Fig. 4; Supplementary Table 4). This gradient was minimal by 55–64 years (metropolitan 0.337, 95% CI: 0.336–0.339; non-metropolitan 0.329, 95% CI: 0.327–0.332) and reversed slightly at ≥ 85 years (metropolitan 0.560; non-metropolitan 0.574).

### Racial and ethnic stratification

**Fig. 5:**
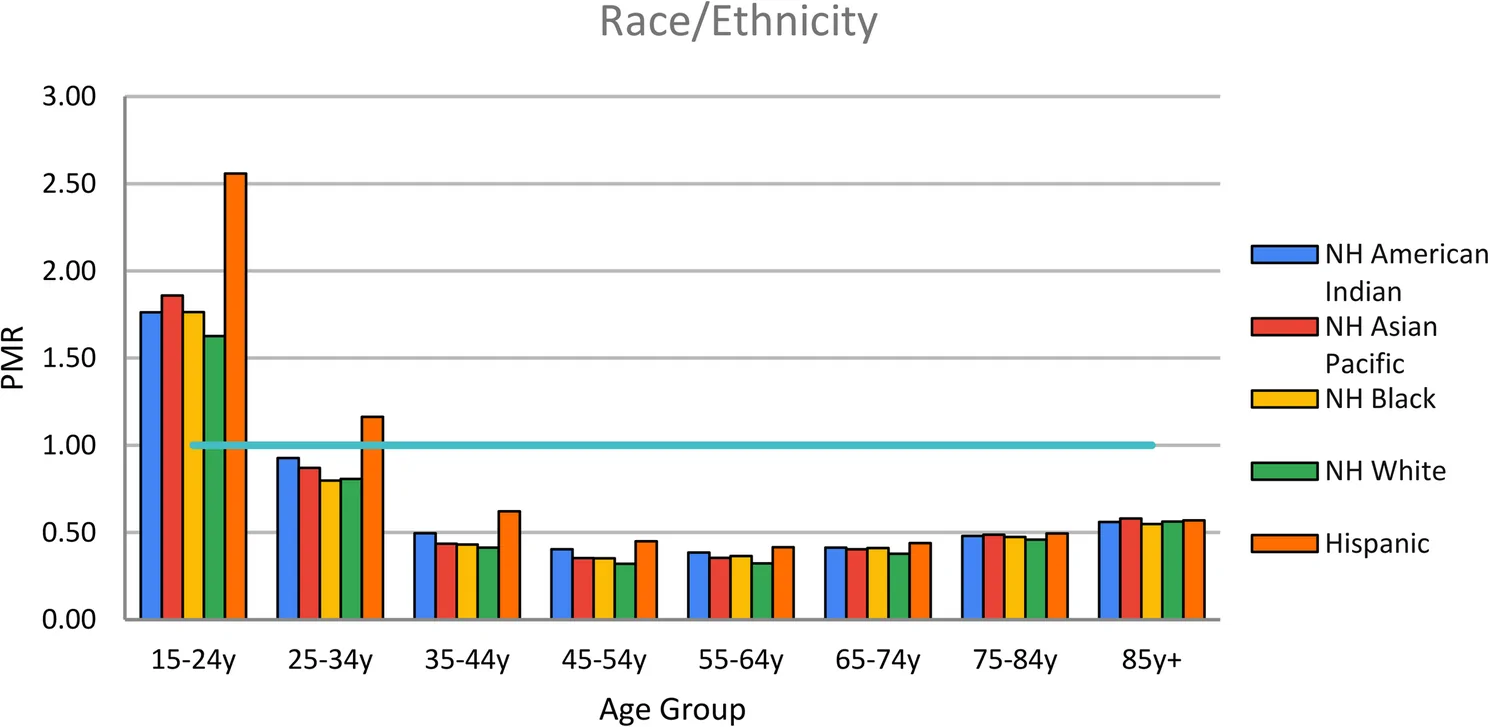
Proportional Mortality Ratio for cardiovascular co-listing on malignant neoplasm death certificates, stratified by race/ethnicity and age group in the United States, 1999–2020

Among racial and ethnic groups at 15–24 years, Hispanic or Latino decedents had the highest relative CVD co-listing (PMR 2.558, 95% CI: 2.418–2.706), followed by NH Asian or Pacific Islander (1.859, 95% CI: 1.646–2.098), NH Black (1.764, 95% CI: 1.654–1.880), NH American Indian or Alaska Native (1.763, 95% CI: 1.277–2.434), and NH White (1.626, 95% CI 1.564–1.690) (Fig. 5; Supplementary Table 6). At 55–64 years, the ranking shifted: Hispanic (0.415, 95% CI: 0.410–0.420) and NH American Indian or Alaska Native (0.385, 95% CI: 0.370–0.401) had the highest PMRs, while NH White (0.323, 95% CI: 0.322–0.324) had the lowest. By ≥ 85 years the groups converged, with PMRs ranging from 0.548 (NH Black, 95% CI: 0.543–0.554) to 0.580 (NH Asian or Pacific Islander, 95% CI: 0.570–0.590). Temporal analyses showed positive AAPCs in most race–age strata; the steepest was NH American Indian or Alaska Native at 65–74 years (AAPC 2.20%, 95% CI: 1.75–2.69) (Supplementary Table 2).

### Place of death

**Fig. 6:**
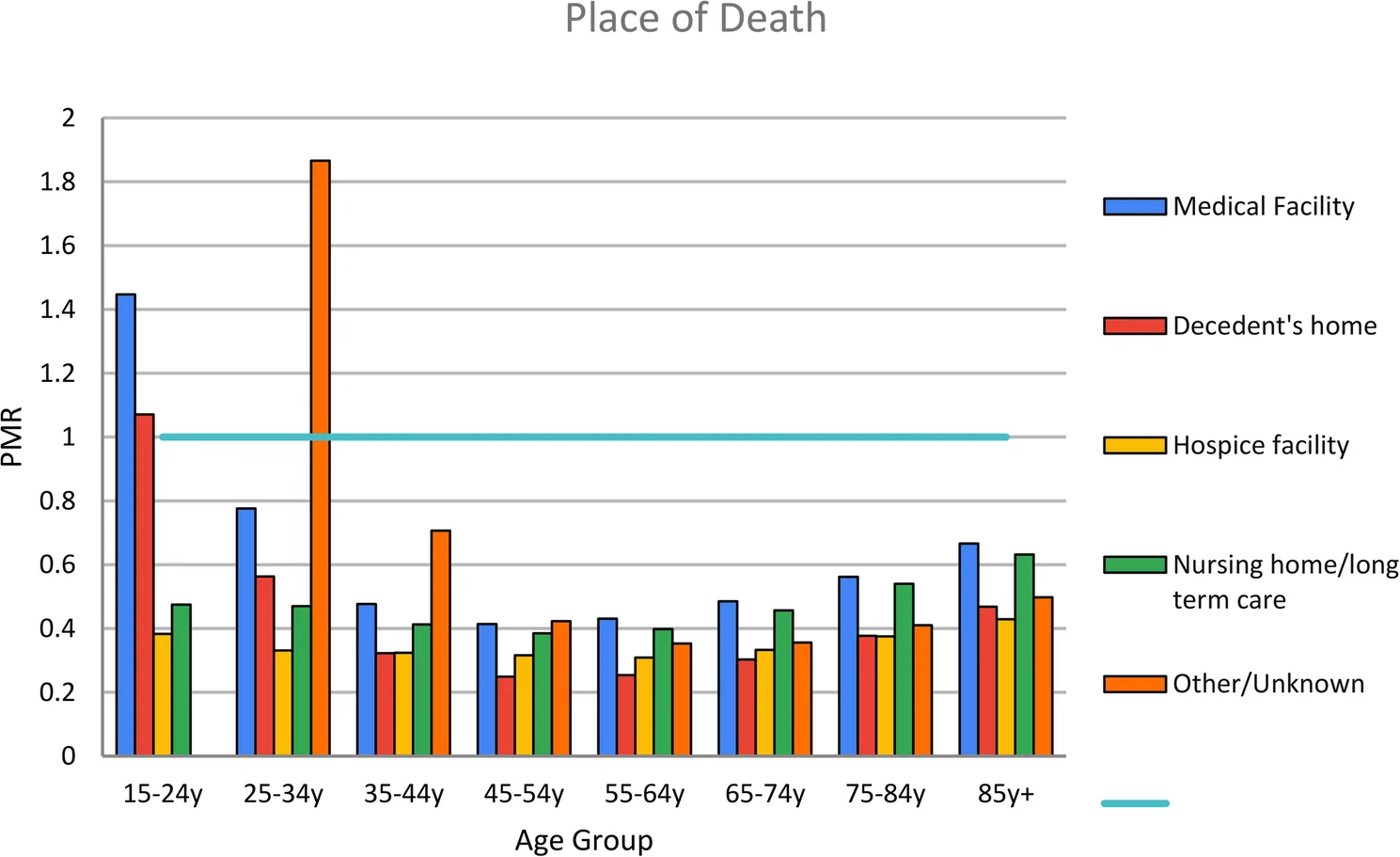
Proportional Mortality Ratio for cardiovascular co-listing on malignant neoplasm death certificates, stratified by place of death and age group in the United States, 1999–2020 *The Unknown/Other PMR value for 15-24y (5.32) was removed due to data uncertainty*

Deaths at medical facilities had the highest proportional CVD representation across all age groups (Fig. 6; Supplementary Table 7). At 15–24 years, medical facility deaths carried a PMR of 1.447; deaths at the decedent’s home showed a lower PMR (1.071); hospice deaths lower still (0.383); and nursing home deaths showed a pattern consistent with the older-adult strata (≥ 85 years: 0.540). The Other/Unknown place-of-death category exhibited an extreme value at 15–24 years (PMR 5.32) reflecting data sparsity.

### Cancer-subtype analyses

Comparative PMR and AAPC data for all three cancer subtypes are summarised in Table 1.

**Table 1:** Comparative Proportional Mortality Ratio (95% CI) and average annual percent change (AAPC) for cardiovascular co-listing by cancer site and age group in the United States, 1999–2020

Age Groups	Lung Cancer		Leukemia		Colon	
	PMR	AAPC 1999–2020 (95% CI)	PMR	AAPC 1999–2020 (95% CI)	PMR	AAPC 1999–2020 (95% CI)
15–24	1.839 (1.498–2.257)	—	2.174 (2.072–2.281)	0.92 (− 0.03 to 1.88)	1.173 (0.951–1.447)‡	—
25–34	0.893 (0.816–0.977)	1.86* (1.03–2.92)	1.177 (1.125–1.232)	0.57 (− 0.41 to 1.65)	0.651 (0.600–0.706)	0.81 (− 0.31–2.30)
35–44	0.460 (0.447–0.473)	1.90* (1.55–2.23)	0.681 (0.657–0.707)	0.77 (− 0.14–1.59)	0.412 (0.397–0.427)	0.81* (0.28–1.43)
45–54	0.400 (0.396–0.404)	1.32* (1.06–1.56)	0.545 (0.532–0.559)	0.96* (0.61–1.35)	0.374 (0.367–0.381)	0.63* (0.31–0.99)
55–64	0.425 (0.423–0.428)	1.13* (0.98–1.25)	0.533 (0.525–0.542)	1.18* (0.96–1.38)	0.418 (0.414–0.423)	0.58* (0.34–0.86)
65–74	0.469 (0.467–0.471)	1.13* (0.97–1.25)	0.551 (0.545–0.557)	1.13* (0.86–1.36)	0.504 (0.500–0.508)	0.77* (0.53–0.99)
75–84	0.486 (0.484–0.488)	1.16* (1.05–1.37)	0.584 (0.578–0.589)	1.16* (1.01–1.29)	0.586 (0.582–0.590)	0.72* (0.57–0.83)
85+	0.492 (0.489–0.495)	1.00* (0.90–1.15)	0.647 (0.641–0.653)	0.89* (0.67–1.05)	0.654 (0.650–0.659)	0.26* (0.10–0.40)

‡ Colon cancer 15–24y PMR confidence interval includes unity (95% CI 0.951–1.447); proportional CVD co-listing in this stratum did not differ significantly from the non-cancer reference population. Dashes (-) indicate suppressed or unavailable Joinpoint estimates due to sparse data

*PMR* Proportional Mortality Ratio, *AAPC* average annual percent change, *CI* confidence interval

AAPC values marked with * in supplementary tables indicate significance at *p* < 0.05

Lung cancer. Among 15–24-year decedents, lung cancer demonstrated a PMR of 1.839 (95% CI 1.498–2.257), comparable to the overall malignant-neoplasm PMR in the same age group (Supplementary Fig. 2; Supplementary Tables 8–9). PMR declined to a nadir at 45–54 years (0.400, 95% CI: 0.396–0.404) before rising to 0.492 (95% CI: 0.489–0.495) at ≥ 85 years. Male PMR at 15–24 years was 2.024 (95% CI: 1.540–2.659) compared with 1.428 (95% CI: 1.047–1.948) in females. The West region had the highest young-adult PMR (2.392, 95% CI: 1.668–3.430). The 15–24-year group had insufficient counts for temporal analysis. Significant upward temporal trends were present from 25 to 34 years onward; the largest AAPCs were at 35–44 years (1.90%, 95% CI: 1.55–2.23) and 25–34 years (1.86%, 95% CI: 1.03–2.92) and remained positive through the oldest group (≥ 85 years: AAPC 1.00%, 95% CI: 0.90–1.15).

Leukaemia. Leukaemia demonstrated the highest relative CVD co-listing of the three index cancers in young adults (15–24 years: PMR 2.174, 95% CI: 2.072–2.281) (Supplementary Fig. 3; Supplementary Tables 10–11). PMR declined to a nadir at 55–64 years (0.533, 95% CI: 0.525–0.542), then rose to 0.647 (95% CI: 0.641–0.653) at ≥ 85 years. This is the highest ≥ 85-year value among the three subtypes. Sex differences were pronounced at 15–24 years (males: 2.505, 95% CI: 2.358–2.662; females: 1.586, 95% CI: 1.467–1.715). Among racial groups, Hispanic or Latino decedents had the highest 15–24-year PMR (2.637, 95% CI: 2.419–2.874). The 15–24-year AAPC was 0.92% (95% CI: −0.03 to 1.88, *p* = 0.053), borderline non-significant; significant positive AAPCs began at 45–54 years (0.96%, 95% CI: 0.61–1.35) and peaked at 55–64 years (1.18%, 95% CI: 0.96–1.38).

Colon cancer. The 15–24-year PMR was 1.173 (95% CI: 0.951–1.447); the confidence interval crosses 1, indicating no statistically significant proportional CVD co-listing in the youngest age group, distinguishing colon cancer from both lung cancer and leukaemia at this age (Supplementary Fig. 4; Supplementary Tables 12–13). PMR declined to a nadir at 45–54 years (0.374, 95% CI: 0.367–0.381), then rose progressively to 0.654 (95% CI: 0.650–0.659) at ≥ 85 years. Male PMR at 15–24 years was 1.409 (95% CI: 1.080–1.840) while female PMR was 0.802 (95% CI: 0.571–1.127), also non-significant. Significant upward temporal trends began at 35–44 years (AAPC 0.81%, 95% CI: 0.28–1.43, *p* = 0.005) and persisted through ≥ 85 years, though the AAPC in the oldest group was the most modest of all subtypes (0.26%, 95% CI: 0.10–0.40, *p* = 0.003).

## Discussion

CVD co-listing on cancer-related death certificates was not uniform across cancer types, age groups, or demographic strata between 1999 and 2020. The reverse J-shaped age gradient, the systematic sex and regional differences, and the divergence across cancer subtypes all indicate that cardiovascular disease occupies markedly different shares of the cancer mortality profile. These are surveillance observations from death-certificate records describing proportional representation, not individual cardiovascular risk.

Prospective cohort data establish a clear biological expectation that cancer patients, given their shared cardiovascular risk factors, treatment-related cardiotoxicity, and systemic inflammatory burden, should have CVD co-listing rates exceeding those of the non-cancer population at every age. Cardiac mortality SMRs among five-year cancer survivors diagnosed at 15–19 years reach 4.2 (95% CI 3.4–5.2) and remain elevated through their 40s [4]; among 3.23 million SEER cancer survivors, CVD mortality risk was highest in those diagnosed before age 35, with an SMR of 3.93 (95% CI 3.89–3.97) in the first year after diagnosis [17]; and leukaemia survivors in the Childhood Cancer Survivor Study had hazard ratios of 5.9 for congestive heart failure and 5.0 for myocardial infarction versus siblings, with risks amplified two- to five-fold by cumulative anthracycline exposure [18]. The findings of our analysis are opposite to this expectation across most of the adult lifespan. After a genuine elevation in young adults, where PMRs exceed 1 and align with the prospective signal, values decline progressively from the mid-thirties onward, reaching a nadir of 0.336 at 55–64 years before a partial recovery at older ages. This pattern persists after cancer deaths have been removed from the non-cancer reference population, eliminating conflation of the two groups as an explanation.

The most plausible explanation for sub-unity PMRs is that CVD is systematically under-documented on cancer death certificates while simultaneously being over-attributed on non-cancer certificates at middle and older ages, with both distortions pushing the PMR below 1 regardless of the underlying biology. In a cohort of lung cancer patients receiving radical radiotherapy, baseline CVD was present in 30.9% and post-treatment cardiac hospitalisations occurred at 6.2 per 100 patient-years, yet only 5.3% of deaths were certified as primarily cardiac, with many sudden out-of-hospital deaths attributed to cancer in the palliative care involvement [19]. A hospital-based audit of 183 consecutive death certificates found that at least one comorbidity was omitted in 56% of certificates for patients under 75 and 71% for those aged 75 or older, with cardiovascular conditions among the most frequently omitted diagnoses [20]. Nationally, CVD appears on roughly one-third of cancer death certificates [21], but this figure likely underestimates true prevalence since prospective cohort studies document cardiovascular event rates in cancer survivors substantially more than what a one-third co-listing prevalence would imply [2]. Death certificate sensitivity for heart disease as the underlying cause runs around 60%, compared with 80% for malignant neoplasms [22]; while this reflects underlying-cause accuracy rather than co-listing completeness, it confirms that cardiovascular conditions are more prone to misclassification in multimorbid deaths. Provider focus groups found that the complexity of cancer documentation created substantial structural load, with many providers struggling to fit cancer history into the certificate at all, leaving little guidance on handling comorbid conditions [23]. The over-attribution problem runs in the other direction: at older ages where CVD dominates non-cancer mortality, it is frequently selected as the underlying cause on non-cancer certificates even when a more specific condition was responsible, with death certificates overestimating coronary heart disease deaths by 51% in adults aged 35 to 74 and more than doubling attribution in those aged 85 or older [24, 25]. Neither distortion reflects genuine biology. In a large English cohort of 104,028 cancer survivors, CVD mortality overtook cancer-specific mortality within 2 to 17 years of diagnosis [26], a pattern difficult to reconcile with sub-unity PMRs.

These documentation effects are not constant. As age progresses, denominator inflation from CVD over-attribution on non-cancer certificates compresses the PMR even when absolute cancer-decedent co-listing is unchanged. In young adults, background cardiovascular mortality in the non-cancer population is near zero, so an unexpected cardiac event in a young cancer patient is more likely to be recorded explicitly. The reverse J-shape therefore reflects both a genuine biological signal in younger decedents, supported by prospective cohort data [4, 5], and a denominator inflation effect at older ages that this dataset cannot disentangle. Documentation completeness also varies by place of death and certifier type, contributing to the place-of-death PMR gradient observed in this study [27].

Across the three index cancers, leukaemia demonstrated the highest proportional CVD co-listing in young adults and the highest PMR at 85 years or older, suggesting cardiovascular disease occupies a consistently prominent position within leukaemia-related mortality across the lifespan. Lung cancer closely mirrored the overall malignant neoplasm pattern at younger ages but showed a shallower rise at older ages, consistent with a disease in which rapid progression may reduce the opportunity for comorbid conditions to be documented alongside the primary malignancy [19]. Colon cancer showed the most attenuated pattern, with a 15–24-year PMR of 1.17 whose confidence interval crosses unity, distinguishing it from both leukaemia and lung cancer at this age. These differences may reflect varying degrees of true cardiovascular comorbidity, differential cardiotoxicity of treatment regimens, or differences in disease tempo; death-certificate data cannot separate these contributions.

Temporally, PMR increased significantly across all adult age groups from 1999 to 2020, with the sole exception of the 15–24-year group. Improved cancer survival exposes a larger population to cumulative cardiovascular consequences of cancer therapies [28], and the formal establishment of cardio-oncology as a clinical subspecialty with systematic surveillance protocols may have contributed to more consistent CVD documentation in oncology settings over this period [29]. Simultaneously, the declining CVD co-listing proportion among non-cancer decedents, reflected in consistently negative C-A/D-B AAPCs across adult strata, mirrors improvements in cardiovascular mortality in the non-cancer population [30] and mathematically amplifies the PMR even when absolute cancer-decedent co-listing rises only modestly. The consistency of upward trends across sex, race/ethnicity, and census region argues against a purely administrative explanation and points toward a genuine shift in how cardiovascular disease is recognised and recorded in the context of cancer mortality lately. Prior work described the absolute burden of CVD co-listing on cancer death certificates using age-adjusted rates [21]; the present analysis adds a proportional comparison against non-cancer certificates, exposing the relative suppression of cardiovascular documentation across most of the adult lifespan, something rate-based approaches cannot show.

Racial and regional patterns warrant caution in interpretation. Hispanic or Latino decedents demonstrated the highest proportional CVD co-listing across multiple younger age strata, and Non-Hispanic American Indian or Alaska Native decedents showed the steepest temporal increases. PMRs in these groups reflect both true disease burden and death-certificate ascertainment quality: race misclassification on death certificates reached 40% for the American Indian or Alaska Native population in the most recent national validation study, while remaining substantially lower for Black and White decedents [31]. Racial and ethnic minoritised groups also face higher cardiovascular mortality, increased cardiotoxicity risks during cancer treatment, more advanced-stage cancer diagnoses at presentation, and greater cardiovascular risk factor burden, all of which add further complexity to interpreting these PMR patterns [32]. Regional differences in cancer case mix, socioeconomic composition, access to academic cancer centers, and coding practice all plausibly contribute to the geographic gradient. Demographic and regional PMR differences should be read as ecological observations blending true epidemiologic variation with patterned ascertainment differences; they are not adjusted risk estimates.

Simulation analyses of death-certificate data found that both collider bias, arising from conditioning on death, and reporting bias, arising from selective omission of secondary conditions, attenuated observed associations toward the null, with reporting bias the larger effect, ranging from 4.7-fold to 64-fold attenuation compared with 1.7-fold to 9.3-fold for collider bias [33]. Minhas et al., using the same CDC WONDER multiple cause-of-death files, found that underlying-cause-only methods capture only 53% of heart disease deaths versus 91% of malignant neoplasm deaths [34], quantifying the asymmetry empirically. Together, these analyses suggest the PMRs reported here understate rather than overstate cardiovascular co-listing in cancer mortality.

The consistent over-representation of CVD among younger cancer decedents, particularly those with leukaemia, and the demographic disparities identified here align with a growing body of evidence supporting cardiovascular surveillance as a component of cancer care [32]. Sub-unity PMRs in middle-aged and older patients, read alongside the certifier behaviour evidence, make a direct case for improving death-certificate practice in oncology settings, which is recording CVD as a contributing cause alongside the underlying cancer diagnosis would produce mortality statistics that more accurately reflect the true multimorbidity burden and generate more reliable surveillance data for cardio-oncology research. These findings do not establish which clinical interventions are warranted; that determination requires the prospective data that these surveillance signals are designed to motivate.

Death-certificate reporting heterogeneity modifies the observed PMR magnitude rather than generating the signal from nothing. Cancer and CVD share upstream biological drivers including chronic inflammation, metabolic dysregulation, and clonal haematopoiesis [35, 36], and the available evidence suggests our estimates are conservative throughout. What the PMR cannot do is distinguish between pre-existing cardiovascular disease, treatment-related injury, and late survivorship cardiotoxicity. It identifies populations and age groups where CVD occupies a disproportionate share of the cancer mortality record; it tells us where to look, not what is causing the signal.

## Limitations

1. *Ecological and cross-sectional design*. Analyses use aggregate death counts without individual follow-up. PMRs describe group-level proportional patterns and support no causal inference.
2. *No individual-level clinical data*. CDC WONDER contains no information on cancer stage, treatment regimen, time since diagnosis, or comorbidity burden. The PMR cannot distinguish cardiovascular disease present before cancer diagnosis from disease arising during treatment or survivorship. This is a constraint of national death-certificate surveillance rather than a correctable methodological gap.
3. *Multiple comparisons*. PMRs are reported across many strata. The analysis is exploratory and no corrections for multiple comparisons were applied; stratum-level differences should not be treated as confirmatory.
4. *Temporal changes in certification and coding*. ICD-10 conventions, certifier behaviour, and clinical recognition of cardiovascular toxicity in oncology all evolved over 1999–2020. Observed temporal trends in PMR reflect a mixture of true epidemiological change and shifting documentation practice that cannot be separated in this dataset.
5. *Race and ethnicity misclassification*. Race and Hispanic origin are subject to systematic misclassification on death certificates, most markedly for Non-Hispanic American Indian or Alaska Native decedents. Demographic PMRs for these groups should be interpreted with caution.
6. *Suppression of small cells*. CDC WONDER suppresses cells with fewer than ten deaths. Several demographic and age-group strata, notably Non-Hispanic American Indian or Alaska Native decedents in the youngest age groups, were partially or fully suppressed, limiting completeness of subgroup analyses.

## Conclusion

National death-certificate surveillance from 1999 to 2020 reveals that cardiovascular co-listing on cancer-related death certificates is neither random nor uniform. It follows a consistent age pattern, peaks in leukaemia among the three index cancers, and has risen across nearly all adult age groups over the study period. The elevated PMRs in young adult cancer decedents are consistent with what prospective cohort data tell us about cardiovascular risk in this population. The sub-unity PMRs in middle-aged and older adults, by contrast, almost certainly reflect documentation behaviour rather than biology. The gap between true burden and recorded burden is the central finding of this study, and it points to two practical priorities. Cardiovascular surveillance in young cancer patients, particularly those with haematological malignancies, is supported by the mortality record. Systematic improvement in comorbidity documentation on cancer death certificates would narrow the gap between what the surveillance record shows and what the clinical literature tells us is happening and would make national mortality data substantially more useful for cardio-oncology research and planning.

## Supplementary information

Supplementary Material 1.

## Data Availability

All data used in this study are publicly available through the Centers for Disease Control and Prevention Wide-ranging Online Data for Epidemiologic Research (CDC WONDER) database. The authors obtained access in accordance with the CDC’s data use guidelines.

## Abbreviations

CVD: Cardiovascular disease
PMR: Proportional Mortality Ratio
SMR: Standardised mortality ratio
CI: Confidence interval
SE: Standard error
SEER: Surveillance, Epidemiology, and End Results
CDC: Centers for Disease Control
WONDER: Wide-ranging ONline Data for Epidemiologic Research
MCD: Multiple Cause-of-Death
ICD-10: International Classification of Diseases, Tenth Revision
STROBE: Strengthening the Reporting of Observational Studies in Epidemiology
NH: Non-Hispanic
APC: Annual percent change
AAPC: Average annual percent change
WBIC: Weighted Bayesian Information Criterion
US: United States
