# Supplementary material for: Heterogeneity in proportional cardiovascular co-listing on cancer death certificates in the United States: a national multiple cause-of-death analysis, 1999–2020
Source: Cardiooncology. 2026 Jun 12;12:124. doi: 10.1186/s40959-026-00525-8 (PMC13651163; doi:10.1186/s40959-026-00525-8)
Supplement: Supplementary file 1 [file 40959_2026_525_MOESM1_ESM.docx]

**Supplementary Material**

**Calculations and Worked Example**

***Step 1 — CVD Co-listing Proportion in Cancer Deaths (A/B)***

The first step quantifies the proportion of cancer-related death certificates that also list cardiovascular disease (CVD) anywhere on the certificate:

CVD co-listing proportion in cancer deaths = A / B

where **A** is the number of death certificates listing both a malignant neoplasm (ICD-10: C00–C97) and CVD (ICD-10: I00–I99) anywhere across all cause-of-death fields, and **B** is the total number of death certificates listing a malignant neoplasm anywhere on the certificate. Both A and B are derived from CDC WONDER Multiple Cause-of-Death Public Use files using an any-mention framework, meaning all conditions recorded in Parts I and II of the death certificate are included regardless of whether they were selected as the underlying cause.

***Step 2 — CVD Co-listing Proportion in Non-Cancer Deaths [(C−A) / (D−B)]***

The reference proportion is calculated from death certificates that do not list cancer. To avoid double-counting, cancer-related deaths are excluded from both the CVD numerator and the all-cause denominator:

CVD co-listing proportion in non-cancer deaths = (C − A) / (D − B)

where **C** is the total number of death certificates in the full population listing CVD anywhere on the certificate, and **D** is total all-cause mortality. Subtracting A from C and B from D removes cancer-related deaths from both the CVD count and the all-cause denominator, yielding a CVD co-listing proportion exclusive to the non-cancer decedent population. This adjustment is essential: using C/D without the subtraction would retain cancer deaths in the reference group, inflating the denominator at older ages where cancer is a large share of total mortality and compressing the PMR.

***Step 3 — Proportional Mortality Ratio (PMR)***

The PMR is derived by dividing the CVD co-listing proportion in cancer deaths by the corresponding proportion in non-cancer deaths:

PMR = (A / B) ÷ [(C − A) / (D − B)]

A PMR greater than 1 indicates that CVD is proportionally more frequently co-listed on cancer-related death certificates than on non-cancer certificates. A PMR less than 1 indicates the reverse. The PMR is a measure of relative proportional representation on death certificates and does not estimate incidence, absolute mortality risk, or causal effect; it should not be interpreted as a hazard ratio or relative risk.

***Step 4 — Confidence Intervals (Log-Scale Delta Method)***

Standard errors and 95% confidence intervals were derived using the log-scale delta method. The standard error of the natural log of the PMR is:

SE[ln(PMR)] = √(1/A + 1/(C−A) + 1/B + 1/(D−B))

The 95% confidence interval on the PMR scale is:

95% CI = [exp(ln(PMR) − 1.96 × SE), exp(ln(PMR) + 1.96 × SE)]

This Poisson-based delta method approximation treats each death count (A, C−A, B, D−B) as an independent Poisson observation and is consistent with variance estimation in proportional mortality analyses. All calculations were performed in Microsoft Excel (Microsoft Corporation, Redmond, WA).

Because the total death counts underlying each PMR estimate are derived from the full US national mortality record across 22 years, sample sizes are very large and resulting confidence intervals are narrow. This reflects high statistical precision of the administrative record rather than freedom from systematic error. Residual uncertainty from misclassification, reporting bias, and collider bias is addressed in the Limitations section of the main manuscript.

**Table A. 2×2 Contingency Table**

|  | **CVD Present (ICD-10: I00–I99)** | **CVD Absent** | **Total** | **Proportion** |
| --- | --- | --- | --- | --- |
| **Cancer Deaths** (malignant neoplasm listed anywhere on certificate) | **A** Deaths listing both CVD and malignant neoplasm | B − A | **B** All cancer deaths | **A / B** CVD co-listing proportion in cancer deaths |
| **Non-Cancer Deaths** (all death certificates not listing cancer) | **C − A** Non-cancer deaths listing CVD on certificate | (D − B) − (C − A) | **D − B** All non-cancer deaths | **(C − A) / (D − B)** CVD co-listing proportion in non-cancer deaths |
| **Total** | **C** All CVD Deaths | D - C | **D** All deaths |  |
| **PMR = (A / B) ÷ [(C − A) / (D − B)]** \| A PMR > 1: CVD proportionally more frequent on cancer certificates. A PMR < 1: the reverse. | | | | |

**Table B. Worked Example: Age Group 15–24 Years (Aggregate 1999–2020)**

| **Age Group 15–24y (Aggregate 1999–2020)** | **CVD Present** | **CVD Absent** | **Total** | **Proportion** |
| --- | --- | --- | --- | --- |
| **Cancer Deaths** | **A = 6,562** | B − A | **B = 36,257** | A/B = 6,562/36,257 = 0.181 |
| **Non-Cancer Deaths** | **C−A = 65,370** | (D−B) − (C−A) | **D−B = 660,222** | (C−A)/(D−B) = 65,370/660,222 = 0.099 |
| **Total** | **C = 71,932** | D – C | **D = 696,479** |  |
| **PMR = 0.181 ÷ 0.099 = 1.828** | | | | |

*.Source: CDC WONDER Multiple Cause-of-Death Public Use Files, 1999–2020. ICD-10: malignant neoplasms = C00–C97; CVD = I00–I99. Aggregate counts represent the sum of annual death certificate records across all 22 study years.*

**Table C. Confidence Interval Derivation**

| **Step** | **Formula** | **Applied to 15–24y Example** |
| --- | --- | --- |
| **1** | **Standard error of ln(PMR)** SE = √(1/A + 1/(C−A) + 1/B + 1/(D−B)) | SE = √(1/6,562 + 1/65,370 + 1/36,257 + 1/660,222), **SE = √(0.0001967) = 0.0140** |
| **2** | **Log-scale 95% CI bounds** ln(PMR) ± 1.96 × SE | ln(**1.828**) = 0.6032 ± 1.96 × 0.0140 = 0.6537 ± 0.02744 **[0.5758, 0.6306]** |
| **3** | **Back-transform to PMR scale** 95% CI = [exp(lower), exp(upper)] | [exp(0.5758), exp(0.6306)] = [1.870, 1.976] **PMR = 1.828 (95% CI: 1.778–1.879)** |

*The standard error is computed on the natural log scale using the Poisson-based delta method, treating each of the four independent cell counts (A, C−A, B, D−B) as a Poisson observation. Confidence intervals are back-transformed from the log scale to avoid the asymmetry of direct normal-approximation intervals. This approach is consistent with variance estimation in occupational proportional mortality analyses.*

***Abbreviations***

PMR, Proportional Mortality Ratio; CVD, cardiovascular disease; ICD-10, International Classification of Diseases, 10th Revision; CDC WONDER, Centers for Disease Control and Prevention Wide-Ranging Online Data for Epidemiologic Research; CI, confidence interval; SE, standard error; A, deaths listing both cancer and CVD; B, all cancer deaths; C, all deaths listing CVD in the full population; D, all-cause deaths in the full population.

**Legend**

**Supplementary Figure 1.** Temporal trends in the Proportional Mortality Ratio for cardiovascular co-listing on malignant neoplasm death certificates, stratified by age group in the United States, 1999–2020

**Supplementary Figure 2.** Proportional Mortality Ratio for cardiovascular co-listing on lung cancer death certificates, stratified by age group in the United States, 1999–2020

**Supplementary Figure 3.** Proportional Mortality Ratio for cardiovascular co-listing on leukaemia death certificates, stratified by age group in the United States, 1999–2020

**Supplementary Figure 4.** Proportional Mortality Ratio for cardiovascular co-listing on colon cancer death certificates, stratified by age group in the United States, 1999–2020

**Supplementary Table 1.** Death counts and Proportional Mortality Ratio (95% CI) for cardiovascular co-listing on malignant neoplasm death certificates, stratified by age group and demographic subgroup in the United States, 1999–2020

**Supplementary Table 2.** Average annual percent change (AAPC) in the Proportional Mortality Ratio for cardiovascular co-listing on malignant neoplasm death certificates, by age group and demographic subgroup in the United States, 1999–2020

**Supplementary Table 3.** Annual CVD co-listing proportions in malignant neoplasm death certificates (A/B) and non-cancer death certificates [(C−A)/(D−B)], by age group in the United States, 1999–2020

**Supplementary Table 4.** Proportional Mortality Ratio (95% CI) for cardiovascular co-listing on malignant neoplasm death certificates, stratified by sex and urbanisation level in the United States, 1999–2020

**Supplementary Table 5.** Proportional Mortality Ratio (95% CI) for cardiovascular co-listing on malignant neoplasm death certificates, stratified by US Census region in the United States, 1999–2020

**Supplementary Table 6.** Proportional Mortality Ratio (95% CI) for cardiovascular co-listing on malignant neoplasm death certificates, stratified by race and ethnicity in the United States, 1999–2020

**Supplementary Table 7.** Proportional Mortality Ratio (95% CI) for cardiovascular co-listing on malignant neoplasm death certificates, stratified by place of death in the United States, 1999–2020

**Supplementary Table 8.** Death counts and Proportional Mortality Ratio (95% CI) for cardiovascular co-listing on lung cancer death certificates, stratified by age group and demographic subgroup in the United States, 1999–2020

**Supplementary Table 9.** Average annual percent change (AAPC) in the Proportional Mortality Ratio for cardiovascular co-listing on lung cancer death certificates, by age group and demographic subgroup in the United States, 1999–2020

**Supplementary Table 10.** Death counts and Proportional Mortality Ratio (95% CI) for cardiovascular co-listing on leukaemia death certificates, stratified by age group and demographic subgroup in the United States, 1999–2020

**Supplementary Table 11.** Average annual percent change (AAPC) in the Proportional Mortality Ratio for cardiovascular co-listing on leukaemia death certificates, by age group and demographic subgroup in the United States, 1999–2020

**Supplementary Table 12.** Death counts and Proportional Mortality Ratio (95% CI) for cardiovascular co-listing on colon cancer death certificates, stratified by age group and demographic subgroup in the United States, 1999–2020

**Supplementary Table 13.** Average annual percent change (AAPC) in the Proportional Mortality Ratio for cardiovascular co-listing on colon cancer death certificates, by age group and demographic subgroup in the United States, 1999–2020

**Supplemental Figure 1:** Temporal trends in the Proportional Mortality Ratio for cardiovascular co-listing on malignant neoplasm death certificates, stratified by age group in the United States, 1999–2020

Overall Ratio

PMR

| Age Group | Rate Overall |  |
| --- | --- | --- |
| 15-24y | 1.83866 | 1 |
| 25-34y | 0.892563 | 1 |
| 35-44y | 0.459766 | 1 |
| 45-54y | 0.399842 | 1 |
| 55-64y | 0.425412 | 1 |
| 65-74y | 0.468785 | 1 |
| 75-84y | 0.485798 | 1 |
| 85y+ | 0.492349 | 1 |

**Supplementary Figure 2:** Proportional Mortality Ratio for cardiovascular co-listing on lung cancer death certificates, stratified by age group in the United States, 1999–2020

**Supplementary Figure 3:** Proportional Mortality Ratio for cardiovascular co-listing on leukaemia death certificates, stratified by age group in the United States, 1999–2020

Overall Ratio

PMR

| Age Group | Rate Overall |  |
| --- | --- | --- |
| 15-24y | 1.173 | 1 |
| 25-34y | 0.651 | 1 |
| 35-44y | 0.412 | 1 |
| 45-54y | 0.374 | 1 |
| 55-64y | 0.418 | 1 |
| 65-74y | 0.504 | 1 |
| 75-84y | 0.586 | 1 |
| 85y+ | 0.654 | 1 |

**Supplementary Figure 4:** Proportional Mortality Ratio for cardiovascular co-listing on colon cancer death certificates, stratified by age group in the United States, 1999–2020

| **Supplementary Table 1: Death counts and Proportional Mortality Ratio (95% CI) for cardiovascular co-listing on malignant neoplasm death certificates, stratified by age group and demographic subgroup in the United States, 1999–2020** | | | | | | | |
| --- | --- | --- | --- | --- | --- | --- | --- |
| **Deaths and Proportional Mortality Ratio (95% CI)** | | | | | | | |
| **Cohort** | **Cancer and CVD Deaths (A)** | **Total Cancer Deaths (B)** | **Total CVD Deaths (C-A)** | **Total Non-Cancer Deaths (D)** | **A/B** | **C/D** | **PMR (95% CI)** |
| **15-24 Age Group** | | | | | | | |
| Overall | 6562 | 36257 | 65370 | 660222 | 0.181 | 0.099 | 1.828 (1.778-1.879) |
| Female | 2591 | 14455 | 23123 | 168416 | 0.179 | 0.137 | 1.306 (1.249-1.364) |
| Male | 3971 | 21802 | 42247 | 491806 | 0.182 | 0.086 | 2.12 (2.047-2.196) |
| NH American Indian | 44 | 321 | 891 | 11460 | 0.137 | 0.078 | 1.763 (1.277-2.434) |
| NH Asian Pacific | 377 | 1629 | 1977 | 15877 | 0.231 | 0.125 | 1.859 (1.646-2.098) |
| NH Black | 1194 | 5880 | 17311 | 150316 | 0.203 | 0.115 | 1.764 (1.654-1.88) |
| NH White | 3210 | 20636 | 35313 | 369124 | 0.156 | 0.096 | 1.626 (1.564-1.69) |
| Hispanic | 1727 | 7706 | 9721 | 110960 | 0.224 | 0.088 | 2.558 (2.418-2.706) |
| Northeast | 1462 | 6118 | 9868 | 93104 | 0.239 | 0.106 | 2.255 (2.122-2.396) |
| Midwest | 895 | 7632 | 13430 | 144097 | 0.117 | 0.093 | 1.258 (1.171-1.351) |
| South | 2172 | 13369 | 28229 | 279205 | 0.162 | 0.101 | 1.607 (1.533-1.684) |
| West | 2033 | 9138 | 13843 | 143816 | 0.222 | 0.096 | 2.311 (2.196-2.433) |
| Metro | 5763 | 30782 | 53108 | 540515 | 0.187 | 0.098 | 1.905 (1.85-1.963) |
| Non metro | 799 | 5475 | 12262 | 119707 | 0.146 | 0.102 | 1.425 (1.32-1.538) |
| **25-34 Age Group** | | | | | | | |
| Overall | 13987 | 84925 | 183682 | 958118 | 0.165 | 0.192 | 0.859 (0.843-0.875) |
| Female | 6929 | 43307 | 66281 | 274566 | 0.160 | 0.241 | 0.663 (0.645-0.681) |
| Male | 7058 | 41618 | 117401 | 683552 | 0.170 | 0.172 | 0.987 (0.962-1.013) |
| NH American Indian | 113 | 704 | 3134 | 18095 | 0.161 | 0.173 | 0.927 (0.757-1.134) |
| NH Asian Pacific | 835 | 4139 | 5002 | 21588 | 0.202 | 0.232 | 0.871 (0.803-0.944) |
| NH Black | 2857 | 14922 | 51333 | 213918 | 0.191 | 0.240 | 0.798 (0.766-0.831) |
| NH White | 7209 | 50670 | 99350 | 563264 | 0.142 | 0.176 | 0.807 (0.786-0.828) |
| Hispanic | 2931 | 14296 | 24336 | 138052 | 0.205 | 0.176 | 1.163 (1.115-1.213) |
| Northeast | 3215 | 14088 | 27093 | 148187 | 0.228 | 0.183 | 1.248 (1.199-1.3) |
| Midwest | 1889 | 17867 | 37131 | 200611 | 0.106 | 0.185 | 0.571 (0.544-0.6) |
| South | 4870 | 33217 | 81183 | 409401 | 0.147 | 0.198 | 0.739 (0.717-0.763) |
| West | 4013 | 19753 | 38275 | 199919 | 0.203 | 0.191 | 1.061 (1.024-1.1) |
| Metro | 12124 | 72200 | 151277 | 798320 | 0.168 | 0.189 | 0.886 (0.869-0.904) |
| Non metro | 1863 | 12725 | 32405 | 159798 | 0.146 | 0.203 | 0.722 (0.687-0.759) |
| **35-44 Age Group** | | | | | | | |
| Overall | 47969 | 296962 | 543345 | 1486387 | 0.162 | 0.366 | 0.442 (0.437-0.446) |
| Female | 26283 | 166815 | 189759 | 491119 | 0.158 | 0.386 | 0.408 (0.402-0.414) |
| Male | 21686 | 130147 | 353586 | 995268 | 0.167 | 0.355 | 0.469 (0.462-0.476) |
| NH American Indian | 325 | 2130 | 7806 | 25341 | 0.153 | 0.308 | 0.495 (0.44-0.558) |
| NH Asian Pacific | 2550 | 12799 | 13515 | 29586 | 0.199 | 0.457 | 0.436 (0.416-0.457) |
| NH Black | 10273 | 53102 | 142842 | 317853 | 0.193 | 0.449 | 0.43 (0.421-0.44) |
| NH White | 27129 | 192627 | 318687 | 936174 | 0.141 | 0.340 | 0.414 (0.408-0.419) |
| Hispanic | 7554 | 35636 | 58527 | 171409 | 0.212 | 0.341 | 0.621 (0.605-0.638) |
| Northeast | 11373 | 52204 | 82930 | 233648 | 0.218 | 0.355 | 0.614 (0.601-0.627) |
| Midwest | 6948 | 64160 | 112043 | 307989 | 0.108 | 0.364 | 0.298 (0.29-0.305) |
| South | 17166 | 119408 | 241162 | 641318 | 0.144 | 0.376 | 0.382 (0.376-0.389) |
| West | 12482 | 61190 | 107210 | 303432 | 0.204 | 0.353 | 0.577 (0.566-0.589) |
| Metro | 40974 | 249506 | 444379 | 1226476 | 0.164 | 0.362 | 0.453 (0.448-0.458) |
| Non metro | 6995 | 47456 | 98966 | 259911 | 0.147 | 0.381 | 0.387 (0.377-0.397) |
| **45-54 Age Group** | | | | | | | |
| Overall | 190288 | 1057619 | 1498419 | 2812063 | 0.180 | 0.533 | 0.338 (0.336-0.339) |
| Female | 91745 | 526373 | 501042 | 957406 | 0.174 | 0.523 | 0.333 (0.33-0.336) |
| Male | 98543 | 531246 | 997377 | 1854657 | 0.185 | 0.538 | 0.345 (0.342-0.347) |
| NH American Indian | 1310 | 7174 | 16689 | 36927 | 0.183 | 0.452 | 0.404 (0.38-0.43) |
| NH Asian Pacific | 7576 | 34145 | 32328 | 51554 | 0.222 | 0.627 | 0.354 (0.344-0.364) |
| NH Black | 38774 | 178471 | 349353 | 565958 | 0.217 | 0.617 | 0.352 (0.348-0.356) |
| NH White | 122360 | 750181 | 962908 | 1890237 | 0.163 | 0.509 | 0.32 (0.318-0.322) |
| Hispanic | 19674 | 84948 | 130823 | 254198 | 0.232 | 0.515 | 0.45 (0.442-0.458) |
| Northeast | 44281 | 189490 | 238717 | 449254 | 0.234 | 0.531 | 0.44 (0.435-0.445) |
| Midwest | 31435 | 240243 | 318222 | 596834 | 0.131 | 0.533 | 0.245 (0.242-0.248) |
| South | 69983 | 425741 | 649671 | 1198020 | 0.164 | 0.542 | 0.303 (0.301-0.306) |
| West | 44589 | 202145 | 291809 | 567955 | 0.221 | 0.514 | 0.429 (0.425-0.434) |
| Metro | 158220 | 872957 | 1220843 | 2302610 | 0.181 | 0.530 | 0.342 (0.34-0.344) |
| Non metro | 32068 | 184662 | 277576 | 509453 | 0.174 | 0.545 | 0.319 (0.315-0.323) |
| **55-64 Age Group** | | | | | | | |
| Overall | 533148 | 2446534 | 2880808 | 4438505 | 0.218 | 0.649 | 0.336 (0.335-0.337) |
| Female | 225042 | 1097885 | 1014334 | 1620489 | 0.205 | 0.626 | 0.327 (0.326-0.329) |
| Male | 308106 | 1348649 | 1866474 | 2818016 | 0.228 | 0.662 | 0.345 (0.343-0.346) |
| NH American Indian | 3453 | 15174 | 25835 | 43764 | 0.228 | 0.590 | 0.385 (0.37-0.401) |
| NH Asian Pacific | 15838 | 62222 | 60500 | 84165 | 0.255 | 0.719 | 0.354 (0.347-0.361) |
| NH Black | 94541 | 360762 | 571730 | 797069 | 0.262 | 0.717 | 0.365 (0.362-0.368) |
| NH White | 378436 | 1855976 | 1997909 | 3165194 | 0.204 | 0.631 | 0.323 (0.322-0.324) |
| Hispanic | 39216 | 146098 | 211575 | 327314 | 0.268 | 0.646 | 0.415 (0.41-0.42) |
| Northeast | 116493 | 438627 | 469658 | 709830 | 0.266 | 0.662 | 0.401 (0.398-0.404) |
| Midwest | 98132 | 561280 | 620856 | 968045 | 0.175 | 0.641 | 0.273 (0.271-0.275) |
| South | 198618 | 977943 | 1231579 | 1890036 | 0.203 | 0.652 | 0.312 (0.31-0.313) |
| West | 119905 | 468684 | 558715 | 870594 | 0.256 | 0.642 | 0.399 (0.396-0.402) |
| Metro | 433997 | 1984755 | 2315330 | 3571528 | 0.219 | 0.648 | 0.337 (0.336-0.339) |
| Non metro | 99151 | 461779 | 565478 | 866977 | 0.215 | 0.652 | 0.329 (0.327-0.332) |
| **65-74 Age Group** | | | | | | | |
| Overall | 967400 | 3610827 | 4469361 | 6423461 | 0.268 | 0.696 | 0.385 (0.384-0.386) |
| Female | 398201 | 1602068 | 1846393 | 2746830 | 0.249 | 0.672 | 0.37 (0.368-0.371) |
| Male | 569199 | 2008759 | 2622968 | 3676631 | 0.283 | 0.713 | 0.397 (0.396-0.399) |
| NH American Indian | 5152 | 18524 | 31087 | 46218 | 0.278 | 0.673 | 0.413 (0.4-0.428) |
| NH Asian Pacific | 24544 | 80545 | 99229 | 131371 | 0.305 | 0.755 | 0.403 (0.397-0.41) |
| NH Black | 130999 | 420747 | 673352 | 889072 | 0.311 | 0.757 | 0.411 (0.408-0.414) |
| NH White | 746906 | 2901437 | 3362250 | 4934270 | 0.257 | 0.681 | 0.378 (0.377-0.379) |
| Hispanic | 57164 | 181283 | 287034 | 399670 | 0.315 | 0.718 | 0.439 (0.434-0.444) |
| Northeast | 209063 | 666835 | 782109 | 1087509 | 0.314 | 0.719 | 0.436 (0.433-0.438) |
| Midwest | 193213 | 836922 | 982897 | 1443518 | 0.231 | 0.681 | 0.339 (0.337-0.341) |
| South | 350100 | 1407715 | 1846229 | 2678775 | 0.249 | 0.689 | 0.361 (0.359-0.362) |
| West | 215024 | 699355 | 858126 | 1213659 | 0.315 | 0.718 | 0.435 (0.432-0.437) |
| Metro | 776507 | 2897225 | 3548706 | 5091017 | 0.268 | 0.697 | 0.385 (0.383-0.386) |
| Non metro | 190893 | 713602 | 920655 | 1332444 | 0.268 | 0.691 | 0.387 (0.385-0.389) |
| **75-84 Age Group** | | | | | | | |
| Overall | 1280687 | 3956872 | 7530951 | 10766350 | 0.324 | 0.699 | 0.463 (0.462-0.464) |
| Female | 559346 | 1840508 | 3815641 | 5541541 | 0.304 | 0.689 | 0.441 (0.44-0.443) |
| Male | 721341 | 2116364 | 3715310 | 5224809 | 0.341 | 0.711 | 0.479 (0.478-0.481) |
| NH American Indian | 4734 | 14379 | 32037 | 46691 | 0.329 | 0.686 | 0.48 (0.463-0.497) |
| NH Asian Pacific | 30580 | 83477 | 161192 | 214248 | 0.366 | 0.752 | 0.487 (0.48-0.494) |
| NH Black | 125477 | 353362 | 758976 | 1014043 | 0.355 | 0.748 | 0.474 (0.471-0.478) |
| NH White | 1053419 | 3321921 | 6162078 | 8922171 | 0.317 | 0.691 | 0.459 (0.458-0.46) |
| Hispanic | 63840 | 176472 | 398775 | 544738 | 0.362 | 0.732 | 0.494 (0.489-0.499) |
| Northeast | 294639 | 801863 | 1481300 | 2038057 | 0.367 | 0.727 | 0.506 (0.503-0.508) |
| Midwest | 276055 | 941751 | 1730993 | 2531034 | 0.293 | 0.684 | 0.429 (0.427-0.431) |
| South | 422374 | 1426935 | 2855133 | 4165469 | 0.296 | 0.685 | 0.432 (0.43-0.433) |
| West | 287619 | 786323 | 1463525 | 2031790 | 0.366 | 0.720 | 0.508 (0.505-0.51) |
| Metro | 1034994 | 3206437 | 6028719 | 8615342 | 0.323 | 0.700 | 0.461 (0.46-0.462) |
| Non metro | 245693 | 750435 | 1502232 | 2151008 | 0.327 | 0.698 | 0.469 (0.466-0.471) |
| **85+ Age Group** | | | | | | | |
| Overall | 957197 | 2427137 | 10205477 | 14551863 | 0.394 | 0.701 | 0.562 (0.561-0.564) |
| Female | 491102 | 1289356 | 6691033 | 9594400 | 0.381 | 0.697 | 0.546 (0.544-0.548) |
| Male | 466095 | 1137781 | 3514444 | 4957463 | 0.410 | 0.709 | 0.578 (0.576-0.58) |
| NH American Indian | 2249 | 5956 | 24723 | 36716 | 0.378 | 0.673 | 0.561 (0.533-0.59) |
| NH Asian Pacific | 21021 | 49258 | 205412 | 279258 | 0.427 | 0.736 | 0.58 (0.57-0.59) |
| NH Black | 70780 | 178906 | 714720 | 990824 | 0.396 | 0.721 | 0.548 (0.543-0.554) |
| NH White | 821997 | 2093608 | 8811478 | 12625005 | 0.393 | 0.698 | 0.563 (0.561-0.564) |
| Hispanic | 39443 | 95469 | 430671 | 594386 | 0.413 | 0.725 | 0.57 (0.563-0.577) |
| Northeast | 232600 | 536664 | 2252255 | 3090418 | 0.433 | 0.729 | 0.595 (0.592-0.598) |
| Midwest | 222272 | 595580 | 2474128 | 3603835 | 0.373 | 0.687 | 0.544 (0.541-0.546) |
| South | 279007 | 788724 | 3420306 | 5019116 | 0.354 | 0.681 | 0.519 (0.517-0.521) |
| West | 223318 | 506169 | 2058788 | 2838494 | 0.441 | 0.725 | 0.608 (0.605-0.612) |
| Metro | 778819 | 1985535 | 8258722 | 11786492 | 0.392 | 0.701 | 0.56 (0.558-0.561) |
| Non metro | 178378 | 441602 | 1946755 | 2765371 | 0.404 | 0.704 | 0.574 (0.57-0.577) |

| **Supplementary Table 2: Average annual percent change (AAPC) in the Proportional Mortality Ratio for cardiovascular co-listing on malignant neoplasm death certificates, by age group and demographic subgroup in the United States, 1999–2020** | | | | | |
| --- | --- | --- | --- | --- | --- |
| **Proportional Mortality Ratio AAPC (1999-2020)** | | | | | |
| **Age Group** | **AAPC (95% CI)** | **p-value** | **Age Group** | **AAPC (95% CI)** | **p-value** |
| **Overall** | | | | | |
| 15-24 | 0.43 (-0.19-1.07) | 0.145171 | 55-64 | 1.69* (1.52-1.83) | < 0.000001 |
| 25-34 | 1.19* (0.79-1.63) | < 0.000001 | 65-74 | 1.38* (1.27-1.49) | < 0.000001 |
| 35-44 | 1.34* (1.13-1.57) | < 0.000001 | 75-84 | 1.07* (0.94-1.17) | < 0.000001 |
| 45-54 | 1.47* (1.34-1.61) | < 0.000001 | 85+ | 0.66* (0.52-0.76) | < 0.000001 |
| **CVD Burden in Malignant Cancer Deaths (A/B)** | | | | | |
| 15-24 | 0.25 (-0.14-0.67) | 0.207958 | 55-64 | 0.96* (0.85-1.07) | < 0.000001 |
| 25-34 | 0.89* (0.47-1.22) | < 0.000001 | 65-74 | 0.67* (0.6-0.73) | < 0.000001 |
| 35-44 | 1.15* (0.98-1.32) | < 0.000001 | 75-84 | 0.32* (0.24-0.38) | < 0.000001 |
| 45-54 | 1.15* (1-1.3) | < 0.000001 | 85+ | -0.04 (-0.11-0.02) | 0.205959 |
| **CVD Burden in Non-Malignant Cancer Deaths (C/D)** | | | | | |
| 15-24 | -0.23 (-0.52-0.07) | 0.095981 | 55-64 | -0.67* (-0.72--0.63) | < 0.000001 |
| 25-34 | -0.25* (-0.48--0.04) | 0.018796 | 65-74 | -0.67* (-0.71--0.6) | < 0.000001 |
| 35-44 | -0.37* (-0.48--0.23) | < 0.000001 | 75-84 | -0.77* (-0.82--0.7) | < 0.000001 |
| 45-54 | -0.40* (-0.46--0.31) | < 0.000001 | 85+ | -0.70* (-0.74--0.64) | < 0.000001 |
| **Female** | | | | | |
| 15-24 | 0.2 (-0.41-0.81) | 0.515097 | 55-64 | 1.69* (1.5-1.85) | < 0.000001 |
| 25-34 | 1.16* (0.74-1.66) | < 0.000001 | 65-74 | 1.31* (1.13-1.44) | < 0.000001 |
| 35-44 | 1.14* (0.85-1.43) | < 0.000001 | 75-84 | 1.05* (0.87-1.18) | < 0.000001 |
| 45-54 | 1.55* (1.35-1.73) | < 0.000001 | 85+ | 0.57* (0.39-0.69) | < 0.000001 |
| **Male** | | | | | |
| 15-24 | 0.6 (-0.03-1.22) | 0.057988 | 55-64 | 1.61* (1.48-1.75) | < 0.000001 |
| 25-34 | 1.27* (0.93-1.64) | < 0.000001 | 65-74 | 1.37* (1.22-1.52) | < 0.000001 |
| 35-44 | 1.60* (1.36-1.87) | < 0.000001 | 75-84 | 1.07* (0.95-1.17) | < 0.000001 |
| 45-54 | 1.44* (1.24-1.64) | < 0.000001 | 85+ | 0.62* (0.5-0.72) | < 0.000001 |
| **NH American Indian or Alaskan Native** | | | | | |
| 15-24 | - | - | 55-64 | 2.06* (1.37-2.72) | < 0.000001 |
| 25-34 | - | - | 65-74 | 2.20* (1.75-2.69) | < 0.000001 |
| 35-44 | - | - | 75-84 | 2.06* (1.27-2.75) | < 0.000001 |
| 45-54 | 0.41 (-0.21-1.71) | 0.221556 | 85+ | 1.14* (0.38-2.23) | 0.008798 |
| **NH Asian or Pacific Islander** | | | | | |
| 15-24 | -2.76* (-4.26--0.92) | 0.003199 | 55-64 | 0.76* (0.06-1.23) | 0.040392 |
| 25-34 | -0.52 (-1.51-0.57) | 0.341532 | 65-74 | 0.4 (-0.12-0.7) | 0.084383 |
| 35-44 | -0.33 (-1.12-0.37) | 0.405119 | 75-84 | 0.38* (0.1-0.81) | 0.013197 |
| 45-54 | 0.02 (-0.31-0.37) | 0.962208 | 85+ | -0.31* (-0.49--0.14) | 0.0004 |
| **NH Black or African American** | | | | | |
| 15-24 | 1.12* (0.01-2.37) | 0.04799 | 55-64 | 1.20* (1.01-1.38) | < 0.000001 |
| 25-34 | 0.41 (-0.6-1.51) | 0.395121 | 65-74 | 1.12* (0.91-1.38) | < 0.000001 |
| 35-44 | 0.67* (0.23-1.06) | 0.004399 | 75-84 | 0.89* (0.68-1.12) | < 0.000001 |
| 45-54 | 1.03* (0.67-1.31) | < 0.000001 | 85+ | 0.67* (0.46-0.86) | < 0.000001 |
| **NH White** | | | | | |
| 15-24 | -0.27 (-1.01-0.41) | 0.369126 | 55-64 | 1.64* (1.5-1.78) | < 0.000001 |
| 25-34 | 1.53* (1.06-2.01) | < 0.000001 | 65-74 | 1.36* (1.23-1.5) | < 0.000001 |
| 35-44 | 1.77* (1.48-2.07) | < 0.000001 | 75-84 | 1.13* (0.98-1.23) | < 0.000001 |
| 45-54 | 1.66* (1.54-1.79) | < 0.000001 | 85+ | 0.69* (0.55-0.79) | < 0.000001 |
| **Hispanic or Latino** | | | | | |
| 15-24 | -0.63 (-1.37-0.2) | 0.139972 | 55-64 | 1.46* (1.17-1.72) | < 0.000001 |
| 25-34 | -0.52 (-1.41-0.36) | 0.264747 | 65-74 | 1.31* (0.97-1.6) | < 0.000001 |
| 35-44 | -0.25 (-0.87-0.32) | 0.418716 | 75-84 | 1.04* (0.81-1.23) | < 0.000001 |
| 45-54 | 0.42* (0.06-0.79) | 0.030794 | 85+ | 0.53* (0.31-0.72) | < 0.000001 |
| **Northeast region** | | | | | |
| 15-24 | -1.36* (-2.52--0.4) | 0.009198 | 55-64 | 0.64* (0.36-0.81) | < 0.000001 |
| 25-34 | 0.29 (-0.8-1.22) | 0.561488 | 65-74 | 0.68* (0.49-0.86) | < 0.000001 |
| 35-44 | 0.3 (-0.27-0.79) | 0.241952 | 75-84 | 0.69* (0.46-0.88) | < 0.000001 |
| 45-54 | 0.59* (0.2-0.96) | 0.025195 | 85+ | 0.34* (0.06-0.49) | 0.025195 |
| **Midwest region** | | | | | |
| 15-24 | -2.52* (-3.73--1.1) | < 0.000001 | 55-64 | 1.90* (1.7-2.13) | < 0.000001 |
| 25-34 | 2.07* (1.17-3.35) | < 0.000001 | 65-74 | 1.48* (1.37-1.6) | < 0.000001 |
| 35-44 | 2.59* (1.96-3.27) | < 0.000001 | 75-84 | 1.18* (0.97-1.42) | < 0.000001 |
| 45-54 | 2.20* (1.88-2.53) | < 0.000001 | 85+ | 0.68* (0.54-0.85) | < 0.000001 |
| **South region** | | | | | |
| 15-24 | 0.73* (0.13-1.4) | 0.021196 | 55-64 | 2.02* (1.87-2.18) | < 0.000001 |
| 25-34 | 1.16* (0.51-1.78) | 0.0024 | 65-74 | 1.72* (1.55-1.91) | < 0.000001 |
| 35-44 | 1.52* (1.19-1.88) | < 0.000001 | 75-84 | 1.38* (1.27-1.47) | < 0.000001 |
| 45-54 | 1.85* (1.67-2.06) | < 0.000001 | 85+ | 0.88* (0.73-0.99) | < 0.000001 |
| **West region** | | | | | |
| 15-24 | -0.24 (-1.61-0.59) | 0.512697 | 55-64 | 1.92* (1.62-2.13) | < 0.000001 |
| 25-34 | 0.96* (0.1-1.96) | 0.028794 | 65-74 | 1.46* (1.3-1.58) | < 0.000001 |
| 35-44 | 1.40* (0.88-1.92) | < 0.000001 | 75-84 | 1.15* (1.02-1.25) | < 0.000001 |
| 45-54 | 1.56* (1.33-1.82) | < 0.000001 | 85+ | 0.74* (0.58-0.85) | < 0.000001 |
| **Metropolitan area** | | | | | |
| 15-24 | 0.36 (-0.62-1.27) | 0.40152 | 55-64 | 1.64* (1.47-1.78) | < 0.000001 |
| 25-34 | 1.13* (0.6-1.64) | < 0.000001 | 65-74 | 1.32* (1.19-1.44) | < 0.000001 |
| 35-44 | 1.29* (1.03-1.55) | < 0.000001 | 75-84 | 1.04* (0.91-1.14) | < 0.000001 |
| 45-54 | 1.34* (1.16-1.52) | < 0.000001 | 85+ | 0.60* (0.43-0.73) | < 0.000001 |
| **Nonmetropolitan areas** | | | | | |
| 15-24 | 0.69 (-0.3-1.75) | 0.169166 | 55-64 | 1.94* (1.79-2.11) | < 0.000001 |
| 25-34 | 1.88* (0.82-2.9) | 0.0004 | 65-74 | 1.51* (1.34-1.67) | < 0.000001 |
| 35-44 | 1.67* (1.28-2.08) | < 0.000001 | 75-84 | 1.35* (1.21-1.49) | < 0.000001 |
| 45-54 | 2.02* (1.69-2.31) | < 0.000001 | 85+ | 0.91* (0.79-1.05) | < 0.000001 |
| AAPC = average annual percent change; N.H. = non-Hispanic; * Indicates that the average annual percentage change (AAPC) is significantly different from zero at α = 0.05. | | | | | |

| **Supplemental Table 3:** **Annual CVD co-listing proportions in malignant neoplasm death certificates (A/B) and non-cancer death certificates [(C−A)/(D−B)], by age group in the United States, 1999–2020** | | | |
| --- | --- | --- | --- |
| **Year** | **CVD Burden in Malignant Cancer Deaths (A/B)** | **CVD Burden in Non-Malignant Cancer Deaths (C/D)** | **Proportional Mortality Ratio (95% CI)** |
| **15-24 Age Group** | | | |
| **1999** | 0.186 | 0.101 | 1.842 (1.629-2.082) |
| **2000** | 0.181 | 0.097 | 1.874 (1.654-2.122) |
| **2001** | 0.180 | 0.097 | 1.863 (1.644-2.111) |
| **2002** | 0.177 | 0.096 | 1.856 (1.638-2.104) |
| **2003** | 0.164 | 0.098 | 1.673 (1.468-1.907) |
| **2004** | 0.188 | 0.097 | 1.947 (1.723-2.201) |
| **2005** | 0.182 | 0.098 | 1.848 (1.632-2.092) |
| **2006** | 0.172 | 0.096 | 1.796 (1.579-2.043) |
| **2007** | 0.191 | 0.097 | 1.967 (1.738-2.226) |
| **2008** | 0.169 | 0.101 | 1.681 (1.477-1.914) |
| **2009** | 0.179 | 0.108 | 1.66 (1.461-1.886) |
| **2010** | 0.182 | 0.108 | 1.685 (1.483-1.915) |
| **2011** | 0.180 | 0.103 | 1.745 (1.534-1.985) |
| **2012** | 0.168 | 0.101 | 1.656 (1.449-1.892) |
| **2013** | 0.173 | 0.105 | 1.642 (1.435-1.879) |
| **2014** | 0.181 | 0.103 | 1.746 (1.533-1.988) |
| **2015** | 0.190 | 0.099 | 1.917 (1.682-2.186) |
| **2016** | 0.179 | 0.094 | 1.9 (1.659-2.175) |
| **2017** | 0.203 | 0.092 | 2.205 (1.934-2.515) |
| **2018** | 0.181 | 0.099 | 1.837 (1.602-2.106) |
| **2019** | 0.197 | 0.099 | 1.99 (1.745-2.271) |
| **2020** | 0.185 | 0.095 | 1.952 (1.7-2.241) |
| **25-34 Age Group** | | | |
| **1999** | 0.162 | 0.200 | 0.806 (0.742-0.875) |
| **2000** | 0.162 | 0.188 | 0.864 (0.793-0.942) |
| **2001** | 0.161 | 0.186 | 0.865 (0.794-0.942) |
| **2002** | 0.143 | 0.195 | 0.736 (0.672-0.806) |
| **2003** | 0.163 | 0.198 | 0.822 (0.753-0.897) |
| **2004** | 0.161 | 0.200 | 0.805 (0.736-0.88) |
| **2005** | 0.158 | 0.197 | 0.806 (0.736-0.882) |
| **2006** | 0.160 | 0.197 | 0.814 (0.744-0.89) |
| **2007** | 0.171 | 0.193 | 0.886 (0.81-0.968) |
| **2008** | 0.163 | 0.199 | 0.817 (0.747-0.894) |
| **2009** | 0.154 | 0.202 | 0.763 (0.697-0.835) |
| **2010** | 0.158 | 0.204 | 0.771 (0.704-0.843) |
| **2011** | 0.163 | 0.200 | 0.811 (0.741-0.887) |
| **2012** | 0.167 | 0.198 | 0.844 (0.774-0.921) |
| **2013** | 0.156 | 0.199 | 0.786 (0.719-0.859) |
| **2014** | 0.165 | 0.199 | 0.83 (0.76-0.906) |
| **2015** | 0.172 | 0.189 | 0.91 (0.835-0.991) |
| **2016** | 0.158 | 0.176 | 0.897 (0.822-0.979) |
| **2017** | 0.172 | 0.178 | 0.966 (0.887-1.052) |
| **2018** | 0.188 | 0.182 | 1.03 (0.95-1.118) |
| **2019** | 0.180 | 0.183 | 0.989 (0.909-1.075) |
| **2020** | 0.188 | 0.182 | 1.033 (0.952-1.121) |
| **35-44 Age Group** | | | |
| **1999** | 0.154 | 0.378 | 0.408 (0.391-0.426) |
| **2000** | 0.157 | 0.354 | 0.443 (0.424-0.462) |
| **2001** | 0.150 | 0.346 | 0.434 (0.415-0.453) |
| **2002** | 0.147 | 0.357 | 0.412 (0.394-0.431) |
| **2003** | 0.153 | 0.362 | 0.423 (0.405-0.443) |
| **2004** | 0.151 | 0.374 | 0.404 (0.385-0.423) |
| **2005** | 0.157 | 0.370 | 0.423 (0.404-0.443) |
| **2006** | 0.157 | 0.371 | 0.423 (0.404-0.444) |
| **2007** | 0.157 | 0.371 | 0.423 (0.404-0.444) |
| **2008** | 0.158 | 0.373 | 0.425 (0.404-0.446) |
| **2009** | 0.157 | 0.379 | 0.415 (0.396-0.436) |
| **2010** | 0.156 | 0.383 | 0.407 (0.387-0.428) |
| **2011** | 0.161 | 0.381 | 0.421 (0.401-0.443) |
| **2012** | 0.153 | 0.382 | 0.4 (0.379-0.421) |
| **2013** | 0.165 | 0.384 | 0.429 (0.407-0.451) |
| **2014** | 0.171 | 0.384 | 0.445 (0.424-0.468) |
| **2015** | 0.162 | 0.369 | 0.439 (0.417-0.462) |
| **2016** | 0.175 | 0.356 | 0.491 (0.467-0.516) |
| **2017** | 0.180 | 0.349 | 0.517 (0.492-0.543) |
| **2018** | 0.182 | 0.353 | 0.516 (0.491-0.542) |
| **2019** | 0.190 | 0.348 | 0.546 (0.52-0.573) |
| **2020** | 0.194 | 0.346 | 0.56 (0.535-0.587) |
| **45-54 Age Group** | | | |
| **1999** | 0.175 | 0.574 | 0.304 (0.297-0.312) |
| **2000** | 0.169 | 0.556 | 0.303 (0.296-0.311) |
| **2001** | 0.172 | 0.543 | 0.316 (0.309-0.324) |
| **2002** | 0.168 | 0.544 | 0.309 (0.301-0.317) |
| **2003** | 0.170 | 0.539 | 0.316 (0.308-0.323) |
| **2004** | 0.172 | 0.539 | 0.319 (0.311-0.327) |
| **2005** | 0.169 | 0.531 | 0.318 (0.31-0.326) |
| **2006** | 0.171 | 0.531 | 0.323 (0.315-0.331) |
| **2007** | 0.171 | 0.530 | 0.323 (0.315-0.331) |
| **2008** | 0.174 | 0.525 | 0.331 (0.323-0.339) |
| **2009** | 0.177 | 0.526 | 0.336 (0.328-0.344) |
| **2010** | 0.173 | 0.530 | 0.326 (0.319-0.334) |
| **2011** | 0.177 | 0.526 | 0.336 (0.328-0.344) |
| **2012** | 0.176 | 0.525 | 0.335 (0.327-0.343) |
| **2013** | 0.183 | 0.533 | 0.343 (0.335-0.352) |
| **2014** | 0.182 | 0.530 | 0.344 (0.335-0.352) |
| **2015** | 0.188 | 0.527 | 0.357 (0.349-0.367) |
| **2016** | 0.194 | 0.526 | 0.369 (0.36-0.378) |
| **2017** | 0.202 | 0.522 | 0.386 (0.377-0.396) |
| **2018** | 0.203 | 0.531 | 0.383 (0.374-0.394) |
| **2019** | 0.207 | 0.531 | 0.39 (0.38-0.4) |
| **2020** | 0.219 | 0.521 | 0.421 (0.41-0.432) |
| **55-64 Age Group** | | | |
| **1999** | 0.212 | 0.716 | 0.296 (0.291-0.301) |
| **2000** | 0.209 | 0.707 | 0.296 (0.291-0.301) |
| **2001** | 0.213 | 0.695 | 0.306 (0.301-0.311) |
| **2002** | 0.207 | 0.693 | 0.298 (0.293-0.303) |
| **2003** | 0.205 | 0.683 | 0.3 (0.295-0.306) |
| **2004** | 0.208 | 0.676 | 0.308 (0.302-0.313) |
| **2005** | 0.209 | 0.669 | 0.313 (0.308-0.318) |
| **2006** | 0.210 | 0.666 | 0.316 (0.311-0.321) |
| **2007** | 0.213 | 0.657 | 0.324 (0.319-0.329) |
| **2008** | 0.213 | 0.649 | 0.328 (0.323-0.333) |
| **2009** | 0.210 | 0.643 | 0.327 (0.322-0.332) |
| **2010** | 0.213 | 0.642 | 0.331 (0.326-0.337) |
| **2011** | 0.213 | 0.638 | 0.333 (0.328-0.338) |
| **2012** | 0.213 | 0.634 | 0.335 (0.33-0.34) |
| **2013** | 0.217 | 0.631 | 0.344 (0.339-0.349) |
| **2014** | 0.216 | 0.630 | 0.343 (0.338-0.348) |
| **2015** | 0.215 | 0.631 | 0.341 (0.336-0.346) |
| **2016** | 0.222 | 0.628 | 0.354 (0.349-0.359) |
| **2017** | 0.230 | 0.630 | 0.365 (0.36-0.37) |
| **2018** | 0.235 | 0.633 | 0.371 (0.366-0.376) |
| **2019** | 0.241 | 0.632 | 0.381 (0.375-0.386) |
| **2020** | 0.258 | 0.616 | 0.419 (0.413-0.425) |
| **65-74 Age Group** | | | |
| **1999** | 0.266 | 0.753 | 0.353 (0.348-0.357) |
| **2000** | 0.264 | 0.748 | 0.352 (0.348-0.357) |
| **2001** | 0.266 | 0.740 | 0.359 (0.355-0.363) |
| **2002** | 0.265 | 0.735 | 0.36 (0.356-0.365) |
| **2003** | 0.265 | 0.729 | 0.363 (0.358-0.367) |
| **2004** | 0.264 | 0.724 | 0.364 (0.36-0.369) |
| **2005** | 0.261 | 0.714 | 0.366 (0.361-0.37) |
| **2006** | 0.265 | 0.709 | 0.374 (0.369-0.379) |
| **2007** | 0.264 | 0.702 | 0.376 (0.372-0.381) |
| **2008** | 0.263 | 0.693 | 0.379 (0.375-0.384) |
| **2009** | 0.260 | 0.693 | 0.375 (0.371-0.38) |
| **2010** | 0.262 | 0.686 | 0.381 (0.376-0.386) |
| **2011** | 0.259 | 0.681 | 0.38 (0.376-0.385) |
| **2012** | 0.260 | 0.680 | 0.383 (0.379-0.388) |
| **2013** | 0.261 | 0.677 | 0.385 (0.38-0.39) |
| **2014** | 0.259 | 0.674 | 0.384 (0.38-0.389) |
| **2015** | 0.260 | 0.674 | 0.386 (0.381-0.39) |
| **2016** | 0.267 | 0.675 | 0.395 (0.39-0.399) |
| **2017** | 0.275 | 0.677 | 0.406 (0.401-0.41) |
| **2018** | 0.280 | 0.676 | 0.413 (0.409-0.418) |
| **2019** | 0.288 | 0.680 | 0.424 (0.419-0.428) |
| **2020** | 0.308 | 0.651 | 0.473 (0.468-0.478) |
| **75-84 Age Group** | | | |
| **1999** | 0.332 | 0.763 | 0.435 (0.43-0.439) |
| **2000** | 0.331 | 0.758 | 0.437 (0.433-0.442) |
| **2001** | 0.326 | 0.750 | 0.435 (0.43-0.439) |
| **2002** | 0.331 | 0.742 | 0.446 (0.441-0.45) |
| **2003** | 0.326 | 0.736 | 0.443 (0.439-0.448) |
| **2004** | 0.324 | 0.729 | 0.445 (0.44-0.449) |
| **2005** | 0.326 | 0.718 | 0.453 (0.449-0.458) |
| **2006** | 0.323 | 0.711 | 0.454 (0.45-0.459) |
| **2007** | 0.324 | 0.706 | 0.459 (0.454-0.463) |
| **2008** | 0.320 | 0.695 | 0.46 (0.455-0.465) |
| **2009** | 0.318 | 0.694 | 0.458 (0.453-0.463) |
| **2010** | 0.320 | 0.685 | 0.466 (0.461-0.471) |
| **2011** | 0.317 | 0.679 | 0.467 (0.462-0.472) |
| **2012** | 0.313 | 0.674 | 0.465 (0.46-0.47) |
| **2013** | 0.313 | 0.673 | 0.465 (0.46-0.47) |
| **2014** | 0.309 | 0.671 | 0.46 (0.456-0.465) |
| **2015** | 0.310 | 0.670 | 0.462 (0.457-0.467) |
| **2016** | 0.315 | 0.670 | 0.471 (0.466-0.476) |
| **2017** | 0.319 | 0.669 | 0.477 (0.472-0.482) |
| **2018** | 0.326 | 0.671 | 0.486 (0.481-0.491) |
| **2019** | 0.334 | 0.672 | 0.497 (0.492-0.502) |
| **2020** | 0.357 | 0.644 | 0.553 (0.548-0.559) |
| **85+ Age Group** | | | |
| **1999** | 0.419 | 0.766 | 0.546 (0.54-0.553) |
| **2000** | 0.415 | 0.762 | 0.544 (0.538-0.551) |
| **2001** | 0.414 | 0.753 | 0.549 (0.542-0.556) |
| **2002** | 0.411 | 0.747 | 0.551 (0.544-0.558) |
| **2003** | 0.409 | 0.739 | 0.553 (0.546-0.56) |
| **2004** | 0.405 | 0.734 | 0.552 (0.545-0.559) |
| **2005** | 0.403 | 0.724 | 0.557 (0.551-0.564) |
| **2006** | 0.402 | 0.717 | 0.56 (0.553-0.567) |
| **2007** | 0.400 | 0.712 | 0.561 (0.555-0.568) |
| **2008** | 0.392 | 0.702 | 0.558 (0.552-0.565) |
| **2009** | 0.390 | 0.699 | 0.558 (0.552-0.565) |
| **2010** | 0.388 | 0.692 | 0.561 (0.554-0.567) |
| **2011** | 0.386 | 0.682 | 0.567 (0.56-0.573) |
| **2012** | 0.385 | 0.678 | 0.568 (0.561-0.574) |
| **2013** | 0.379 | 0.676 | 0.561 (0.555-0.568) |
| **2014** | 0.373 | 0.676 | 0.552 (0.546-0.559) |
| **2015** | 0.372 | 0.676 | 0.55 (0.544-0.557) |
| **2016** | 0.374 | 0.677 | 0.553 (0.546-0.559) |
| **2017** | 0.381 | 0.678 | 0.562 (0.556-0.569) |
| **2018** | 0.386 | 0.679 | 0.569 (0.563-0.576) |
| **2019** | 0.394 | 0.682 | 0.577 (0.571-0.584) |
| **2020** | 0.420 | 0.657 | 0.638 (0.631-0.645) |

| **Supplemental Table 4** Proportional Mortality Ratio (95% CI) for cardiovascular co-listing on malignant neoplasm death certificates, stratified by sex and urbanisation level in the United States, 1999–2020 | | | | |
| --- | --- | --- | --- | --- |
| **Proportional Mortality Ratio (95% CI)** | | | | |
| **Year** | **Female** | **Male** | **Metropolitan Area** | **Non-Metropolitan Area** |
| **15-24 Age Group** | | | | |
| **1999** | 1.22 (1.005-1.482) | 2.245 (1.917-2.629) | 1.895 (1.659-2.165) | 1.567 (1.139-2.156) |
| **2000** | 1.34 (1.104-1.626) | 2.154 (1.829-2.536) | 2.053 (1.798-2.346) | 1.054 (0.725-1.532) |
| **2001** | 1.303 (1.067-1.591) | 2.171 (1.849-2.549) | 1.938 (1.691-2.22) | 1.533 (1.111-2.115) |
| **2002** | 1.337 (1.099-1.625) | 2.131 (1.811-2.509) | 1.985 (1.736-2.271) | 1.267 (0.895-1.793) |
| **2003** | 1.302 (1.064-1.593) | 1.83 (1.54-2.175) | 1.737 (1.508-2.002) | 1.357 (0.958-1.924) |
| **2004** | 1.417 (1.165-1.723) | 2.241 (1.915-2.621) | 2.036 (1.785-2.322) | 1.482 (1.057-2.077) |
| **2005** | 1.328 (1.087-1.622) | 2.128 (1.816-2.493) | 1.98 (1.731-2.263) | 1.287 (0.923-1.794) |
| **2006** | 1.221 (0.99-1.506) | 2.123 (1.802-2.5) | 1.962 (1.711-2.25) | 1.005 (0.676-1.495) |
| **2007** | 1.448 (1.192-1.759) | 2.218 (1.889-2.605) | 2.055 (1.802-2.345) | 1.448 (1.007-2.084) |
| **2008** | 1.152 (0.928-1.43) | 1.985 (1.688-2.335) | 1.725 (1.501-1.983) | 1.449 (1.015-2.069) |
| **2009** | 1.212 (0.987-1.489) | 1.907 (1.621-2.244) | 1.624 (1.413-1.868) | 1.842 (1.345-2.524) |
| **2010** | 1.253 (1.014-1.548) | 1.917 (1.633-2.252) | 1.698 (1.478-1.95) | 1.614 (1.157-2.25) |
| **2011** | 1.291 (1.05-1.588) | 1.987 (1.685-2.342) | 1.789 (1.56-2.053) | 1.449 (0.994-2.112) |
| **2012** | 1.186 (0.96-1.465) | 1.925 (1.62-2.287) | 1.766 (1.534-2.033) | 0.976 (0.63-1.512) |
| **2013** | 1.243 (1.007-1.534) | 1.855 (1.556-2.212) | 1.669 (1.444-1.929) | 1.49 (1.026-2.164) |
| **2014** | 1.356 (1.107-1.661) | 1.918 (1.619-2.273) | 1.79 (1.558-2.057) | 1.459 (1.006-2.117) |
| **2015** | 1.333 (1.076-1.651) | 2.277 (1.929-2.686) | 2.005 (1.742-2.307) | 1.482 (1.031-2.13) |
| **2016** | 1.265 (1.002-1.597) | 2.291 (1.94-2.706) | 1.987 (1.718-2.297) | 1.473 (1.01-2.149) |
| **2017** | 1.604 (1.297-1.984) | 2.546 (2.154-3.008) | 2.368 (2.062-2.719) | 1.295 (0.838-2.001) |
| **2018** | 1.381 (1.108-1.722) | 2.081 (1.748-2.478) | 1.864 (1.609-2.159) | 1.682 (1.156-2.449) |
| **2019** | 1.342 (1.088-1.655) | 2.406 (2.031-2.851) | 2.035 (1.764-2.347) | 1.801 (1.277-2.538) |
| **2020** | 1.212 (0.971-1.512) | 2.467 (2.067-2.944) | 2.037 (1.759-2.359) | 1.489 (0.99-2.238) |
| **25-34 Age Group** | | | | |
| **1999** | 0.572 (0.505-0.647) | 0.98 (0.876-1.097) | 0.823 (0.752-0.899) | 0.707 (0.565-0.885) |
| **2000** | 0.65 (0.575-0.735) | 1.005 (0.89-1.135) | 0.908 (0.828-0.995) | 0.615 (0.477-0.793) |
| **2001** | 0.635 (0.563-0.716) | 1.021 (0.904-1.154) | 0.896 (0.817-0.983) | 0.718 (0.575-0.897) |
| **2002** | 0.519 (0.453-0.594) | 0.91 (0.804-1.03) | 0.74 (0.67-0.817) | 0.718 (0.573-0.899) |
| **2003** | 0.628 (0.555-0.712) | 0.939 (0.83-1.063) | 0.862 (0.786-0.947) | 0.597 (0.462-0.772) |
| **2004** | 0.612 (0.539-0.696) | 0.931 (0.821-1.056) | 0.828 (0.752-0.912) | 0.689 (0.543-0.874) |
| **2005** | 0.596 (0.522-0.68) | 0.953 (0.841-1.078) | 0.838 (0.76-0.923) | 0.643 (0.504-0.82) |
| **2006** | 0.632 (0.556-0.718) | 0.923 (0.814-1.047) | 0.847 (0.77-0.932) | 0.634 (0.493-0.816) |
| **2007** | 0.712 (0.627-0.807) | 0.967 (0.851-1.098) | 0.911 (0.827-1.004) | 0.763 (0.606-0.961) |
| **2008** | 0.645 (0.566-0.735) | 0.914 (0.806-1.036) | 0.838 (0.76-0.923) | 0.718 (0.567-0.91) |
| **2009** | 0.654 (0.576-0.744) | 0.808 (0.711-0.919) | 0.8 (0.726-0.881) | 0.569 (0.441-0.736) |
| **2010** | 0.609 (0.534-0.694) | 0.878 (0.775-0.994) | 0.798 (0.724-0.879) | 0.63 (0.494-0.803) |
| **2011** | 0.671 (0.59-0.763) | 0.889 (0.784-1.009) | 0.832 (0.755-0.917) | 0.702 (0.552-0.894) |
| **2012** | 0.668 (0.588-0.759) | 0.966 (0.856-1.09) | 0.853 (0.776-0.938) | 0.804 (0.641-1.008) |
| **2013** | 0.649 (0.57-0.738) | 0.864 (0.764-0.977) | 0.791 (0.717-0.871) | 0.769 (0.615-0.961) |
| **2014** | 0.656 (0.577-0.745) | 0.951 (0.843-1.073) | 0.85 (0.773-0.935) | 0.736 (0.584-0.928) |
| **2015** | 0.705 (0.622-0.798) | 1.05 (0.933-1.182) | 0.93 (0.848-1.02) | 0.808 (0.642-1.018) |
| **2016** | 0.708 (0.625-0.801) | 1.015 (0.897-1.148) | 0.954 (0.869-1.047) | 0.626 (0.488-0.803) |
| **2017** | 0.787 (0.699-0.887) | 1.05 (0.927-1.189) | 0.985 (0.898-1.08) | 0.869 (0.69-1.094) |
| **2018** | 0.781 (0.696-0.877) | 1.202 (1.07-1.351) | 1.069 (0.979-1.166) | 0.827 (0.655-1.043) |
| **2019** | 0.7 (0.618-0.792) | 1.225 (1.092-1.374) | 1.023 (0.936-1.12) | 0.809 (0.638-1.026) |
| **2020** | 0.746 (0.662-0.839) | 1.258 (1.123-1.409) | 1.036 (0.948-1.132) | 1.029 (0.833-1.271) |
| **35-44 Age Group** | | | | |
| **1999** | 0.364 (0.343-0.387) | 0.441 (0.415-0.469) | 0.419 (0.401-0.439) | 0.352 (0.315-0.394) |
| **2000** | 0.405 (0.381-0.43) | 0.461 (0.433-0.491) | 0.456 (0.435-0.477) | 0.382 (0.342-0.426) |
| **2001** | 0.395 (0.371-0.419) | 0.458 (0.43-0.489) | 0.451 (0.43-0.472) | 0.355 (0.316-0.397) |
| **2002** | 0.376 (0.354-0.4) | 0.438 (0.41-0.468) | 0.426 (0.406-0.447) | 0.349 (0.312-0.391) |
| **2003** | 0.389 (0.366-0.414) | 0.45 (0.422-0.48) | 0.441 (0.42-0.463) | 0.341 (0.304-0.383) |
| **2004** | 0.378 (0.355-0.403) | 0.424 (0.396-0.453) | 0.415 (0.395-0.436) | 0.349 (0.31-0.393) |
| **2005** | 0.402 (0.378-0.429) | 0.437 (0.408-0.467) | 0.43 (0.409-0.452) | 0.39 (0.348-0.438) |
| **2006** | 0.402 (0.377-0.429) | 0.438 (0.408-0.469) | 0.434 (0.412-0.457) | 0.375 (0.333-0.421) |
| **2007** | 0.406 (0.38-0.433) | 0.434 (0.405-0.466) | 0.437 (0.416-0.461) | 0.353 (0.312-0.401) |
| **2008** | 0.397 (0.371-0.425) | 0.448 (0.417-0.481) | 0.429 (0.407-0.452) | 0.404 (0.357-0.456) |
| **2009** | 0.389 (0.364-0.417) | 0.44 (0.409-0.473) | 0.426 (0.404-0.449) | 0.359 (0.315-0.409) |
| **2010** | 0.387 (0.361-0.415) | 0.424 (0.393-0.457) | 0.416 (0.394-0.44) | 0.357 (0.312-0.407) |
| **2011** | 0.404 (0.377-0.432) | 0.438 (0.407-0.472) | 0.431 (0.409-0.456) | 0.37 (0.324-0.421) |
| **2012** | 0.371 (0.346-0.399) | 0.427 (0.395-0.461) | 0.407 (0.385-0.431) | 0.362 (0.316-0.414) |
| **2013** | 0.405 (0.378-0.434) | 0.452 (0.419-0.486) | 0.436 (0.413-0.461) | 0.388 (0.341-0.443) |
| **2014** | 0.416 (0.388-0.445) | 0.477 (0.443-0.513) | 0.454 (0.43-0.479) | 0.403 (0.354-0.459) |
| **2015** | 0.406 (0.378-0.436) | 0.468 (0.433-0.504) | 0.446 (0.422-0.471) | 0.406 (0.356-0.464) |
| **2016** | 0.447 (0.418-0.479) | 0.527 (0.49-0.568) | 0.504 (0.478-0.532) | 0.429 (0.376-0.489) |
| **2017** | 0.46 (0.429-0.492) | 0.571 (0.531-0.613) | 0.53 (0.502-0.559) | 0.46 (0.405-0.522) |
| **2018** | 0.474 (0.443-0.507) | 0.544 (0.505-0.585) | 0.523 (0.496-0.552) | 0.482 (0.426-0.546) |
| **2019** | 0.464 (0.434-0.496) | 0.627 (0.585-0.673) | 0.561 (0.533-0.591) | 0.469 (0.412-0.533) |
| **2020** | 0.498 (0.466-0.531) | 0.61 (0.569-0.654) | 0.569 (0.541-0.599) | 0.519 (0.459-0.587) |
| **45-54 Age Group** | | | | |
| **1999** | 0.295 (0.284-0.306) | 0.313 (0.302-0.324) | 0.309 (0.301-0.318) | 0.28 (0.263-0.298) |
| **2000** | 0.29 (0.279-0.301) | 0.315 (0.304-0.326) | 0.309 (0.301-0.318) | 0.276 (0.259-0.294) |
| **2001** | 0.302 (0.291-0.314) | 0.329 (0.318-0.34) | 0.326 (0.317-0.335) | 0.274 (0.258-0.291) |
| **2002** | 0.301 (0.29-0.312) | 0.317 (0.306-0.327) | 0.314 (0.306-0.323) | 0.285 (0.268-0.302) |
| **2003** | 0.31 (0.299-0.322) | 0.322 (0.311-0.333) | 0.32 (0.311-0.329) | 0.295 (0.278-0.313) |
| **2004** | 0.312 (0.3-0.323) | 0.327 (0.316-0.338) | 0.324 (0.315-0.332) | 0.297 (0.28-0.315) |
| **2005** | 0.303 (0.293-0.315) | 0.333 (0.322-0.344) | 0.32 (0.312-0.329) | 0.305 (0.288-0.324) |
| **2006** | 0.314 (0.303-0.325) | 0.333 (0.322-0.344) | 0.326 (0.317-0.334) | 0.309 (0.292-0.328) |
| **2007** | 0.318 (0.307-0.33) | 0.33 (0.319-0.341) | 0.329 (0.321-0.338) | 0.294 (0.277-0.312) |
| **2008** | 0.328 (0.316-0.34) | 0.337 (0.326-0.348) | 0.335 (0.326-0.344) | 0.312 (0.294-0.33) |
| **2009** | 0.338 (0.326-0.35) | 0.338 (0.327-0.349) | 0.34 (0.331-0.349) | 0.318 (0.3-0.337) |
| **2010** | 0.329 (0.317-0.341) | 0.327 (0.317-0.338) | 0.331 (0.322-0.34) | 0.306 (0.288-0.324) |
| **2011** | 0.34 (0.328-0.352) | 0.338 (0.327-0.35) | 0.34 (0.331-0.349) | 0.319 (0.302-0.338) |
| **2012** | 0.335 (0.323-0.348) | 0.339 (0.328-0.351) | 0.336 (0.327-0.345) | 0.33 (0.311-0.349) |
| **2013** | 0.345 (0.333-0.358) | 0.345 (0.334-0.357) | 0.348 (0.339-0.358) | 0.319 (0.301-0.338) |
| **2014** | 0.346 (0.333-0.359) | 0.346 (0.335-0.358) | 0.35 (0.34-0.359) | 0.316 (0.297-0.335) |
| **2015** | 0.355 (0.342-0.368) | 0.364 (0.352-0.377) | 0.364 (0.355-0.375) | 0.326 (0.307-0.347) |
| **2016** | 0.36 (0.347-0.373) | 0.384 (0.371-0.397) | 0.371 (0.361-0.381) | 0.36 (0.339-0.382) |
| **2017** | 0.377 (0.364-0.391) | 0.399 (0.385-0.413) | 0.391 (0.38-0.402) | 0.365 (0.344-0.389) |
| **2018** | 0.381 (0.367-0.395) | 0.391 (0.377-0.405) | 0.383 (0.373-0.395) | 0.384 (0.361-0.409) |
| **2019** | 0.377 (0.362-0.391) | 0.408 (0.393-0.423) | 0.39 (0.379-0.402) | 0.389 (0.365-0.414) |
| **2020** | 0.413 (0.398-0.428) | 0.432 (0.417-0.448) | 0.418 (0.407-0.43) | 0.432 (0.406-0.458) |
| **55-64 Age Group** | | | | |
| **1999** | 0.29 (0.282-0.297) | 0.302 (0.296-0.309) | 0.299 (0.294-0.305) | 0.281 (0.27-0.293) |
| **2000** | 0.286 (0.279-0.294) | 0.305 (0.298-0.312) | 0.3 (0.295-0.306) | 0.277 (0.266-0.288) |
| **2001** | 0.299 (0.291-0.307) | 0.313 (0.307-0.321) | 0.309 (0.304-0.315) | 0.291 (0.279-0.303) |
| **2002** | 0.292 (0.285-0.3) | 0.304 (0.297-0.311) | 0.298 (0.293-0.304) | 0.296 (0.285-0.308) |
| **2003** | 0.291 (0.283-0.298) | 0.31 (0.304-0.317) | 0.302 (0.296-0.307) | 0.295 (0.284-0.307) |
| **2004** | 0.301 (0.293-0.309) | 0.315 (0.308-0.322) | 0.31 (0.304-0.316) | 0.297 (0.285-0.308) |
| **2005** | 0.307 (0.299-0.315) | 0.321 (0.314-0.328) | 0.314 (0.309-0.32) | 0.308 (0.296-0.32) |
| **2006** | 0.309 (0.301-0.317) | 0.324 (0.317-0.331) | 0.316 (0.31-0.322) | 0.315 (0.304-0.327) |
| **2007** | 0.316 (0.308-0.324) | 0.333 (0.326-0.34) | 0.326 (0.32-0.331) | 0.318 (0.307-0.33) |
| **2008** | 0.32 (0.313-0.329) | 0.336 (0.329-0.343) | 0.329 (0.323-0.335) | 0.322 (0.31-0.334) |
| **2009** | 0.315 (0.308-0.323) | 0.338 (0.331-0.345) | 0.327 (0.321-0.333) | 0.327 (0.316-0.339) |
| **2010** | 0.324 (0.316-0.332) | 0.34 (0.333-0.346) | 0.334 (0.328-0.34) | 0.32 (0.309-0.332) |
| **2011** | 0.326 (0.319-0.334) | 0.341 (0.334-0.348) | 0.336 (0.33-0.342) | 0.321 (0.31-0.333) |
| **2012** | 0.327 (0.319-0.335) | 0.344 (0.337-0.351) | 0.338 (0.332-0.343) | 0.325 (0.313-0.336) |
| **2013** | 0.338 (0.33-0.346) | 0.351 (0.345-0.358) | 0.346 (0.341-0.352) | 0.334 (0.322-0.346) |
| **2014** | 0.337 (0.329-0.345) | 0.351 (0.344-0.358) | 0.345 (0.34-0.351) | 0.333 (0.322-0.345) |
| **2015** | 0.333 (0.325-0.341) | 0.35 (0.343-0.357) | 0.342 (0.336-0.347) | 0.339 (0.328-0.351) |
| **2016** | 0.344 (0.337-0.352) | 0.365 (0.358-0.372) | 0.355 (0.349-0.361) | 0.35 (0.338-0.362) |
| **2017** | 0.356 (0.348-0.364) | 0.375 (0.368-0.382) | 0.365 (0.36-0.371) | 0.363 (0.351-0.375) |
| **2018** | 0.357 (0.349-0.365) | 0.386 (0.378-0.393) | 0.371 (0.365-0.377) | 0.372 (0.36-0.385) |
| **2019** | 0.369 (0.361-0.378) | 0.394 (0.386-0.401) | 0.38 (0.374-0.386) | 0.385 (0.373-0.398) |
| **2020** | 0.411 (0.402-0.419) | 0.43 (0.422-0.438) | 0.419 (0.413-0.425) | 0.419 (0.406-0.432) |
| **65-74 Age Group** | | | | |
| **1999** | 0.336 (0.33-0.342) | 0.365 (0.36-0.371) | 0.353 (0.348-0.358) | 0.351 (0.341-0.361) |
| **2000** | 0.336 (0.33-0.343) | 0.365 (0.359-0.37) | 0.354 (0.349-0.358) | 0.346 (0.337-0.356) |
| **2001** | 0.347 (0.34-0.353) | 0.368 (0.362-0.374) | 0.359 (0.354-0.364) | 0.357 (0.347-0.367) |
| **2002** | 0.35 (0.343-0.356) | 0.368 (0.362-0.374) | 0.361 (0.356-0.366) | 0.356 (0.347-0.366) |
| **2003** | 0.351 (0.344-0.357) | 0.372 (0.366-0.378) | 0.362 (0.357-0.367) | 0.365 (0.355-0.375) |
| **2004** | 0.352 (0.345-0.359) | 0.375 (0.368-0.381) | 0.365 (0.36-0.37) | 0.362 (0.352-0.372) |
| **2005** | 0.35 (0.343-0.357) | 0.378 (0.371-0.384) | 0.365 (0.36-0.37) | 0.368 (0.358-0.379) |
| **2006** | 0.363 (0.356-0.37) | 0.383 (0.376-0.389) | 0.373 (0.367-0.378) | 0.379 (0.368-0.39) |
| **2007** | 0.363 (0.356-0.37) | 0.387 (0.381-0.394) | 0.377 (0.372-0.383) | 0.373 (0.362-0.383) |
| **2008** | 0.366 (0.359-0.373) | 0.39 (0.384-0.397) | 0.379 (0.373-0.384) | 0.382 (0.371-0.392) |
| **2009** | 0.361 (0.354-0.368) | 0.387 (0.381-0.394) | 0.375 (0.369-0.38) | 0.378 (0.368-0.389) |
| **2010** | 0.37 (0.363-0.377) | 0.391 (0.384-0.397) | 0.382 (0.377-0.387) | 0.378 (0.368-0.389) |
| **2011** | 0.368 (0.361-0.375) | 0.39 (0.384-0.397) | 0.381 (0.376-0.387) | 0.376 (0.366-0.387) |
| **2012** | 0.369 (0.362-0.376) | 0.395 (0.389-0.402) | 0.384 (0.379-0.389) | 0.38 (0.37-0.391) |
| **2013** | 0.372 (0.365-0.379) | 0.396 (0.39-0.402) | 0.385 (0.38-0.391) | 0.383 (0.373-0.393) |
| **2014** | 0.37 (0.363-0.376) | 0.396 (0.39-0.403) | 0.383 (0.378-0.388) | 0.39 (0.38-0.401) |
| **2015** | 0.372 (0.365-0.379) | 0.397 (0.391-0.403) | 0.384 (0.379-0.389) | 0.391 (0.381-0.401) |
| **2016** | 0.379 (0.372-0.386) | 0.408 (0.402-0.414) | 0.392 (0.387-0.397) | 0.407 (0.397-0.418) |
| **2017** | 0.388 (0.381-0.395) | 0.42 (0.414-0.426) | 0.405 (0.4-0.41) | 0.41 (0.4-0.421) |
| **2018** | 0.397 (0.39-0.403) | 0.427 (0.421-0.434) | 0.411 (0.406-0.416) | 0.424 (0.413-0.434) |
| **2019** | 0.401 (0.395-0.408) | 0.441 (0.435-0.448) | 0.42 (0.415-0.425) | 0.439 (0.428-0.449) |
| **2020** | 0.451 (0.444-0.458) | 0.491 (0.484-0.497) | 0.47 (0.464-0.475) | 0.486 (0.474-0.497) |
| **75-84 Age Group** | | | | |
| **1999** | 0.413 (0.407-0.419) | 0.453 (0.447-0.459) | 0.436 (0.432-0.441) | 0.427 (0.417-0.437) |
| **2000** | 0.416 (0.41-0.422) | 0.456 (0.449-0.462) | 0.437 (0.432-0.442) | 0.439 (0.429-0.449) |
| **2001** | 0.415 (0.409-0.421) | 0.451 (0.445-0.457) | 0.435 (0.43-0.44) | 0.432 (0.422-0.442) |
| **2002** | 0.427 (0.421-0.433) | 0.461 (0.455-0.468) | 0.444 (0.439-0.449) | 0.452 (0.442-0.463) |
| **2003** | 0.422 (0.415-0.428) | 0.461 (0.455-0.467) | 0.442 (0.437-0.447) | 0.447 (0.437-0.458) |
| **2004** | 0.422 (0.416-0.428) | 0.463 (0.457-0.47) | 0.442 (0.437-0.447) | 0.456 (0.446-0.467) |
| **2005** | 0.438 (0.431-0.444) | 0.466 (0.459-0.472) | 0.452 (0.447-0.458) | 0.457 (0.447-0.468) |
| **2006** | 0.435 (0.429-0.442) | 0.47 (0.463-0.476) | 0.452 (0.447-0.457) | 0.464 (0.454-0.475) |
| **2007** | 0.436 (0.429-0.443) | 0.477 (0.47-0.483) | 0.457 (0.451-0.462) | 0.467 (0.456-0.478) |
| **2008** | 0.441 (0.435-0.448) | 0.474 (0.468-0.481) | 0.458 (0.453-0.464) | 0.467 (0.457-0.479) |
| **2009** | 0.44 (0.433-0.447) | 0.472 (0.465-0.479) | 0.457 (0.452-0.463) | 0.462 (0.451-0.474) |
| **2010** | 0.446 (0.439-0.453) | 0.482 (0.475-0.489) | 0.466 (0.461-0.472) | 0.467 (0.456-0.479) |
| **2011** | 0.45 (0.443-0.457) | 0.479 (0.472-0.485) | 0.467 (0.462-0.473) | 0.465 (0.454-0.476) |
| **2012** | 0.448 (0.441-0.455) | 0.477 (0.47-0.484) | 0.464 (0.459-0.47) | 0.469 (0.458-0.481) |
| **2013** | 0.446 (0.439-0.454) | 0.479 (0.472-0.486) | 0.467 (0.462-0.473) | 0.458 (0.446-0.469) |
| **2014** | 0.438 (0.431-0.445) | 0.477 (0.47-0.484) | 0.459 (0.453-0.464) | 0.467 (0.456-0.479) |
| **2015** | 0.441 (0.434-0.448) | 0.478 (0.471-0.485) | 0.462 (0.457-0.468) | 0.463 (0.452-0.474) |
| **2016** | 0.45 (0.443-0.458) | 0.486 (0.479-0.493) | 0.468 (0.463-0.474) | 0.481 (0.47-0.493) |
| **2017** | 0.455 (0.447-0.462) | 0.494 (0.487-0.501) | 0.475 (0.47-0.481) | 0.487 (0.475-0.498) |
| **2018** | 0.465 (0.458-0.473) | 0.502 (0.495-0.509) | 0.482 (0.476-0.487) | 0.506 (0.495-0.518) |
| **2019** | 0.473 (0.465-0.48) | 0.515 (0.508-0.522) | 0.492 (0.486-0.498) | 0.52 (0.508-0.532) |
| **2020** | 0.525 (0.518-0.533) | 0.574 (0.567-0.582) | 0.548 (0.542-0.554) | 0.574 (0.562-0.587) |
| **85+ Age Group** | | | | |
| **1999** | 0.534 (0.525-0.543) | 0.565 (0.555-0.576) | 0.548 (0.54-0.556) | 0.54 (0.525-0.555) |
| **2000** | 0.533 (0.524-0.542) | 0.561 (0.551-0.572) | 0.545 (0.537-0.552) | 0.543 (0.528-0.558) |
| **2001** | 0.539 (0.53-0.547) | 0.565 (0.555-0.576) | 0.547 (0.539-0.554) | 0.559 (0.543-0.574) |
| **2002** | 0.537 (0.528-0.545) | 0.569 (0.559-0.58) | 0.551 (0.543-0.559) | 0.55 (0.535-0.565) |
| **2003** | 0.538 (0.529-0.547) | 0.572 (0.562-0.583) | 0.551 (0.543-0.559) | 0.562 (0.546-0.577) |
| **2004** | 0.539 (0.53-0.548) | 0.567 (0.556-0.578) | 0.547 (0.54-0.555) | 0.57 (0.555-0.586) |
| **2005** | 0.545 (0.536-0.554) | 0.57 (0.56-0.581) | 0.555 (0.548-0.563) | 0.566 (0.55-0.581) |
| **2006** | 0.546 (0.537-0.555) | 0.575 (0.564-0.585) | 0.558 (0.55-0.565) | 0.57 (0.554-0.585) |
| **2007** | 0.55 (0.541-0.559) | 0.572 (0.562-0.583) | 0.56 (0.553-0.568) | 0.566 (0.55-0.581) |
| **2008** | 0.544 (0.535-0.553) | 0.572 (0.562-0.582) | 0.555 (0.547-0.562) | 0.575 (0.56-0.591) |
| **2009** | 0.541 (0.532-0.55) | 0.575 (0.565-0.585) | 0.555 (0.548-0.562) | 0.575 (0.559-0.591) |
| **2010** | 0.547 (0.539-0.556) | 0.572 (0.562-0.582) | 0.559 (0.552-0.567) | 0.567 (0.552-0.582) |
| **2011** | 0.551 (0.542-0.56) | 0.58 (0.57-0.59) | 0.565 (0.558-0.572) | 0.574 (0.558-0.59) |
| **2012** | 0.55 (0.542-0.559) | 0.582 (0.572-0.592) | 0.568 (0.56-0.575) | 0.568 (0.553-0.584) |
| **2013** | 0.548 (0.539-0.557) | 0.571 (0.561-0.58) | 0.561 (0.554-0.568) | 0.566 (0.55-0.581) |
| **2014** | 0.534 (0.525-0.543) | 0.566 (0.557-0.576) | 0.55 (0.543-0.557) | 0.561 (0.546-0.577) |
| **2015** | 0.53 (0.522-0.539) | 0.567 (0.558-0.576) | 0.546 (0.539-0.553) | 0.57 (0.555-0.586) |
| **2016** | 0.534 (0.526-0.543) | 0.566 (0.557-0.576) | 0.548 (0.541-0.555) | 0.576 (0.561-0.592) |
| **2017** | 0.546 (0.537-0.554) | 0.574 (0.564-0.583) | 0.558 (0.551-0.565) | 0.583 (0.568-0.599) |
| **2018** | 0.549 (0.54-0.558) | 0.584 (0.575-0.593) | 0.563 (0.556-0.57) | 0.6 (0.584-0.616) |
| **2019** | 0.556 (0.547-0.564) | 0.593 (0.584-0.602) | 0.57 (0.563-0.577) | 0.615 (0.599-0.631) |
| **2020** | 0.616 (0.607-0.626) | 0.654 (0.644-0.663) | 0.634 (0.627-0.641) | 0.659 (0.643-0.676) |

| **Supplemental Table 5** Proportional Mortality Ratio (95% CI) for cardiovascular co-listing on malignant neoplasm death certificates, stratified by US Census region in the United States, 1999–2020 | | | | | |
| --- | --- | --- | --- | --- | --- |
| **Proportional Mortality Ratio (95% CI)** | | | | | |
| **Year** | **Northeast** | **Midwest** | **South** | **West** | |
| **15-24 Age Group** | | | | | |
| **1999** | 2.641 (2.039-3.421) | 2.422 (1.789-3.279) | 1.25 (0.993-1.573) | 2.584 (2.057-3.246) | |
| **2000** | 2.928 (2.271-3.775) | 0.983 (0.695-1.39) | 1.537 (1.235-1.912) | 2.498 (1.963-3.18) | |
| **2001** | 2.629 (2.014-3.432) | 1.575 (1.206-2.059) | 1.352 (1.07-1.708) | 2.515 (1.965-3.22) | |
| **2002** | 2.426 (1.864-3.158) | 1.341 (0.977-1.84) | 1.623 (1.31-2.012) | 2.184 (1.713-2.786) | |
| **2003** | 2.354 (1.782-3.109) | 0.89 (0.618-1.281) | 1.541 (1.234-1.924) | 2.112 (1.649-2.704) | |
| **2004** | 2.835 (2.21-3.638) | 1.104 (0.798-1.528) | 1.803 (1.466-2.218) | 2.291 (1.79-2.933) | |
| **2005** | 2.359 (1.82-3.057) | 0.978 (0.682-1.402) | 1.653 (1.333-2.049) | 2.443 (1.945-3.068) | |
| **2006** | 1.775 (1.315-2.395) | 1.284 (0.926-1.78) | 1.681 (1.347-2.099) | 2.428 (1.925-3.062) | |
| **2007** | 2.788 (2.154-3.608) | 1.393 (1.021-1.902) | 1.547 (1.23-1.946) | 2.372 (1.89-2.977) | |
| **2008** | 2.101 (1.595-2.768) | 1.061 (0.735-1.533) | 1.487 (1.192-1.856) | 2.02 (1.59-2.565) | |
| **2009** | 2.077 (1.576-2.739) | 1.218 (0.887-1.672) | 1.58 (1.276-1.957) | 1.84 (1.431-2.365) | |
| **2010** | 1.96 (1.471-2.611) | 1.378 (1.005-1.89) | 1.577 (1.273-1.954) | 1.861 (1.458-2.376) | |
| **2011** | 1.691 (1.242-2.301) | 1.231 (0.884-1.715) | 1.655 (1.333-2.055) | 2.31 (1.832-2.914) | |
| **2012** | 1.935 (1.41-2.655) | 1.461 (1.051-2.031) | 1.362 (1.08-1.719) | 1.98 (1.561-2.51) | |
| **2013** | 1.87 (1.366-2.559) | 1.175 (0.827-1.669) | 1.594 (1.275-1.993) | 1.889 (1.474-2.422) | |
| **2014** | 1.916 (1.416-2.592) | 1.402 (1.001-1.964) | 1.752 (1.423-2.157) | 1.826 (1.422-2.346) | |
| **2015** | 2.248 (1.662-3.041) | 1.311 (0.93-1.846) | 1.598 (1.269-2.012) | 2.578 (2.048-3.246) | |
| **2016** | 1.691 (1.189-2.406) | 1.158 (0.784-1.712) | 1.784 (1.437-2.214) | 2.753 (2.168-3.497) | |
| **2017** | 2.773 (2.023-3.802) | 1.284 (0.873-1.889) | 1.805 (1.455-2.239) | 3.252 (2.575-4.106) | |
| **2018** | 2.013 (1.466-2.763) | 1.142 (0.785-1.66) | 1.691 (1.347-2.123) | 2.463 (1.921-3.156) | |
| **2019** | 2.187 (1.619-2.955) | 1.408 (0.997-1.987) | 1.759 (1.41-2.195) | 2.644 (2.073-3.373) | |
| **2020** | 2.107 (1.542-2.878) | 1.351 (0.918-1.989) | 1.74 (1.388-2.181) | 2.521 (1.951-3.259) | |
| **25-34 Age Group** | | | | | |
| **1999** | 1.145 (0.976-1.342) | 0.509 (0.409-0.633) | 0.688 (0.596-0.795) | 0.958 (0.805-1.139) | |
| **2000** | 1.382 (1.172-1.629) | 0.561 (0.454-0.694) | 0.644 (0.55-0.754) | 1.117 (0.936-1.333) | |
| **2001** | 1.369 (1.153-1.626) | 0.46 (0.365-0.58) | 0.748 (0.648-0.863) | 1.145 (0.963-1.362) | |
| **2002** | 0.961 (0.791-1.167) | 0.499 (0.398-0.624) | 0.679 (0.584-0.789) | 0.911 (0.757-1.096) | |
| **2003** | 1.484 (1.256-1.753) | 0.424 (0.331-0.544) | 0.675 (0.58-0.786) | 0.935 (0.78-1.12) | |
| **2004** | 1.156 (0.962-1.39) | 0.486 (0.381-0.619) | 0.682 (0.586-0.793) | 1.035 (0.868-1.234) | |
| **2005** | 1.168 (0.968-1.408) | 0.451 (0.353-0.577) | 0.723 (0.622-0.84) | 1.017 (0.85-1.217) | |
| **2006** | 1.278 (1.056-1.546) | 0.473 (0.372-0.603) | 0.713 (0.614-0.828) | 0.997 (0.839-1.184) | |
| **2007** | 1.168 (0.96-1.421) | 0.522 (0.411-0.663) | 0.823 (0.711-0.953) | 1.128 (0.949-1.339) | |
| **2008** | 1.169 (0.967-1.413) | 0.462 (0.36-0.593) | 0.708 (0.608-0.825) | 1.06 (0.892-1.26) | |
| **2009** | 1.046 (0.861-1.272) | 0.447 (0.348-0.575) | 0.747 (0.646-0.864) | 0.852 (0.713-1.018) | |
| **2010** | 1.127 (0.921-1.379) | 0.546 (0.434-0.686) | 0.613 (0.526-0.715) | 1.028 (0.869-1.215) | |
| **2011** | 1.183 (0.975-1.434) | 0.561 (0.445-0.706) | 0.709 (0.61-0.824) | 0.945 (0.792-1.127) | |
| **2012** | 1.179 (0.97-1.434) | 0.542 (0.428-0.686) | 0.73 (0.632-0.843) | 1.084 (0.919-1.279) | |
| **2013** | 0.997 (0.805-1.234) | 0.702 (0.564-0.873) | 0.673 (0.582-0.778) | 0.914 (0.773-1.082) | |
| **2014** | 1.089 (0.883-1.343) | 0.583 (0.463-0.733) | 0.698 (0.603-0.808) | 1.101 (0.94-1.29) | |
| **2015** | 1.167 (0.948-1.436) | 0.626 (0.502-0.781) | 0.84 (0.733-0.964) | 1.111 (0.947-1.304) | |
| **2016** | 1.23 (0.999-1.513) | 0.627 (0.497-0.791) | 0.787 (0.684-0.906) | 1.088 (0.925-1.28) | |
| **2017** | 1.32 (1.067-1.633) | 0.827 (0.668-1.024) | 0.761 (0.661-0.876) | 1.219 (1.043-1.426) | |
| **2018** | 1.47 (1.212-1.783) | 0.678 (0.54-0.851) | 0.852 (0.745-0.974) | 1.344 (1.158-1.56) | |
| **2019** | 1.337 (1.087-1.645) | 0.786 (0.638-0.968) | 0.873 (0.762-1.001) | 1.141 (0.976-1.334) | |
| **2020** | 1.268 (1.044-1.541) | 0.828 (0.674-1.017) | 0.886 (0.772-1.017) | 1.26 (1.087-1.46) | |
| **35-44 Age Group** | | | | | |
| **1999** | 0.577 (0.531-0.628) | 0.268 (0.241-0.298) | 0.35 (0.326-0.376) | 0.517 (0.472-0.566) | |
| **2000** | 0.684 (0.629-0.745) | 0.283 (0.254-0.315) | 0.378 (0.351-0.407) | 0.537 (0.489-0.589) | |
| **2001** | 0.65 (0.595-0.71) | 0.283 (0.253-0.315) | 0.353 (0.327-0.38) | 0.597 (0.545-0.655) | |
| **2002** | 0.645 (0.59-0.704) | 0.262 (0.235-0.293) | 0.352 (0.326-0.379) | 0.498 (0.453-0.549) | |
| **2003** | 0.648 (0.593-0.709) | 0.264 (0.236-0.296) | 0.36 (0.333-0.388) | 0.536 (0.488-0.589) | |
| **2004** | 0.578 (0.527-0.634) | 0.252 (0.224-0.284) | 0.351 (0.325-0.379) | 0.519 (0.471-0.572) | |
| **2005** | 0.617 (0.563-0.677) | 0.269 (0.239-0.302) | 0.373 (0.346-0.403) | 0.518 (0.47-0.572) | |
| **2006** | 0.589 (0.535-0.649) | 0.263 (0.233-0.297) | 0.386 (0.358-0.416) | 0.526 (0.478-0.58) | |
| **2007** | 0.606 (0.549-0.668) | 0.266 (0.235-0.302) | 0.362 (0.334-0.392) | 0.551 (0.499-0.607) | |
| **2008** | 0.558 (0.503-0.619) | 0.292 (0.259-0.33) | 0.366 (0.337-0.396) | 0.576 (0.521-0.636) | |
| **2009** | 0.559 (0.504-0.621) | 0.247 (0.217-0.282) | 0.357 (0.329-0.388) | 0.579 (0.525-0.637) | |
| **2010** | 0.554 (0.497-0.619) | 0.254 (0.222-0.29) | 0.358 (0.33-0.389) | 0.544 (0.492-0.602) | |
| **2011** | 0.55 (0.492-0.615) | 0.302 (0.267-0.342) | 0.352 (0.323-0.382) | 0.58 (0.526-0.641) | |
| **2012** | 0.534 (0.476-0.599) | 0.259 (0.226-0.297) | 0.342 (0.314-0.372) | 0.556 (0.503-0.616) | |
| **2013** | 0.533 (0.476-0.597) | 0.306 (0.269-0.347) | 0.363 (0.334-0.395) | 0.596 (0.541-0.656) | |
| **2014** | 0.57 (0.507-0.64) | 0.342 (0.302-0.387) | 0.373 (0.344-0.405) | 0.611 (0.555-0.674) | |
| **2015** | 0.607 (0.538-0.684) | 0.273 (0.238-0.313) | 0.394 (0.363-0.428) | 0.589 (0.533-0.651) | |
| **2016** | 0.614 (0.545-0.692) | 0.321 (0.281-0.367) | 0.438 (0.404-0.474) | 0.666 (0.605-0.732) | |
| **2017** | 0.678 (0.601-0.764) | 0.393 (0.347-0.444) | 0.456 (0.422-0.493) | 0.655 (0.594-0.721) | |
| **2018** | 0.683 (0.604-0.771) | 0.389 (0.343-0.44) | 0.459 (0.424-0.496) | 0.64 (0.582-0.704) | |
| **2019** | 0.652 (0.577-0.737) | 0.436 (0.387-0.49) | 0.495 (0.459-0.535) | 0.677 (0.616-0.743) | |
| **2020** | 0.678 (0.604-0.761) | 0.471 (0.42-0.529) | 0.475 (0.44-0.513) | 0.722 (0.66-0.789) | |
| **45-54 Age Group** | | | | | |
| **1999** | 0.425 (0.404-0.447) | 0.222 (0.209-0.235) | 0.259 (0.248-0.27) | 0.367 (0.348-0.388) | |
| **2000** | 0.427 (0.406-0.45) | 0.212 (0.2-0.225) | 0.262 (0.251-0.273) | 0.378 (0.357-0.399) | |
| **2001** | 0.462 (0.44-0.486) | 0.216 (0.204-0.229) | 0.266 (0.255-0.277) | 0.406 (0.385-0.428) | |
| **2002** | 0.441 (0.419-0.464) | 0.223 (0.211-0.237) | 0.267 (0.256-0.278) | 0.37 (0.351-0.391) | |
| **2003** | 0.438 (0.417-0.461) | 0.218 (0.206-0.232) | 0.283 (0.272-0.295) | 0.382 (0.362-0.403) | |
| **2004** | 0.451 (0.428-0.474) | 0.216 (0.204-0.23) | 0.278 (0.267-0.29) | 0.4 (0.38-0.422) | |
| **2005** | 0.434 (0.412-0.457) | 0.225 (0.212-0.238) | 0.284 (0.273-0.296) | 0.389 (0.369-0.41) | |
| **2006** | 0.424 (0.403-0.446) | 0.239 (0.225-0.253) | 0.287 (0.276-0.298) | 0.403 (0.382-0.425) | |
| **2007** | 0.427 (0.405-0.449) | 0.225 (0.212-0.238) | 0.29 (0.278-0.301) | 0.413 (0.392-0.434) | |
| **2008** | 0.443 (0.421-0.467) | 0.227 (0.214-0.24) | 0.296 (0.285-0.308) | 0.427 (0.406-0.45) | |
| **2009** | 0.435 (0.413-0.458) | 0.249 (0.235-0.263) | 0.301 (0.29-0.313) | 0.421 (0.4-0.443) | |
| **2010** | 0.424 (0.403-0.447) | 0.232 (0.219-0.246) | 0.296 (0.285-0.308) | 0.416 (0.395-0.438) | |
| **2011** | 0.416 (0.394-0.439) | 0.242 (0.228-0.256) | 0.307 (0.296-0.319) | 0.444 (0.422-0.467) | |
| **2012** | 0.407 (0.385-0.43) | 0.239 (0.225-0.253) | 0.304 (0.292-0.316) | 0.456 (0.433-0.48) | |
| **2013** | 0.428 (0.405-0.453) | 0.255 (0.241-0.27) | 0.312 (0.3-0.325) | 0.444 (0.421-0.467) | |
| **2014** | 0.427 (0.403-0.452) | 0.248 (0.234-0.263) | 0.302 (0.29-0.314) | 0.479 (0.455-0.505) | |
| **2015** | 0.442 (0.417-0.468) | 0.272 (0.257-0.289) | 0.323 (0.311-0.337) | 0.462 (0.439-0.487) | |
| **2016** | 0.441 (0.416-0.468) | 0.27 (0.254-0.287) | 0.344 (0.33-0.357) | 0.478 (0.453-0.504) | |
| **2017** | 0.479 (0.452-0.508) | 0.293 (0.276-0.312) | 0.346 (0.332-0.36) | 0.504 (0.478-0.531) | |
| **2018** | 0.449 (0.422-0.478) | 0.308 (0.29-0.327) | 0.355 (0.341-0.37) | 0.478 (0.453-0.505) | |
| **2019** | 0.455 (0.427-0.484) | 0.317 (0.298-0.337) | 0.359 (0.344-0.374) | 0.486 (0.46-0.513) | |
| **2020** | 0.477 (0.448-0.508) | 0.335 (0.315-0.356) | 0.397 (0.382-0.413) | 0.519 (0.492-0.547) | |
| **55-64 Age Group** | | | | | |
| **1999** | 0.383 (0.369-0.397) | 0.24 (0.231-0.249) | 0.266 (0.258-0.273) | 0.334 (0.321-0.347) | |
| **2000** | 0.392 (0.378-0.407) | 0.233 (0.224-0.243) | 0.263 (0.255-0.27) | 0.337 (0.324-0.35) | |
| **2001** | 0.397 (0.383-0.411) | 0.241 (0.232-0.25) | 0.271 (0.264-0.279) | 0.363 (0.349-0.377) | |
| **2002** | 0.383 (0.37-0.397) | 0.247 (0.238-0.257) | 0.266 (0.259-0.273) | 0.336 (0.324-0.349) | |
| **2003** | 0.384 (0.37-0.398) | 0.243 (0.234-0.252) | 0.272 (0.264-0.28) | 0.341 (0.329-0.354) | |
| **2004** | 0.393 (0.38-0.408) | 0.247 (0.238-0.257) | 0.274 (0.267-0.282) | 0.36 (0.347-0.374) | |
| **2005** | 0.4 (0.387-0.415) | 0.255 (0.246-0.265) | 0.279 (0.272-0.287) | 0.365 (0.352-0.378) | |
| **2006** | 0.398 (0.384-0.412) | 0.258 (0.248-0.267) | 0.289 (0.282-0.297) | 0.358 (0.345-0.371) | |
| **2007** | 0.412 (0.398-0.426) | 0.257 (0.248-0.267) | 0.295 (0.287-0.303) | 0.373 (0.36-0.386) | |
| **2008** | 0.399 (0.385-0.413) | 0.265 (0.256-0.275) | 0.299 (0.292-0.307) | 0.389 (0.376-0.403) | |
| **2009** | 0.398 (0.384-0.412) | 0.258 (0.249-0.268) | 0.304 (0.296-0.312) | 0.387 (0.374-0.4) | |
| **2010** | 0.401 (0.387-0.415) | 0.258 (0.249-0.267) | 0.307 (0.3-0.315) | 0.4 (0.387-0.413) | |
| **2011** | 0.397 (0.384-0.411) | 0.27 (0.26-0.279) | 0.309 (0.301-0.316) | 0.397 (0.384-0.41) | |
| **2012** | 0.382 (0.369-0.395) | 0.268 (0.259-0.278) | 0.314 (0.306-0.321) | 0.414 (0.401-0.428) | |
| **2013** | 0.395 (0.381-0.409) | 0.278 (0.269-0.288) | 0.323 (0.315-0.331) | 0.418 (0.405-0.432) | |
| **2014** | 0.388 (0.375-0.402) | 0.273 (0.264-0.283) | 0.323 (0.315-0.33) | 0.428 (0.415-0.442) | |
| **2015** | 0.392 (0.379-0.406) | 0.275 (0.266-0.284) | 0.321 (0.313-0.328) | 0.417 (0.405-0.431) | |
| **2016** | 0.4 (0.387-0.414) | 0.288 (0.279-0.298) | 0.334 (0.327-0.342) | 0.431 (0.418-0.445) | |
| **2017** | 0.407 (0.393-0.421) | 0.306 (0.296-0.316) | 0.348 (0.34-0.356) | 0.433 (0.42-0.447) | |
| **2018** | 0.407 (0.393-0.421) | 0.309 (0.299-0.319) | 0.357 (0.349-0.365) | 0.442 (0.429-0.456) | |
| **2019** | 0.414 (0.4-0.429) | 0.32 (0.31-0.33) | 0.364 (0.356-0.372) | 0.462 (0.448-0.476) | |
| **2020** | 0.45 (0.435-0.465) | 0.36 (0.349-0.371) | 0.402 (0.393-0.41) | 0.501 (0.487-0.516) | |
| **65-74 Age Group** | | | | | |
| **1999** | 0.424 (0.413-0.434) | 0.312 (0.304-0.32) | 0.32 (0.313-0.326) | 0.385 (0.375-0.396) | |
| **2000** | 0.427 (0.417-0.438) | 0.307 (0.299-0.315) | 0.319 (0.313-0.326) | 0.39 (0.379-0.401) | |
| **2001** | 0.427 (0.416-0.438) | 0.32 (0.311-0.328) | 0.325 (0.319-0.332) | 0.399 (0.388-0.41) | |
| **2002** | 0.426 (0.415-0.437) | 0.318 (0.309-0.326) | 0.333 (0.327-0.34) | 0.392 (0.381-0.403) | |
| **2003** | 0.424 (0.412-0.435) | 0.326 (0.318-0.335) | 0.332 (0.325-0.339) | 0.4 (0.389-0.411) | |
| **2004** | 0.44 (0.428-0.452) | 0.323 (0.314-0.332) | 0.328 (0.321-0.335) | 0.407 (0.395-0.418) | |
| **2005** | 0.435 (0.424-0.448) | 0.332 (0.323-0.341) | 0.329 (0.322-0.336) | 0.405 (0.394-0.417) | |
| **2006** | 0.44 (0.428-0.452) | 0.337 (0.328-0.347) | 0.344 (0.337-0.351) | 0.407 (0.396-0.419) | |
| **2007** | 0.446 (0.434-0.459) | 0.338 (0.329-0.348) | 0.344 (0.337-0.352) | 0.414 (0.402-0.426) | |
| **2008** | 0.446 (0.434-0.459) | 0.337 (0.328-0.346) | 0.346 (0.339-0.354) | 0.427 (0.415-0.439) | |
| **2009** | 0.434 (0.422-0.446) | 0.328 (0.319-0.338) | 0.348 (0.341-0.355) | 0.427 (0.415-0.439) | |
| **2010** | 0.439 (0.427-0.452) | 0.33 (0.321-0.34) | 0.353 (0.346-0.36) | 0.438 (0.426-0.45) | |
| **2011** | 0.431 (0.419-0.444) | 0.327 (0.318-0.336) | 0.357 (0.349-0.364) | 0.437 (0.426-0.449) | |
| **2012** | 0.429 (0.417-0.441) | 0.328 (0.32-0.337) | 0.358 (0.351-0.365) | 0.449 (0.437-0.461) | |
| **2013** | 0.426 (0.415-0.438) | 0.332 (0.323-0.341) | 0.367 (0.36-0.374) | 0.44 (0.429-0.452) | |
| **2014** | 0.423 (0.411-0.435) | 0.333 (0.325-0.342) | 0.363 (0.356-0.37) | 0.447 (0.435-0.458) | |
| **2015** | 0.426 (0.414-0.438) | 0.337 (0.329-0.346) | 0.361 (0.354-0.367) | 0.448 (0.437-0.459) | |
| **2016** | 0.426 (0.415-0.438) | 0.346 (0.338-0.355) | 0.378 (0.372-0.385) | 0.45 (0.439-0.461) | |
| **2017** | 0.429 (0.418-0.44) | 0.356 (0.348-0.365) | 0.392 (0.386-0.4) | 0.462 (0.451-0.473) | |
| **2018** | 0.434 (0.422-0.445) | 0.36 (0.352-0.369) | 0.404 (0.397-0.412) | 0.469 (0.458-0.48) | |
| **2019** | 0.438 (0.427-0.45) | 0.38 (0.371-0.389) | 0.413 (0.406-0.42) | 0.478 (0.467-0.489) | |
| **2020** | 0.493 (0.481-0.505) | 0.424 (0.414-0.433) | 0.462 (0.455-0.47) | 0.529 (0.518-0.541) | |
| **75-84 Age Group** | | | | | |
| **1999** | 0.491 (0.481-0.502) | 0.405 (0.396-0.413) | 0.399 (0.392-0.406) | 0.466 (0.456-0.477) | |
| **2000** | 0.496 (0.486-0.507) | 0.403 (0.395-0.412) | 0.401 (0.394-0.408) | 0.471 (0.461-0.482) | |
| **2001** | 0.492 (0.482-0.502) | 0.402 (0.394-0.411) | 0.397 (0.39-0.404) | 0.472 (0.462-0.483) | |
| **2002** | 0.503 (0.493-0.514) | 0.413 (0.404-0.422) | 0.41 (0.403-0.417) | 0.48 (0.469-0.491) | |
| **2003** | 0.493 (0.483-0.504) | 0.415 (0.406-0.424) | 0.409 (0.402-0.416) | 0.477 (0.467-0.488) | |
| **2004** | 0.499 (0.488-0.509) | 0.415 (0.406-0.424) | 0.407 (0.4-0.414) | 0.482 (0.472-0.493) | |
| **2005** | 0.507 (0.496-0.517) | 0.428 (0.419-0.437) | 0.415 (0.408-0.422) | 0.489 (0.479-0.5) | |
| **2006** | 0.51 (0.499-0.521) | 0.433 (0.424-0.442) | 0.415 (0.408-0.423) | 0.485 (0.474-0.496) | |
| **2007** | 0.517 (0.506-0.528) | 0.431 (0.421-0.44) | 0.42 (0.413-0.428) | 0.49 (0.479-0.502) | |
| **2008** | 0.51 (0.499-0.521) | 0.431 (0.422-0.44) | 0.423 (0.415-0.43) | 0.501 (0.489-0.512) | |
| **2009** | 0.511 (0.499-0.522) | 0.422 (0.413-0.431) | 0.423 (0.416-0.431) | 0.501 (0.489-0.512) | |
| **2010** | 0.518 (0.506-0.529) | 0.424 (0.415-0.434) | 0.432 (0.424-0.44) | 0.513 (0.502-0.525) | |
| **2011** | 0.511 (0.5-0.523) | 0.435 (0.425-0.445) | 0.43 (0.422-0.438) | 0.515 (0.504-0.527) | |
| **2012** | 0.495 (0.483-0.506) | 0.43 (0.421-0.44) | 0.436 (0.428-0.444) | 0.52 (0.508-0.532) | |
| **2013** | 0.493 (0.482-0.505) | 0.426 (0.416-0.436) | 0.437 (0.429-0.445) | 0.525 (0.513-0.537) | |
| **2014** | 0.5 (0.488-0.512) | 0.426 (0.416-0.436) | 0.427 (0.419-0.435) | 0.515 (0.503-0.527) | |
| **2015** | 0.498 (0.486-0.51) | 0.428 (0.418-0.438) | 0.429 (0.422-0.437) | 0.519 (0.507-0.531) | |
| **2016** | 0.494 (0.482-0.506) | 0.428 (0.418-0.438) | 0.448 (0.44-0.456) | 0.529 (0.518-0.541) | |
| **2017** | 0.497 (0.485-0.509) | 0.438 (0.428-0.448) | 0.455 (0.447-0.463) | 0.537 (0.525-0.549) | |
| **2018** | 0.506 (0.494-0.519) | 0.445 (0.435-0.455) | 0.466 (0.458-0.474) | 0.545 (0.533-0.557) | |
| **2019** | 0.503 (0.491-0.515) | 0.456 (0.446-0.466) | 0.487 (0.479-0.495) | 0.551 (0.539-0.563) | |
| **2020** | 0.576 (0.563-0.59) | 0.518 (0.508-0.529) | 0.536 (0.527-0.544) | 0.598 (0.586-0.61) | |
| **85+ Age Group** | | | | |  |
| **1999** | 0.595 (0.58-0.61) | 0.53 (0.517-0.544) | 0.499 (0.488-0.51) | 0.585 (0.569-0.602) | |
| **2000** | 0.592 (0.578-0.607) | 0.531 (0.518-0.545) | 0.498 (0.488-0.51) | 0.578 (0.562-0.594) | |
| **2001** | 0.598 (0.583-0.613) | 0.531 (0.518-0.545) | 0.511 (0.499-0.522) | 0.572 (0.556-0.588) | |
| **2002** | 0.593 (0.578-0.608) | 0.536 (0.523-0.549) | 0.506 (0.495-0.518) | 0.588 (0.572-0.604) | |
| **2003** | 0.596 (0.582-0.611) | 0.541 (0.528-0.555) | 0.507 (0.496-0.519) | 0.585 (0.569-0.601) | |
| **2004** | 0.594 (0.579-0.609) | 0.547 (0.534-0.561) | 0.504 (0.492-0.515) | 0.579 (0.563-0.595) | |
| **2005** | 0.59 (0.575-0.604) | 0.553 (0.54-0.567) | 0.513 (0.502-0.525) | 0.589 (0.574-0.605) | |
| **2006** | 0.593 (0.579-0.608) | 0.549 (0.536-0.563) | 0.512 (0.5-0.523) | 0.604 (0.589-0.62) | |
| **2007** | 0.605 (0.59-0.62) | 0.547 (0.534-0.56) | 0.515 (0.504-0.527) | 0.594 (0.579-0.609) | |
| **2008** | 0.591 (0.577-0.606) | 0.548 (0.535-0.561) | 0.511 (0.5-0.523) | 0.599 (0.583-0.614) | |
| **2009** | 0.586 (0.572-0.6) | 0.546 (0.533-0.56) | 0.515 (0.504-0.527) | 0.599 (0.584-0.614) | |
| **2010** | 0.595 (0.581-0.609) | 0.536 (0.523-0.549) | 0.517 (0.506-0.528) | 0.607 (0.592-0.622) | |
| **2011** | 0.605 (0.591-0.62) | 0.547 (0.534-0.56) | 0.519 (0.508-0.53) | 0.608 (0.594-0.623) | |
| **2012** | 0.598 (0.584-0.613) | 0.546 (0.533-0.559) | 0.52 (0.51-0.531) | 0.619 (0.604-0.634) | |
| **2013** | 0.586 (0.573-0.601) | 0.528 (0.516-0.541) | 0.522 (0.511-0.533) | 0.621 (0.606-0.636) | |
| **2014** | 0.586 (0.572-0.6) | 0.526 (0.514-0.539) | 0.493 (0.482-0.503) | 0.621 (0.607-0.636) | |
| **2015** | 0.579 (0.566-0.593) | 0.531 (0.519-0.544) | 0.497 (0.487-0.508) | 0.61 (0.596-0.624) | |
| **2016** | 0.576 (0.562-0.59) | 0.533 (0.521-0.546) | 0.505 (0.494-0.515) | 0.612 (0.598-0.627) | |
| **2017** | 0.581 (0.567-0.595) | 0.537 (0.525-0.55) | 0.524 (0.514-0.535) | 0.619 (0.605-0.633) | |
| **2018** | 0.583 (0.569-0.597) | 0.537 (0.525-0.55) | 0.54 (0.529-0.551) | 0.625 (0.611-0.639) | |
| **2019** | 0.58 (0.566-0.594) | 0.549 (0.536-0.562) | 0.555 (0.544-0.566) | 0.631 (0.617-0.645) | |
| **2020** | 0.658 (0.643-0.673) | 0.614 (0.6-0.628) | 0.607 (0.595-0.618) | 0.683 (0.669-0.698) | |

| **Supplemental Table 6** Proportional Mortality Ratio (95% CI) for cardiovascular co-listing on malignant neoplasm death certificates, stratified by race and ethnicity in the United States, 1999–2020 | | | | | |
| --- | --- | --- | --- | --- | --- |
| **Proportional Mortality Ratio (95% CI)** | | | | | |
| **Year** | **American Indian or Alaska Native** | **Asian or Pacific Islander** | **Black or African American** | **White** | **Hispanic or Latino** |
| **15-24 Age Group** | | | | | |
| **1999** | Suppressed | 3.318 (1.854-5.938) | 1.43 (1.074-1.903) | 1.749 (1.489-2.055) | 2.852 (2.127-3.824) |
| **2000** | Suppressed | 2.12 (1.227-3.664) | 1.926 (1.47-2.523) | 1.676 (1.416-1.984) | 2.651 (1.958-3.588) |
| **2001** | Suppressed | 2.56 (1.425-4.599) | 1.814 (1.353-2.431) | 1.678 (1.42-1.982) | 2.796 (2.092-3.738) |
| **2002** | Suppressed | 1.904 (1.02-3.554) | 1.621 (1.176-2.233) | 1.695 (1.435-2.002) | 2.865 (2.185-3.758) |
| **2003** | Suppressed | 2.183 (1.188-4.014) | 1.454 (1.054-2.006) | 1.478 (1.24-1.763) | 2.943 (2.218-3.903) |
| **2004** | Suppressed | 1.923 (1.063-3.477) | 1.712 (1.3-2.254) | 1.783 (1.509-2.107) | 2.975 (2.265-3.908) |
| **2005** | Suppressed | 2.068 (1.219-3.508) | 1.718 (1.271-2.322) | 1.53 (1.285-1.821) | 3.236 (2.517-4.162) |
| **2006** | Suppressed | 1.639 (0.852-3.152) | 1.73 (1.277-2.343) | 1.588 (1.329-1.897) | 2.81 (2.152-3.67) |
| **2007** | Suppressed | 1.821 (0.981-3.378) | 1.956 (1.47-2.602) | 1.908 (1.613-2.256) | 2.278 (1.739-2.985) |
| **2008** | Suppressed | 1.667 (0.938-2.962) | 1.5 (1.115-2.019) | 1.611 (1.345-1.93) | 2.238 (1.703-2.941) |
| **2009** | Suppressed | 1.569 (0.888-2.774) | 1.572 (1.186-2.082) | 1.666 (1.4-1.983) | 1.839 (1.381-2.449) |
| **2010** | Suppressed | 2.114 (1.237-3.612) | 1.569 (1.161-2.12) | 1.469 (1.226-1.76) | 2.303 (1.763-3.009) |
| **2011** | Suppressed | 2.24 (1.307-3.839) | 2.035 (1.536-2.696) | 1.503 (1.244-1.816) | 1.94 (1.496-2.516) |
| **2012** | Suppressed | 2.08 (1.265-3.42) | 1.397 (0.996-1.96) | 1.595 (1.324-1.922) | 1.884 (1.431-2.481) |
| **2013** | Suppressed | 0.945 (0.519-1.719) | 1.5 (1.082-2.079) | 1.571 (1.298-1.901) | 2.27 (1.741-2.958) |
| **2014** | Suppressed | 1.685 (0.955-2.974) | 1.933 (1.452-2.574) | 1.474 (1.218-1.784) | 2.321 (1.794-3.002) |
| **2015** | Suppressed | 1.401 (0.779-2.52) | 1.657 (1.204-2.281) | 1.669 (1.379-2.02) | 2.843 (2.214-3.649) |
| **2016** | Suppressed | 1.761 (0.991-3.13) | 1.818 (1.319-2.506) | 1.414 (1.143-1.749) | 3.2 (2.515-4.071) |
| **2017** | Suppressed | 1.647 (0.955-2.84) | 2.014 (1.471-2.758) | 1.918 (1.57-2.342) | 3.211 (2.532-4.071) |
| **2018** | Suppressed | 2.092 (1.198-3.654) | 2.102 (1.545-2.86) | 1.546 (1.26-1.895) | 2.334 (1.797-3.031) |
| **2019** | Suppressed | 1.79 (1.003-3.195) | 2.071 (1.515-2.83) | 1.671 (1.365-2.046) | 2.572 (2.038-3.247) |
| **2020** | Suppressed | 1.441 (0.79-2.628) | 2.339 (1.69-3.237) | 1.53 (1.233-1.897) | 2.51 (1.976-3.189) |
| **25-34 Age Group** | | | | | |
| **1999** | Suppressed | 0.97 (0.648-1.452) | 0.757 (0.629-0.91) | 0.762 (0.684-0.848) | 1.216 (0.978-1.513) |
| **2000** | Suppressed | 1.229 (0.825-1.832) | 0.893 (0.738-1.079) | 0.76 (0.678-0.851) | 1.42 (1.139-1.769) |
| **2001** | Suppressed | 0.985 (0.656-1.481) | 0.941 (0.786-1.126) | 0.751 (0.67-0.841) | 1.449 (1.164-1.804) |
| **2002** | Suppressed | 0.828 (0.554-1.238) | 0.726 (0.593-0.888) | 0.691 (0.613-0.778) | 0.999 (0.785-1.272) |
| **2003** | Suppressed | 0.738 (0.493-1.103) | 0.79 (0.652-0.958) | 0.744 (0.661-0.837) | 1.34 (1.085-1.656) |
| **2004** | Suppressed | 1.016 (0.689-1.499) | 0.779 (0.643-0.944) | 0.711 (0.63-0.804) | 1.333 (1.077-1.649) |
| **2005** | Suppressed | 0.939 (0.652-1.354) | 0.656 (0.536-0.804) | 0.768 (0.68-0.868) | 1.246 (1.002-1.548) |
| **2006** | Suppressed | 0.746 (0.505-1.103) | 0.759 (0.623-0.925) | 0.743 (0.657-0.84) | 1.305 (1.066-1.598) |
| **2007** | Suppressed | 0.998 (0.683-1.458) | 0.801 (0.658-0.975) | 0.833 (0.736-0.943) | 1.245 (1.02-1.52) |
| **2008** | Suppressed | 0.729 (0.498-1.067) | 0.757 (0.618-0.926) | 0.814 (0.719-0.921) | 1.035 (0.845-1.269) |
| **2009** | Suppressed | 0.693 (0.448-1.071) | 0.626 (0.507-0.773) | 0.737 (0.651-0.834) | 1.059 (0.872-1.285) |
| **2010** | Suppressed | 0.937 (0.647-1.357) | 0.725 (0.59-0.89) | 0.697 (0.615-0.791) | 1.032 (0.849-1.254) |
| **2011** | Suppressed | 0.618 (0.399-0.957) | 0.807 (0.669-0.974) | 0.779 (0.688-0.882) | 0.977 (0.795-1.2) |
| **2012** | Suppressed | 0.805 (0.559-1.16) | 0.752 (0.617-0.917) | 0.792 (0.701-0.896) | 1.036 (0.854-1.258) |
| **2013** | Suppressed | 0.901 (0.622-1.304) | 0.702 (0.576-0.855) | 0.752 (0.663-0.853) | 0.957 (0.79-1.158) |
| **2014** | Suppressed | 0.808 (0.554-1.18) | 0.716 (0.589-0.87) | 0.841 (0.746-0.948) | 0.944 (0.773-1.154) |
| **2015** | Suppressed | 0.953 (0.652-1.392) | 0.903 (0.75-1.088) | 0.819 (0.724-0.925) | 1.171 (0.979-1.4) |
| **2016** | Suppressed | 0.762 (0.525-1.106) | 0.841 (0.692-1.023) | 0.817 (0.722-0.925) | 1.216 (1.011-1.461) |
| **2017** | Suppressed | 0.86 (0.599-1.235) | 0.796 (0.655-0.968) | 0.926 (0.819-1.047) | 1.255 (1.055-1.494) |
| **2018** | Suppressed | 1.063 (0.766-1.476) | 0.83 (0.687-1.002) | 1.01 (0.9-1.132) | 1.276 (1.067-1.524) |
| **2019** | Suppressed | 0.905 (0.641-1.279) | 0.943 (0.785-1.132) | 0.974 (0.863-1.099) | 1.105 (0.926-1.318) |
| **2020** | Suppressed | 0.875 (0.626-1.221) | 0.997 (0.832-1.194) | 0.987 (0.879-1.109) | 1.225 (1.031-1.456) |
| **35-44 Age Group** | | | | | |
| **1999** | Suppressed | 0.521 (0.413-0.657) | 0.408 (0.372-0.446) | 0.384 (0.364-0.405) | 0.628 (0.548-0.721) |
| **2000** | Suppressed | 0.571 (0.459-0.711) | 0.46 (0.42-0.505) | 0.401 (0.379-0.423) | 0.747 (0.653-0.854) |
| **2001** | Suppressed | 0.467 (0.369-0.591) | 0.449 (0.409-0.493) | 0.398 (0.377-0.421) | 0.71 (0.621-0.813) |
| **2002** | Suppressed | 0.407 (0.319-0.52) | 0.456 (0.414-0.501) | 0.371 (0.35-0.392) | 0.646 (0.564-0.74) |
| **2003** | Suppressed | 0.48 (0.384-0.601) | 0.445 (0.404-0.489) | 0.383 (0.361-0.405) | 0.664 (0.581-0.76) |
| **2004** | Suppressed | 0.392 (0.305-0.504) | 0.42 (0.381-0.464) | 0.375 (0.353-0.398) | 0.598 (0.523-0.684) |
| **2005** | Suppressed | 0.36 (0.28-0.462) | 0.424 (0.383-0.469) | 0.402 (0.38-0.426) | 0.624 (0.547-0.712) |
| **2006** | Suppressed | 0.45 (0.359-0.563) | 0.421 (0.381-0.466) | 0.39 (0.367-0.414) | 0.66 (0.58-0.751) |
| **2007** | Suppressed | 0.444 (0.354-0.556) | 0.447 (0.404-0.495) | 0.389 (0.365-0.414) | 0.589 (0.517-0.671) |
| **2008** | Suppressed | 0.473 (0.378-0.592) | 0.417 (0.376-0.463) | 0.393 (0.368-0.419) | 0.59 (0.519-0.672) |
| **2009** | Suppressed | 0.454 (0.366-0.564) | 0.383 (0.343-0.427) | 0.389 (0.365-0.415) | 0.609 (0.537-0.692) |
| **2010** | Suppressed | 0.438 (0.35-0.548) | 0.366 (0.327-0.41) | 0.378 (0.353-0.404) | 0.604 (0.531-0.686) |
| **2011** | Suppressed | 0.424 (0.338-0.531) | 0.42 (0.378-0.467) | 0.395 (0.369-0.422) | 0.537 (0.471-0.613) |
| **2012** | Suppressed | 0.409 (0.33-0.507) | 0.405 (0.362-0.453) | 0.367 (0.342-0.393) | 0.521 (0.457-0.592) |
| **2013** | Suppressed | 0.386 (0.31-0.481) | 0.416 (0.372-0.465) | 0.396 (0.37-0.424) | 0.611 (0.542-0.689) |
| **2014** | Suppressed | 0.376 (0.299-0.472) | 0.388 (0.347-0.434) | 0.427 (0.399-0.457) | 0.626 (0.558-0.703) |
| **2015** | Suppressed | 0.412 (0.335-0.507) | 0.403 (0.36-0.451) | 0.422 (0.393-0.453) | 0.525 (0.464-0.593) |
| **2016** | Suppressed | 0.397 (0.323-0.487) | 0.432 (0.386-0.483) | 0.482 (0.45-0.516) | 0.631 (0.562-0.708) |
| **2017** | Suppressed | 0.46 (0.374-0.567) | 0.464 (0.416-0.517) | 0.505 (0.472-0.54) | 0.622 (0.554-0.698) |
| **2018** | Suppressed | 0.377 (0.302-0.47) | 0.441 (0.395-0.493) | 0.523 (0.489-0.559) | 0.628 (0.561-0.703) |
| **2019** | Suppressed | 0.481 (0.392-0.591) | 0.512 (0.462-0.568) | 0.528 (0.493-0.565) | 0.65 (0.582-0.726) |
| **2020** | Suppressed | 0.482 (0.395-0.587) | 0.512 (0.461-0.568) | 0.556 (0.521-0.593) | 0.671 (0.604-0.746) |
| **45-54 Age Group** | | | | | |
| **1999** | 0.445 (0.317-0.626) | 0.37 (0.318-0.43) | 0.342 (0.324-0.362) | 0.283 (0.275-0.292) | 0.423 (0.384-0.466) |
| **2000** | 0.412 (0.289-0.586) | 0.368 (0.317-0.428) | 0.347 (0.328-0.367) | 0.28 (0.272-0.289) | 0.443 (0.402-0.487) |
| **2001** | 0.339 (0.242-0.475) | 0.369 (0.32-0.426) | 0.357 (0.338-0.377) | 0.292 (0.283-0.301) | 0.483 (0.442-0.529) |
| **2002** | 0.35 (0.254-0.484) | 0.349 (0.302-0.402) | 0.338 (0.32-0.357) | 0.289 (0.28-0.298) | 0.451 (0.411-0.494) |
| **2003** | 0.3 (0.214-0.421) | 0.356 (0.309-0.41) | 0.35 (0.332-0.369) | 0.294 (0.285-0.303) | 0.434 (0.397-0.475) |
| **2004** | 0.37 (0.271-0.505) | 0.37 (0.322-0.424) | 0.343 (0.325-0.362) | 0.3 (0.291-0.309) | 0.446 (0.409-0.487) |
| **2005** | 0.365 (0.271-0.492) | 0.344 (0.299-0.395) | 0.344 (0.327-0.363) | 0.299 (0.291-0.309) | 0.429 (0.394-0.468) |
| **2006** | 0.392 (0.291-0.53) | 0.351 (0.305-0.403) | 0.335 (0.317-0.353) | 0.309 (0.3-0.318) | 0.432 (0.397-0.47) |
| **2007** | 0.39 (0.288-0.527) | 0.359 (0.312-0.412) | 0.338 (0.321-0.357) | 0.305 (0.296-0.315) | 0.442 (0.407-0.479) |
| **2008** | 0.368 (0.277-0.489) | 0.371 (0.324-0.425) | 0.352 (0.334-0.371) | 0.312 (0.303-0.322) | 0.439 (0.404-0.476) |
| **2009** | 0.345 (0.258-0.462) | 0.406 (0.356-0.463) | 0.354 (0.336-0.373) | 0.317 (0.308-0.327) | 0.44 (0.407-0.477) |
| **2010** | 0.336 (0.251-0.45) | 0.356 (0.312-0.407) | 0.335 (0.318-0.354) | 0.311 (0.302-0.321) | 0.419 (0.387-0.453) |
| **2011** | 0.43 (0.331-0.557) | 0.323 (0.283-0.369) | 0.343 (0.325-0.362) | 0.323 (0.314-0.333) | 0.419 (0.387-0.453) |
| **2012** | 0.452 (0.345-0.591) | 0.323 (0.283-0.37) | 0.337 (0.319-0.356) | 0.32 (0.311-0.33) | 0.433 (0.402-0.468) |
| **2013** | 0.444 (0.341-0.579) | 0.319 (0.28-0.365) | 0.352 (0.333-0.372) | 0.329 (0.319-0.339) | 0.429 (0.397-0.463) |
| **2014** | 0.359 (0.268-0.48) | 0.341 (0.3-0.388) | 0.341 (0.322-0.361) | 0.329 (0.319-0.339) | 0.451 (0.419-0.485) |
| **2015** | 0.388 (0.294-0.51) | 0.332 (0.292-0.378) | 0.354 (0.334-0.374) | 0.345 (0.334-0.356) | 0.458 (0.426-0.493) |
| **2016** | 0.49 (0.38-0.633) | 0.346 (0.304-0.393) | 0.351 (0.331-0.372) | 0.362 (0.35-0.373) | 0.454 (0.422-0.489) |
| **2017** | 0.502 (0.386-0.653) | 0.369 (0.327-0.417) | 0.377 (0.356-0.4) | 0.371 (0.359-0.383) | 0.493 (0.46-0.529) |
| **2018** | 0.463 (0.353-0.607) | 0.345 (0.304-0.392) | 0.377 (0.355-0.4) | 0.375 (0.363-0.387) | 0.462 (0.431-0.496) |
| **2019** | 0.498 (0.381-0.651) | 0.343 (0.303-0.388) | 0.377 (0.354-0.4) | 0.385 (0.373-0.398) | 0.455 (0.424-0.489) |
| **2020** | 0.475 (0.361-0.624) | 0.382 (0.34-0.43) | 0.444 (0.419-0.47) | 0.399 (0.386-0.412) | 0.518 (0.485-0.554) |
| **55-64 Age Group** | | | | | |
| **1999** | 0.339 (0.265-0.433) | 0.344 (0.305-0.387) | 0.341 (0.327-0.355) | 0.283 (0.277-0.288) | 0.37 (0.343-0.399) |
| **2000** | 0.38 (0.301-0.479) | 0.366 (0.325-0.411) | 0.343 (0.329-0.358) | 0.28 (0.275-0.286) | 0.39 (0.362-0.42) |
| **2001** | 0.325 (0.257-0.411) | 0.367 (0.328-0.412) | 0.36 (0.346-0.376) | 0.289 (0.283-0.295) | 0.405 (0.377-0.435) |
| **2002** | 0.345 (0.275-0.434) | 0.348 (0.31-0.389) | 0.348 (0.334-0.363) | 0.283 (0.278-0.289) | 0.372 (0.346-0.4) |
| **2003** | 0.312 (0.246-0.395) | 0.324 (0.289-0.364) | 0.346 (0.332-0.361) | 0.288 (0.282-0.294) | 0.37 (0.345-0.397) |
| **2004** | 0.369 (0.299-0.456) | 0.344 (0.308-0.385) | 0.349 (0.335-0.363) | 0.295 (0.289-0.3) | 0.387 (0.362-0.415) |
| **2005** | 0.315 (0.254-0.392) | 0.366 (0.33-0.406) | 0.348 (0.334-0.362) | 0.301 (0.296-0.307) | 0.391 (0.365-0.418) |
| **2006** | 0.365 (0.296-0.452) | 0.325 (0.293-0.362) | 0.364 (0.35-0.378) | 0.303 (0.297-0.309) | 0.383 (0.358-0.409) |
| **2007** | 0.351 (0.286-0.43) | 0.358 (0.323-0.396) | 0.362 (0.348-0.376) | 0.312 (0.306-0.318) | 0.391 (0.367-0.417) |
| **2008** | 0.336 (0.273-0.414) | 0.348 (0.314-0.385) | 0.356 (0.343-0.37) | 0.318 (0.312-0.324) | 0.398 (0.374-0.423) |
| **2009** | 0.406 (0.337-0.489) | 0.389 (0.354-0.428) | 0.358 (0.345-0.372) | 0.314 (0.308-0.32) | 0.381 (0.358-0.405) |
| **2010** | 0.363 (0.302-0.436) | 0.366 (0.333-0.402) | 0.349 (0.337-0.363) | 0.321 (0.315-0.327) | 0.401 (0.378-0.426) |
| **2011** | 0.378 (0.313-0.457) | 0.342 (0.312-0.375) | 0.357 (0.344-0.37) | 0.322 (0.316-0.327) | 0.41 (0.387-0.434) |
| **2012** | 0.377 (0.314-0.453) | 0.324 (0.295-0.356) | 0.356 (0.344-0.369) | 0.326 (0.32-0.332) | 0.4 (0.378-0.423) |
| **2013** | 0.362 (0.303-0.434) | 0.332 (0.303-0.363) | 0.37 (0.357-0.383) | 0.332 (0.326-0.338) | 0.418 (0.396-0.442) |
| **2014** | 0.371 (0.311-0.444) | 0.341 (0.312-0.373) | 0.361 (0.349-0.374) | 0.332 (0.326-0.338) | 0.427 (0.405-0.451) |
| **2015** | 0.421 (0.356-0.498) | 0.352 (0.322-0.383) | 0.352 (0.34-0.364) | 0.33 (0.325-0.336) | 0.421 (0.4-0.444) |
| **2016** | 0.372 (0.315-0.44) | 0.352 (0.323-0.383) | 0.366 (0.353-0.378) | 0.344 (0.338-0.35) | 0.424 (0.403-0.446) |
| **2017** | 0.405 (0.345-0.474) | 0.334 (0.307-0.364) | 0.377 (0.365-0.391) | 0.357 (0.351-0.363) | 0.43 (0.409-0.452) |
| **2018** | 0.426 (0.363-0.5) | 0.353 (0.325-0.383) | 0.384 (0.371-0.397) | 0.364 (0.357-0.37) | 0.424 (0.404-0.445) |
| **2019** | 0.456 (0.391-0.532) | 0.361 (0.332-0.391) | 0.393 (0.38-0.407) | 0.372 (0.365-0.378) | 0.44 (0.42-0.462) |
| **2020** | 0.528 (0.457-0.61) | 0.424 (0.394-0.458) | 0.447 (0.433-0.462) | 0.4 (0.394-0.407) | 0.517 (0.494-0.54) |
| **65-74 Age Group** | | | | | |
| **1999** | 0.313 (0.253-0.386) | 0.395 (0.361-0.433) | 0.389 (0.377-0.403) | 0.345 (0.34-0.349) | 0.402 (0.379-0.426) |
| **2000** | 0.372 (0.306-0.452) | 0.426 (0.389-0.466) | 0.383 (0.37-0.396) | 0.344 (0.34-0.349) | 0.414 (0.391-0.438) |
| **2001** | 0.366 (0.302-0.442) | 0.393 (0.36-0.43) | 0.396 (0.383-0.409) | 0.349 (0.345-0.354) | 0.442 (0.418-0.467) |
| **2002** | 0.327 (0.268-0.399) | 0.406 (0.371-0.444) | 0.393 (0.38-0.407) | 0.353 (0.348-0.358) | 0.408 (0.385-0.432) |
| **2003** | 0.377 (0.311-0.457) | 0.402 (0.368-0.439) | 0.398 (0.384-0.412) | 0.355 (0.35-0.36) | 0.418 (0.395-0.442) |
| **2004** | 0.366 (0.305-0.438) | 0.413 (0.379-0.45) | 0.406 (0.393-0.421) | 0.356 (0.351-0.361) | 0.403 (0.381-0.427) |
| **2005** | 0.344 (0.287-0.414) | 0.424 (0.39-0.462) | 0.404 (0.39-0.419) | 0.358 (0.353-0.363) | 0.394 (0.373-0.417) |
| **2006** | 0.365 (0.305-0.438) | 0.407 (0.374-0.443) | 0.406 (0.392-0.42) | 0.368 (0.363-0.373) | 0.405 (0.383-0.428) |
| **2007** | 0.382 (0.319-0.457) | 0.398 (0.366-0.434) | 0.416 (0.402-0.43) | 0.368 (0.363-0.374) | 0.422 (0.4-0.446) |
| **2008** | 0.408 (0.346-0.482) | 0.413 (0.381-0.449) | 0.41 (0.396-0.424) | 0.372 (0.367-0.378) | 0.422 (0.4-0.446) |
| **2009** | 0.388 (0.327-0.461) | 0.41 (0.378-0.445) | 0.407 (0.393-0.421) | 0.368 (0.362-0.373) | 0.423 (0.401-0.446) |
| **2010** | 0.406 (0.345-0.478) | 0.423 (0.391-0.458) | 0.416 (0.402-0.43) | 0.372 (0.367-0.377) | 0.437 (0.415-0.46) |
| **2011** | 0.403 (0.342-0.474) | 0.397 (0.367-0.43) | 0.41 (0.396-0.424) | 0.372 (0.367-0.377) | 0.435 (0.413-0.458) |
| **2012** | 0.381 (0.326-0.446) | 0.383 (0.354-0.415) | 0.402 (0.388-0.416) | 0.378 (0.373-0.384) | 0.433 (0.412-0.455) |
| **2013** | 0.422 (0.362-0.492) | 0.389 (0.36-0.42) | 0.405 (0.392-0.418) | 0.379 (0.373-0.384) | 0.442 (0.421-0.464) |
| **2014** | 0.423 (0.365-0.491) | 0.377 (0.35-0.407) | 0.401 (0.388-0.414) | 0.379 (0.374-0.384) | 0.431 (0.411-0.452) |
| **2015** | 0.408 (0.352-0.473) | 0.388 (0.361-0.417) | 0.398 (0.385-0.411) | 0.381 (0.376-0.386) | 0.432 (0.413-0.452) |
| **2016** | 0.417 (0.361-0.482) | 0.375 (0.349-0.402) | 0.402 (0.39-0.415) | 0.391 (0.386-0.397) | 0.449 (0.429-0.47) |
| **2017** | 0.452 (0.394-0.519) | 0.396 (0.37-0.424) | 0.416 (0.404-0.429) | 0.401 (0.396-0.406) | 0.453 (0.433-0.473) |
| **2018** | 0.498 (0.438-0.566) | 0.389 (0.364-0.416) | 0.414 (0.401-0.426) | 0.411 (0.406-0.417) | 0.449 (0.431-0.469) |
| **2019** | 0.477 (0.419-0.544) | 0.404 (0.379-0.431) | 0.427 (0.415-0.439) | 0.422 (0.417-0.427) | 0.45 (0.432-0.469) |
| **2020** | 0.557 (0.493-0.628) | 0.457 (0.431-0.485) | 0.494 (0.481-0.507) | 0.464 (0.458-0.469) | 0.548 (0.528-0.569) |
| **75-84 Age Group** | | | | | |
| **1999** | 0.403 (0.327-0.498) | 0.473 (0.434-0.515) | 0.458 (0.443-0.473) | 0.43 (0.426-0.435) | 0.472 (0.446-0.501) |
| **2000** | 0.438 (0.359-0.536) | 0.494 (0.455-0.536) | 0.449 (0.435-0.465) | 0.434 (0.429-0.439) | 0.47 (0.444-0.497) |
| **2001** | 0.361 (0.292-0.445) | 0.491 (0.454-0.532) | 0.462 (0.447-0.478) | 0.43 (0.425-0.434) | 0.47 (0.445-0.497) |
| **2002** | 0.397 (0.326-0.483) | 0.482 (0.446-0.522) | 0.458 (0.443-0.473) | 0.442 (0.438-0.447) | 0.48 (0.456-0.507) |
| **2003** | 0.472 (0.394-0.566) | 0.49 (0.454-0.528) | 0.464 (0.449-0.48) | 0.439 (0.435-0.444) | 0.457 (0.434-0.482) |
| **2004** | 0.417 (0.345-0.504) | 0.508 (0.471-0.547) | 0.464 (0.449-0.48) | 0.44 (0.435-0.445) | 0.481 (0.457-0.506) |
| **2005** | 0.412 (0.34-0.498) | 0.506 (0.47-0.545) | 0.473 (0.457-0.489) | 0.449 (0.444-0.454) | 0.49 (0.467-0.514) |
| **2006** | 0.479 (0.401-0.572) | 0.509 (0.474-0.548) | 0.469 (0.453-0.485) | 0.451 (0.446-0.456) | 0.473 (0.451-0.497) |
| **2007** | 0.423 (0.353-0.508) | 0.504 (0.47-0.541) | 0.474 (0.458-0.49) | 0.455 (0.449-0.46) | 0.495 (0.472-0.519) |
| **2008** | 0.468 (0.392-0.559) | 0.508 (0.473-0.544) | 0.476 (0.46-0.492) | 0.456 (0.451-0.461) | 0.488 (0.465-0.512) |
| **2009** | 0.462 (0.385-0.553) | 0.492 (0.459-0.528) | 0.477 (0.461-0.493) | 0.455 (0.449-0.46) | 0.472 (0.45-0.496) |
| **2010** | 0.488 (0.412-0.578) | 0.5 (0.467-0.535) | 0.477 (0.461-0.493) | 0.463 (0.458-0.469) | 0.485 (0.463-0.509) |
| **2011** | 0.514 (0.435-0.607) | 0.486 (0.454-0.521) | 0.48 (0.463-0.496) | 0.463 (0.458-0.469) | 0.487 (0.465-0.51) |
| **2012** | 0.474 (0.402-0.559) | 0.476 (0.445-0.509) | 0.475 (0.459-0.492) | 0.462 (0.456-0.467) | 0.496 (0.474-0.519) |
| **2013** | 0.482 (0.41-0.566) | 0.467 (0.437-0.499) | 0.479 (0.463-0.496) | 0.462 (0.456-0.467) | 0.498 (0.476-0.521) |
| **2014** | 0.462 (0.393-0.543) | 0.469 (0.439-0.501) | 0.463 (0.448-0.479) | 0.458 (0.453-0.464) | 0.488 (0.467-0.51) |
| **2015** | 0.49 (0.421-0.57) | 0.455 (0.427-0.486) | 0.459 (0.444-0.475) | 0.461 (0.455-0.466) | 0.492 (0.471-0.514) |
| **2016** | 0.48 (0.412-0.559) | 0.455 (0.427-0.485) | 0.482 (0.466-0.498) | 0.469 (0.463-0.475) | 0.491 (0.47-0.512) |
| **2017** | 0.551 (0.476-0.636) | 0.477 (0.448-0.507) | 0.471 (0.456-0.487) | 0.477 (0.472-0.483) | 0.492 (0.471-0.513) |
| **2018** | 0.51 (0.44-0.591) | 0.478 (0.45-0.508) | 0.472 (0.456-0.487) | 0.488 (0.483-0.494) | 0.496 (0.476-0.517) |
| **2019** | 0.528 (0.457-0.61) | 0.466 (0.439-0.495) | 0.48 (0.465-0.496) | 0.5 (0.494-0.506) | 0.509 (0.488-0.53) |
| **2020** | 0.645 (0.567-0.733) | 0.538 (0.509-0.568) | 0.555 (0.538-0.572) | 0.551 (0.545-0.557) | 0.598 (0.576-0.621) |
| **85+ Age Group** | | | | | |
| **1999** | 0.537 (0.397-0.727) | 0.643 (0.575-0.719) | 0.537 (0.513-0.561) | 0.544 (0.537-0.552) | 0.574 (0.532-0.619) |
| **2000** | 0.474 (0.353-0.637) | 0.628 (0.563-0.699) | 0.53 (0.507-0.554) | 0.544 (0.537-0.551) | 0.556 (0.515-0.599) |
| **2001** | 0.491 (0.369-0.655) | 0.625 (0.562-0.695) | 0.54 (0.517-0.565) | 0.547 (0.54-0.555) | 0.59 (0.549-0.634) |
| **2002** | 0.509 (0.385-0.674) | 0.629 (0.567-0.698) | 0.544 (0.52-0.568) | 0.55 (0.543-0.557) | 0.557 (0.519-0.598) |
| **2003** | 0.426 (0.315-0.576) | 0.629 (0.57-0.696) | 0.538 (0.514-0.562) | 0.553 (0.546-0.56) | 0.56 (0.522-0.602) |
| **2004** | 0.498 (0.376-0.659) | 0.614 (0.557-0.677) | 0.533 (0.509-0.558) | 0.552 (0.545-0.56) | 0.545 (0.508-0.586) |
| **2005** | 0.498 (0.374-0.663) | 0.608 (0.554-0.668) | 0.55 (0.526-0.575) | 0.557 (0.55-0.564) | 0.553 (0.517-0.592) |
| **2006** | 0.548 (0.421-0.712) | 0.583 (0.531-0.639) | 0.549 (0.525-0.574) | 0.56 (0.553-0.567) | 0.579 (0.542-0.619) |
| **2007** | 0.591 (0.465-0.752) | 0.583 (0.533-0.637) | 0.554 (0.53-0.579) | 0.561 (0.554-0.568) | 0.565 (0.53-0.603) |
| **2008** | 0.491 (0.377-0.64) | 0.591 (0.542-0.644) | 0.568 (0.544-0.594) | 0.557 (0.55-0.564) | 0.564 (0.529-0.601) |
| **2009** | 0.578 (0.449-0.745) | 0.585 (0.538-0.635) | 0.563 (0.538-0.588) | 0.557 (0.55-0.564) | 0.557 (0.523-0.592) |
| **2010** | 0.574 (0.455-0.723) | 0.581 (0.535-0.631) | 0.551 (0.527-0.575) | 0.561 (0.554-0.568) | 0.562 (0.529-0.597) |
| **2011** | 0.603 (0.478-0.761) | 0.565 (0.521-0.612) | 0.567 (0.543-0.592) | 0.566 (0.559-0.573) | 0.579 (0.546-0.613) |
| **2012** | 0.621 (0.492-0.784) | 0.575 (0.532-0.62) | 0.569 (0.545-0.593) | 0.567 (0.56-0.574) | 0.561 (0.531-0.593) |
| **2013** | 0.568 (0.455-0.709) | 0.567 (0.526-0.612) | 0.534 (0.512-0.558) | 0.563 (0.556-0.57) | 0.566 (0.536-0.598) |
| **2014** | 0.619 (0.498-0.769) | 0.584 (0.543-0.628) | 0.529 (0.507-0.553) | 0.553 (0.546-0.56) | 0.551 (0.523-0.581) |
| **2015** | 0.543 (0.439-0.673) | 0.55 (0.512-0.59) | 0.535 (0.513-0.558) | 0.551 (0.544-0.557) | 0.564 (0.536-0.594) |
| **2016** | 0.551 (0.442-0.687) | 0.533 (0.497-0.572) | 0.533 (0.511-0.557) | 0.555 (0.548-0.562) | 0.555 (0.527-0.583) |
| **2017** | 0.552 (0.444-0.686) | 0.556 (0.52-0.594) | 0.518 (0.496-0.541) | 0.567 (0.56-0.574) | 0.559 (0.533-0.587) |
| **2018** | 0.563 (0.457-0.693) | 0.542 (0.507-0.579) | 0.535 (0.513-0.558) | 0.573 (0.566-0.58) | 0.578 (0.551-0.606) |
| **2019** | 0.643 (0.526-0.784) | 0.547 (0.513-0.583) | 0.536 (0.514-0.559) | 0.582 (0.575-0.59) | 0.568 (0.543-0.595) |
| **2020** | 0.661 (0.547-0.799) | 0.607 (0.571-0.645) | 0.622 (0.599-0.647) | 0.641 (0.633-0.648) | 0.643 (0.616-0.672) |

| **Supplemental Table 7** Proportional Mortality Ratio (95% CI) for cardiovascular co-listing on malignant neoplasm death certificates, stratified by place of death in the United States, 1999–2020 | | | | | | | |
| --- | --- | --- | --- | --- | --- | --- | --- |
| **Deaths and Proportional Mortality Ratio (95% CI)** | | | | | | | |
| **Cohort** | **Cancer and CVD Deaths (A)** | **Total Cancer Deaths (B)** | **Total CVD Deaths (C)** | **Total Non-Cancer Deaths (D)** | **A/B** | **C/D** | **PMR** |
| **15-24 Age Group** | | | | | | | |
| Medical Facility | 5070 | 21503 | 46946 | 288062 | 0.236 | 0.163 | 1.447 |
| Decedent's home | 1196 | 11787 | 12048 | 127152 | 0.101 | 0.095 | 1.071 |
| Hospice facility | 97 | 1271 | 318 | 1595 | 0.076 | 0.199 | 0.383 |
| Nursing home/long term care | 61 | 530 | 710 | 2930 | 0.115 | 0.242 | 0.475 |
| Other/Unknown | 138 | 1166 | 5348 | 240483 | 0.118 | 0.022 | 5.322 |
| **25-34 Age Group** | | | | | | | |
| Medical Facility | 9997 | 44192 | 120134 | 411993 | 0.226 | 0.292 | 0.776 |
| Decedent's home | 2713 | 28101 | 44760 | 261051 | 0.097 | 0.171 | 0.563 |
| Hospice facility | 446 | 5341 | 1336 | 5293 | 0.084 | 0.252 | 0.331 |
| Nursing home/long term care | 369 | 2742 | 2742 | 9574 | 0.135 | 0.286 | 0.470 |
| Other/Unknown | 462 | 4549 | 14710 | 270207 | 0.102 | 0.054 | 1.866 |
| **35-44 Age Group** | | | | | | | |
| Medical Facility | 30208 | 134125 | 340976 | 721485 | 0.225 | 0.473 | 0.477 |
| Decedent's home | 11898 | 110035 | 147746 | 441732 | 0.108 | 0.334 | 0.323 |
| Hospice facility | 1898 | 20174 | 4062 | 13988 | 0.094 | 0.290 | 0.324 |
| Nursing home/long term care | 2186 | 15483 | 11026 | 32287 | 0.141 | 0.341 | 0.413 |
| Other/Unknown | 1779 | 17145 | 39535 | 269564 | 0.104 | 0.147 | 0.707 |
| **45-54 Age Group** | | | | | | | |
| Medical Facility | 106961 | 429048 | 885792 | 1470353 | 0.249 | 0.602 | 0.414 |
| Decedent's home | 54513 | 419255 | 453677 | 869757 | 0.130 | 0.522 | 0.249 |
| Hospice facility | 8711 | 78172 | 15319 | 43508 | 0.111 | 0.352 | 0.316 |
| Nursing home/long term care | 12801 | 72928 | 47658 | 104481 | 0.176 | 0.456 | 0.385 |
| Other/Unknown | 7302 | 58216 | 95973 | 323964 | 0.125 | 0.296 | 0.423 |
| **55-64 Age Group** | | | | | | | |
| Medical Facility | 269843 | 919704 | 1642859 | 2411695 | 0.293 | 0.681 | 0.431 |
| Decedent's home | 169510 | 1003610 | 884758 | 1329860 | 0.169 | 0.665 | 0.254 |
| Hospice facility | 26934 | 195554 | 45981 | 103321 | 0.138 | 0.445 | 0.309 |
| Nursing home/long term care | 46867 | 205917 | 167024 | 291954 | 0.228 | 0.572 | 0.398 |
| Other/Unknown | 19994 | 121749 | 140186 | 301675 | 0.164 | 0.465 | 0.353 |
| **65-74 Age Group** | | | | | | | |
| Medical Facility | 443687 | 1270669 | 2510441 | 3486925 | 0.349 | 0.720 | 0.485 |
| Decedent's home | 323353 | 1487497 | 1185867 | 1652812 | 0.217 | 0.717 | 0.303 |
| Hospice facility | 48661 | 273717 | 108268 | 202893 | 0.178 | 0.534 | 0.333 |
| Nursing home/long term care | 115864 | 402846 | 512114 | 813743 | 0.288 | 0.629 | 0.457 |
| Other/Unknown | 35835 | 176098 | 152671 | 267088 | 0.203 | 0.572 | 0.356 |
| **75-84 Age Group** | | | | | | | |
| Medical Facility | 492047 | 1198084 | 3640625 | 4984408 | 0.411 | 0.730 | 0.562 |
| Decedent's home | 426322 | 1563255 | 1702129 | 2351387 | 0.273 | 0.724 | 0.377 |
| Hospice facility | 59250 | 268190 | 236116 | 400263 | 0.221 | 0.590 | 0.375 |
| Nursing home/long term care | 247810 | 707285 | 1686726 | 2597245 | 0.350 | 0.649 | 0.540 |
| Other/Unknown | 55258 | 220058 | 265355 | 433047 | 0.251 | 0.613 | 0.410 |
| **85+ Age Group** | | | | | | | |
| Medical Facility | 251642 | 522351 | 3290364 | 4549782 | 0.482 | 0.723 | 0.666 |
| Decedent's home | 297897 | 856139 | 2153245 | 2897641 | 0.348 | 0.743 | 0.468 |
| Hospice facility | 44212 | 161049 | 388249 | 607046 | 0.275 | 0.640 | 0.429 |
| Nursing home/long term care | 311638 | 730385 | 3879260 | 5749665 | 0.427 | 0.675 | 0.632 |
| Other/Unknown | 51808 | 157213 | 494359 | 747729 | 0.330 | 0.661 | 0.498 |

| **Supplementary Table 8: Death counts and Proportional Mortality Ratio (95% CI) for cardiovascular co-listing on lung cancer death certificates, stratified by age group and demographic subgroup in the United States, 1999–2020** | | | | | | | |  |
| --- | --- | --- | --- | --- | --- | --- | --- | --- |
| **Deaths and Proportional Mortality Ratio (95% CI)** | | | | | | | |  |
| **Cohort** | **Lung Cancer and CVD Deaths (A)** | **Total Lung Cancer Deaths (B)** | **Total CVD Deaths (C)** | **All-Cause Deaths (D)** | **A/B** | **C/D** | **PMR (95% CI)** |  |
| **15-24 Age Group** | | | | | | | |  |
| Overall | 109 | 574 | 71932 | 696479 | 0.190 | 0.103 | 1.839 (1.498-2.257) |  |
| Female | 48 | 239 | 25714 | 182871 | 0.201 | 0.141 | 1.428 (1.047-1.948) |  |
| Male | 61 | 335 | 46218 | 513608 | 0.182 | 0.090 | 2.024 (1.54-2.659) |  |
| NH American Indian | Suppressed | Suppressed | Suppressed | Suppressed | Suppressed | Suppressed | Suppressed |  |
| NH Asian Pacific | 8 | 26 | 2354 | 17506 | 0.308 | 0.134 | 2.288 (1.035-5.06) |  |
| NH Black | 25 | 122 | 18505 | 156196 | 0.205 | 0.118 | 1.73 (1.125-2.66) |  |
| NH White | 51 | 307 | 38523 | 389760 | 0.166 | 0.099 | 1.681 (1.249-2.261) |  |
| Hispanic | 25 | 112 | 11448 | 118666 | 0.223 | 0.096 | 2.314 (1.499-3.571) |  |
| Northeast | 20 | 93 | 11330 | 99222 | 0.215 | 0.114 | 1.883 (1.161-3.054) |  |
| Midwest | 15 | 110 | 14325 | 151729 | 0.136 | 0.094 | 1.444 (0.842-2.478) |  |
| South | 37 | 222 | 30401 | 292574 | 0.167 | 0.104 | 1.604 (1.132-2.272) |  |
| West | 37 | 149 | 15876 | 152954 | 0.248 | 0.104 | 2.392 (1.668-3.43) |  |
| Metro | 92 | 477 | 58871 | 571297 | 0.193 | 0.103 | 1.872 (1.497-2.34) |  |
| Non metro | 17 | 97 | 13061 | 125182 | 0.175 | 0.104 | 1.68 (1.003-2.813) |  |
| **25-34 Age Group** | | | | | | | |  |
| Overall | 556 | 3287 | 197669 | 1043043 | 0.169 | 0.190 | 0.893 (0.816-0.977) |  |
| Female | 252 | 1468 | 73210 | 317873 | 0.172 | 0.230 | 0.745 (0.652-0.852) |  |
| Male | 304 | 1819 | 124459 | 725170 | 0.167 | 0.172 | 0.974 (0.862-1.1) |  |
| NH American Indian | 6 | 14 | 3247 | 18799 | 0.429 | 0.173 | 2.481 (0.953-6.462) |  |
| NH Asian Pacific | 43 | 242 | 5837 | 25727 | 0.178 | 0.227 | 0.783 (0.566-1.085) |  |
| NH Black | 114 | 553 | 54190 | 228840 | 0.206 | 0.237 | 0.871 (0.711-1.065) |  |
| NH White | 297 | 1994 | 106559 | 613934 | 0.149 | 0.174 | 0.858 (0.76-0.97) |  |
| Hispanic | 96 | 471 | 27267 | 152348 | 0.204 | 0.179 | 1.139 (0.914-1.419) |  |
| Northeast | 142 | 609 | 30308 | 162275 | 0.233 | 0.187 | 1.248 (1.04-1.499) |  |
| Midwest | 71 | 693 | 39020 | 218478 | 0.102 | 0.179 | 0.574 (0.449-0.733) |  |
| South | 202 | 1337 | 86053 | 442618 | 0.151 | 0.194 | 0.777 (0.67-0.901) |  |
| West | 141 | 648 | 42288 | 219672 | 0.218 | 0.193 | 1.13 (0.942-1.357) |  |
| Metro | 475 | 2776 | 163401 | 870520 | 0.171 | 0.188 | 0.912 (0.827-1.005) |  |
| Non metro | 81 | 511 | 34268 | 172523 | 0.159 | 0.199 | 0.798 (0.631-1.009) |  |
| **35-44 Age Group** | | | | | | | |  |
| Overall | 5657 | 37108 | 591314 | 1783349 | 0.152 | 0.332 | 0.46 (0.447-0.473) |  |
| Female | 2711 | 17708 | 216042 | 657934 | 0.153 | 0.328 | 0.466 (0.448-0.486) |  |
| Male | 2946 | 19400 | 375272 | 1125415 | 0.152 | 0.333 | 0.455 (0.438-0.473) |  |
| NH American Indian | 28 | 199 | 8131 | 27471 | 0.141 | 0.296 | 0.475 (0.32-0.707) |  |
| NH Asian Pacific | 278 | 1383 | 16065 | 42385 | 0.201 | 0.379 | 0.53 (0.466-0.604) |  |
| NH Black | 1120 | 6383 | 153115 | 370955 | 0.175 | 0.413 | 0.425 (0.399-0.453) |  |
| NH White | 3736 | 26956 | 345816 | 1128801 | 0.139 | 0.306 | 0.452 (0.437-0.468) |  |
| Hispanic | 473 | 2086 | 66081 | 207045 | 0.227 | 0.319 | 0.71 (0.643-0.785) |  |
| Northeast | 1466 | 7020 | 94303 | 285852 | 0.209 | 0.330 | 0.633 (0.598-0.67) |  |
| Midwest | 999 | 8996 | 118991 | 372149 | 0.111 | 0.320 | 0.347 (0.325-0.371) |  |
| South | 2193 | 15827 | 258328 | 760726 | 0.139 | 0.340 | 0.408 (0.39-0.427) |  |
| West | 999 | 5265 | 119692 | 364622 | 0.190 | 0.328 | 0.578 (0.54-0.619) |  |
| Metro | 4626 | 29915 | 485353 | 1475982 | 0.155 | 0.329 | 0.47 (0.456-0.485) |  |
| Non metro | 1031 | 7193 | 105961 | 307367 | 0.143 | 0.345 | 0.416 (0.389-0.444) |  |
| **45-54 Age Group** | | | | | | | |  |
| Overall | 41425 | 237408 | 1688707 | 3869682 | 0.174 | 0.436 | 0.4 (0.396-0.404) |  |
| Female | 17753 | 104281 | 592787 | 1483779 | 0.170 | 0.400 | 0.426 (0.419-0.433) |  |
| Male | 23672 | 133127 | 1095920 | 2385903 | 0.178 | 0.459 | 0.387 (0.382-0.393) |  |
| NH American Indian | 377 | 1629 | 17999 | 44101 | 0.231 | 0.408 | 0.567 (0.506-0.635) |  |
| NH Asian Pacific | 1235 | 5501 | 39904 | 85699 | 0.225 | 0.466 | 0.482 (0.453-0.513) |  |
| NH Black | 8142 | 39487 | 388127 | 744429 | 0.206 | 0.521 | 0.395 (0.386-0.405) |  |
| NH White | 29657 | 181922 | 1085268 | 2640418 | 0.163 | 0.411 | 0.397 (0.392-0.402) |  |
| Hispanic | 2010 | 8498 | 150497 | 339146 | 0.237 | 0.444 | 0.533 (0.508-0.56) |  |
| Northeast | 9988 | 42460 | 282998 | 638744 | 0.235 | 0.443 | 0.531 (0.519-0.543) |  |
| Midwest | 7802 | 58868 | 349657 | 837077 | 0.133 | 0.418 | 0.317 (0.31-0.325) |  |
| South | 16538 | 102582 | 719654 | 1623761 | 0.161 | 0.443 | 0.364 (0.358-0.37) |  |
| West | 7097 | 33498 | 336398 | 770100 | 0.212 | 0.437 | 0.485 (0.473-0.498) |  |
| Metro | 33046 | 188920 | 1379063 | 3175567 | 0.175 | 0.434 | 0.403 (0.398-0.408) |  |
| Non metro | 8379 | 48488 | 309644 | 694115 | 0.173 | 0.446 | 0.387 (0.378-0.397) |  |
| **55-64 Age Group** | | | | | | | |  |
| Overall | 147406 | 698802 | 3413956 | 6885039 | 0.211 | 0.496 | 0.425 (0.423-0.428) |  |
| Female | 57522 | 289478 | 1239376 | 2718374 | 0.199 | 0.456 | 0.436 (0.432-0.44) |  |
| Male | 89884 | 409324 | 2174580 | 4166665 | 0.220 | 0.522 | 0.421 (0.418-0.424) |  |
| NH American Indian | 882 | 3996 | 29288 | 58938 | 0.221 | 0.497 | 0.444 (0.412-0.478) |  |
| NH Asian Pacific | 3248 | 12885 | 76338 | 146387 | 0.252 | 0.521 | 0.483 (0.465-0.503) |  |
| NH Black | 23757 | 95403 | 666271 | 1157831 | 0.249 | 0.575 | 0.433 (0.426-0.439) |  |
| NH White | 113537 | 563796 | 2376345 | 5021170 | 0.201 | 0.473 | 0.426 (0.423-0.428) |  |
| Hispanic | 5552 | 20970 | 250791 | 473412 | 0.265 | 0.530 | 0.5 (0.485-0.515) |  |
| Northeast | 31703 | 121560 | 586151 | 1148457 | 0.261 | 0.510 | 0.511 (0.505-0.518) |  |
| Midwest | 29908 | 170654 | 718988 | 1529325 | 0.175 | 0.470 | 0.373 (0.368-0.378) |  |
| South | 59072 | 297999 | 1430197 | 2867979 | 0.198 | 0.499 | 0.398 (0.394-0.401) |  |
| West | 26723 | 108589 | 678620 | 1339278 | 0.246 | 0.507 | 0.486 (0.479-0.492) |  |
| Metro | 116011 | 550102 | 2749327 | 5556283 | 0.211 | 0.495 | 0.426 (0.423-0.429) |  |
| Non metro | 31395 | 148700 | 664629 | 1328756 | 0.211 | 0.500 | 0.422 (0.417-0.427) |  |
| **65-74 Age Group** | | | | | | | |  |
| Overall | 290081 | 1142068 | 5436761 | 10034288 | 0.254 | 0.542 | 0.469 (0.467-0.471) |  |
| Female | 114278 | 483673 | 2244594 | 4348898 | 0.236 | 0.516 | 0.458 (0.455-0.461) |  |
| Male | 175803 | 658395 | 3192167 | 5685390 | 0.267 | 0.561 | 0.476 (0.473-0.478) |  |
| NH American Indian | 1418 | 5649 | 36239 | 64742 | 0.251 | 0.560 | 0.448 (0.422-0.476) |  |
| NH Asian Pacific | 5980 | 20169 | 123773 | 211916 | 0.296 | 0.584 | 0.508 (0.493-0.523) |  |
| NH Black | 33814 | 117325 | 804351 | 1309819 | 0.288 | 0.614 | 0.469 (0.464-0.475) |  |
| NH White | 237764 | 962264 | 4109156 | 7835707 | 0.247 | 0.524 | 0.471 (0.469-0.473) |  |
| Hispanic | 10328 | 34070 | 344198 | 580953 | 0.303 | 0.592 | 0.512 (0.5-0.523) |  |
| Northeast | 60540 | 199990 | 991172 | 1754344 | 0.303 | 0.565 | 0.536 (0.531-0.541) |  |
| Midwest | 61313 | 275883 | 1176110 | 2280440 | 0.222 | 0.516 | 0.431 (0.427-0.435) |  |
| South | 111684 | 472374 | 2196329 | 4086490 | 0.236 | 0.537 | 0.44 (0.437-0.443) |  |
| West | 56544 | 193821 | 1073150 | 1913014 | 0.292 | 0.561 | 0.52 (0.515-0.525) |  |
| Metro | 227359 | 896824 | 4325213 | 7988242 | 0.254 | 0.541 | 0.468 (0.466-0.47) |  |
| Non metro | 62722 | 245244 | 1111548 | 2046046 | 0.256 | 0.543 | 0.471 (0.467-0.475) |  |
| **75-84 Age Group** | | | | | | | |  |
| Overall | 313454 | 1078114 | 8811638 | 14723222 | 0.291 | 0.598 | 0.486 (0.484-0.488) |  |
| Female | 134068 | 486916 | 4374987 | 7382049 | 0.275 | 0.593 | 0.465 (0.462-0.467) |  |
| Male | 179386 | 591198 | 4436651 | 7341173 | 0.303 | 0.604 | 0.502 (0.499-0.505) |  |
| NH American Indian | 1122 | 3824 | 36771 | 61070 | 0.293 | 0.602 | 0.487 (0.455-0.521) |  |
| NH Asian Pacific | 7272 | 20919 | 191772 | 297725 | 0.348 | 0.644 | 0.54 (0.525-0.555) |  |
| NH Black | 26524 | 85188 | 884453 | 1367405 | 0.311 | 0.647 | 0.481 (0.475-0.488) |  |
| NH White | 266948 | 933634 | 7215497 | 12244092 | 0.286 | 0.589 | 0.485 (0.483-0.487) |  |
| Hispanic | 10984 | 32687 | 462615 | 721210 | 0.336 | 0.641 | 0.524 (0.513-0.535) |  |
| Northeast | 70121 | 208339 | 1775939 | 2839920 | 0.337 | 0.625 | 0.538 (0.534-0.543) |  |
| Midwest | 67898 | 260093 | 2007048 | 3472785 | 0.261 | 0.578 | 0.452 (0.448-0.456) |  |
| South | 109144 | 410733 | 3277507 | 5592404 | 0.266 | 0.586 | 0.453 (0.45-0.457) |  |
| West | 66291 | 198949 | 1751144 | 2818113 | 0.333 | 0.621 | 0.536 (0.531-0.541) |  |
| Metro | 251604 | 867206 | 7063713 | 11821779 | 0.290 | 0.598 | 0.486 (0.483-0.488) |  |
| Non metro | 61850 | 210908 | 1747925 | 2901443 | 0.293 | 0.602 | 0.487 (0.482-0.491) |  |
| **85+ Age Group** | | | | | | | |  |
| Overall | 130831 | 404186 | 11162674 | 16979000 | 0.324 | 0.657 | 0.492 (0.489-0.495) |  |
| Female | 65718 | 207272 | 7182135 | 10883756 | 0.317 | 0.660 | 0.48 (0.476-0.485) |  |
| Male | 65113 | 196914 | 3980539 | 6095244 | 0.331 | 0.653 | 0.506 (0.502-0.511) |  |
| NH American Indian | 321 | 1044 | 26972 | 42672 | 0.307 | 0.632 | 0.486 (0.429-0.552) |  |
| NH Asian Pacific | 3712 | 9714 | 226433 | 328516 | 0.382 | 0.689 | 0.554 (0.534-0.576) |  |
| NH Black | 8909 | 27277 | 785500 | 1169730 | 0.327 | 0.672 | 0.486 (0.475-0.498) |  |
| NH White | 112756 | 352068 | 9633475 | 14718613 | 0.320 | 0.655 | 0.489 (0.486-0.493) |  |
| Hispanic | 4902 | 13389 | 470114 | 689855 | 0.366 | 0.681 | 0.537 (0.52-0.555) |  |
| Northeast | 31779 | 86871 | 2484855 | 3627082 | 0.366 | 0.685 | 0.534 (0.527-0.541) |  |
| Midwest | 28111 | 95050 | 2696400 | 4199415 | 0.296 | 0.642 | 0.461 (0.454-0.467) |  |
| South | 40246 | 140385 | 3699313 | 5807840 | 0.287 | 0.637 | 0.45 (0.445-0.455) |  |
| West | 30695 | 81880 | 2282106 | 3344663 | 0.375 | 0.682 | 0.549 (0.542-0.557) |  |
| Metro | 108100 | 334337 | 9037541 | 13772027 | 0.323 | 0.656 | 0.493 (0.489-0.496) |  |
| Non metro | 22731 | 69849 | 2125133 | 3206973 | 0.325 | 0.663 | 0.491 (0.484-0.499) |  |

| **Supplementary Table 9: Average annual percent change (AAPC) in the Proportional Mortality Ratio for cardiovascular co-listing on lung cancer death certificates, by age group and demographic subgroup in the United States, 1999–2020** | | | | | |
| --- | --- | --- | --- | --- | --- |
| **Proportional Mortality Ratio AAPC (1999-2020)** | | | | | |
| **Age Group** | **AAPC (95% CI)** | **p-value** | **Age Group** | **AAPC (95% CI)** | **p-value** |
| **Overall** | | | | | |
| 15-24 | Suppressed | Suppressed | 55-64 | 1.13* (0.98-1.25) | < 0.000001 |
| 25-34 | 1.86* (1.03-2.92) | < 0.000001 | 65-74 | 1.13* (0.97-1.25) | < 0.000001 |
| 35-44 | 1.90* (1.55-2.23) | < 0.000001 | 75-84 | 1.16* (1.05-1.37) | < 0.000001 |
| 45-54 | 1.32* (1.06-1.56) | < 0.000001 | 85+ | 1.00* (0.9-1.15) | < 0.000001 |
| **CVD Burden in Lung Cancer Deaths (A/B)** | | | | | |
| 15-24 | Suppressed | Suppressed | 55-64 | 1.19* (1.05-1.33) | < 0.000001 |
| 25-34 | 1.62* (0.61-2.86) | 0.004799 | 65-74 | 0.93* (0.82-1.03) | < 0.000001 |
| 35-44 | 1.89* (1.49-2.32) | < 0.000001 | 75-84 | 0.63* (0.51-0.73) | < 0.000001 |
| 45-54 | 1.46* (1.26-1.69) | < 0.000001 | 85+ | 0.39* (0.3-0.48) | < 0.000001 |
| **CVD Burden All Cause Deaths (C/D)** | | | | | |
| 15-24 | Suppressed | Suppressed | 55-64 | -0.01 (-0.03-0.02) | 0.713457 |
| 25-34 | -0.17 (-0.47-0.09) | 0.153169 | 65-74 | -0.18* (-0.21--0.16) | < 0.000001 |
| 35-44 | -0.03 (-0.12-0.09) | 0.492701 | 75-84 | -0.53* (-0.56--0.5) | < 0.000001 |
| 45-54 | 0.16* (0.09-0.25) | < 0.000001 | 85+ | -0.58* (-0.62--0.55) | < 0.000001 |
| **Female** | | | | | |
| 15-24 | Suppressed | Suppressed | 55-64 | 1.05* (0.78-1.28) | < 0.000001 |
| 25-34 | Suppressed | Suppressed | 65-74 | 1.15* (0.85-1.33) | < 0.000001 |
| 35-44 | 1.77* (1.09-2.42) | < 0.000001 | 75-84 | 1.24* (1.1-1.35) | < 0.000001 |
| 45-54 | 1.29* (0.93-1.61) | < 0.000001 | 85+ | 0.94* (0.62-1.14) | < 0.000001 |
| **Male** | | | | | |
| 15-24 | Suppressed | Suppressed | 55-64 | 1.23* (1.08-1.37) | < 0.000001 |
| 25-34 | Suppressed | Suppressed | 65-74 | 1.08* (0.92-1.21) | < 0.000001 |
| 35-44 | 2.07* (1.64-2.46) | < 0.000001 | 75-84 | 1.01* (0.91-1.16) | < 0.000001 |
| 45-54 | 1.34* (1.14-1.55) | < 0.000001 | 85+ | 0.90* (0.75-1.05) | < 0.000001 |
| **NH American Indian or Alaskan Native** | | | | | |
| 15-24 | Suppressed | Suppressed | 55-64 | 2.32* (1.25-3.4) | 0.008798 |
| 25-34 | Suppressed | Suppressed | 65-74 | 2.07* (1.4-3.09) | < 0.000001 |
| 35-44 | Suppressed | Suppressed | 75-84 | 2.01* (1.44-2.9) | < 0.000001 |
| 45-54 | -1.77* (-3.11--0.58) | 0.007598 | 85+ | Suppressed | Suppressed |
| **NH Asian or Pacific Islander** | | | | | |
| 15-24 | Suppressed | Suppressed | 55-64 | 0.77* (0-1.39) | 0.04999 |
| 25-34 | Suppressed | Suppressed | 65-74 | 0.17 (-0.13-0.75) | 0.245951 |
| 35-44 | Suppressed | Suppressed | 75-84 | -0.1 (-0.44-0.31) | 0.65227 |
| 45-54 | 0.94 (-0.01-2.11) | 0.05199 | 85+ | 0.25 (-0.38-1.32) | 0.304339 |
| **NH Black or African American** | | | | | |
| 15-24 | Suppressed | Suppressed | 55-64 | 0.93* (0.54-1.3) | < 0.000001 |
| 25-34 | Suppressed | Suppressed | 65-74 | 1.00* (0.64-1.22) | < 0.000001 |
| 35-44 | 0.46 (-0.71-1.33) | 0.513497 | 75-84 | 1.12* (0.79-1.3) | < 0.000001 |
| 45-54 | 1.66* (0.84-2.05) | < 0.000001 | 85+ | 0.85* (0.51-1.29) | 0.0004 |
| **NH White** | | | | | |
| 15-24 | Suppressed | Suppressed | 55-64 | 1.14* (1.02-1.25) | < 0.000001 |
| 25-34 | Suppressed | Suppressed | 65-74 | 1.16* (1.04-1.34) | < 0.000001 |
| 35-44 | 2.37* (1.84-2.89) | < 0.000001 | 75-84 | 1.23* (1.11-1.33) | < 0.000001 |
| 45-54 | 1.43* (1.03-1.76) | < 0.000001 | 85+ | 1.00* (0.85-1.13) | < 0.000001 |
| **Hispanic or Latino** | | | | | |
| 15-24 | Suppressed | Suppressed | 55-64 | 0.39 (-0.1-1.13) | 0.122376 |
| 25-34 | Suppressed | Suppressed | 65-74 | 1.03* (0.43-1.46) | 0.0004 |
| 35-44 | 0.44 (-0.66-1.67) | 0.411118 | 75-84 | 0.70* (0.35-1.14) | 0.0012 |
| 45-54 | 0.48 (-0.17-1.18) | 0.145571 | 85+ | 0.99* (0.57-1.59) | 0.0004 |
| **Northeast region** | | | | | |
| 15-24 | Suppressed | Suppressed | Suppressed | 0.25* (0.09-0.42) | 0.0024 |
| 25-34 | Suppressed | Suppressed | Suppressed | 0.49* (0.3-0.63) | < 0.000001 |
| 35-44 | 0.79 (-0.08-1.48) | 0.078784 | 75-84 | 0.50* (0.35-0.65) | < 0.000001 |
| 45-54 | 0.38 (-0.13-0.85) | 0.159568 | 85+ | 0.41* (0.06-0.63) | 0.023995 |
| **Midwest region** | | | | | |
| 15-24 | Suppressed | Suppressed | 55-64 | 1.28* (1.12-1.44) | < 0.000001 |
| 25-34 | Suppressed | Suppressed | 65-74 | 1.09* (0.91-1.3) | < 0.000001 |
| 35-44 | 3.54* (2.6-4.54) | < 0.000001 | 75-84 | 1.23* (1.05-1.51) | < 0.000001 |
| 45-54 | 1.98* (1.21-2.74) | < 0.000001 | 85+ | 0.81* (0.5-1.07) | < 0.000001 |
| **South region** | | | | | |
| 15-24 | Suppressed | Suppressed | 55-64 | 1.62* (1.4-1.82) | < 0.000001 |
| 25-34 | Suppressed | Suppressed | 65-74 | 1.50* (1.35-1.66) | < 0.000001 |
| 35-44 | 1.99* (1.15-2.79) | < 0.000001 | 75-84 | 1.46* (1.3-1.63) | < 0.000001 |
| 45-54 | 1.53* (1.25-1.81) | < 0.000001 | 85+ | 1.20* (1.09-1.32) | < 0.000001 |
| **West region** | | | | | |
| 15-24 | Suppressed | Suppressed | 55-64 | 1.22* (1.02-1.47) | < 0.000001 |
| 25-34 | Suppressed | Suppressed | 65-74 | 1.39* (1.22-1.59) | < 0.000001 |
| 35-44 | 1.79* (0.92-2.79) | < 0.000001 | 75-84 | 1.11* (1.03-1.21) | < 0.000001 |
| 45-54 | 1.17* (0.66-1.84) | 0.0004 | 85+ | 1.33* (1.2-1.5) | < 0.000001 |
| **Metropolitan area** | | | | | |
| 15-24 | Suppressed | Suppressed | 55-64 | 1.07* (0.9-1.22) | < 0.000001 |
| 25-34 | 1.42* (0.22-2.88) | 0.027195 | 65-74 | 1.08* (0.92-1.2) | < 0.000001 |
| 35-44 | 1.86* (1.47-2.27) | < 0.000001 | 75-84 | 1.17* (0.94-1.3) | < 0.000001 |
| 45-54 | 1.18* (0.91-1.44) | < 0.000001 | 85+ | 0.96* (0.88-1.06) | < 0.000001 |
| **Nonmetropolitan areas** | | | | | |
| 15-24 | Suppressed | Suppressed | 55-64 | 1.20* (0.98-1.4) | < 0.000001 |
| 25-34 | Suppressed | Suppressed | 65-74 | 1.30* (1.12-1.45) | < 0.000001 |
| 35-44 | 1.41 (-0.07-2.66) | 0.060788 | 75-84 | 1.41* (1.28-1.58) | < 0.000001 |
| 45-54 | 1.71* (1.14-2.13) | < 0.000001 | 85+ | 1.10* (0.76-1.41) | < 0.000001 |
| AAPC = average annual percent change; N.H. = non-Hispanic; * Indicates that the average annual percentage change (AAPC) is significantly different from zero at α = 0.05. | | | | | |

| **Supplementary Table 10: Death counts and Proportional Mortality Ratio (95% CI) for cardiovascular co-listing on leukaemia death certificates, stratified by age group and demographic subgroup in the United States, 1999–2020** | | | | | | | |
| --- | --- | --- | --- | --- | --- | --- | --- |
| **Deaths and Proportional Mortality Ratio (95% CI)** | | | | | | | |
| **Cohort** | **Leukemia and CVD Deaths (A)** | **Total Leukemia Deaths (B)** | **Total CVD Deaths (C-A)** | **All-Cause Deaths (D-B)** | **A/B** | **C-A/D-B** | **PMR (95% CI)** |
| **15-24 Age Group** | | | | | | | |
| Overall | 2107 | 9384 | 71932 | 696479 | 0.225 | 0.103 | 2.174 (2.072-2.281) |
| Female | 794 | 3560 | 25714 | 182871 | 0.223 | 0.141 | 1.586 (1.467-1.715) |
| Male | 1313 | 5824 | 46218 | 513608 | 0.225 | 0.09 | 2.505 (2.358-2.662) |
| NH American Indian | 16 | 107 | 935 | 12351 | 0.15 | 0.076 | 1.975 (1.163-3.354) |
| NH Asian Pacific | 119 | 403 | 2354 | 17506 | 0.295 | 0.134 | 2.196 (1.782-2.706) |
| NH Black | 341 | 1311 | 18505 | 156196 | 0.26 | 0.118 | 2.195 (1.947-2.476) |
| NH White | 947 | 4859 | 38523 | 389760 | 0.195 | 0.099 | 1.972 (1.838-2.116) |
| Hispanic | 683 | 2685 | 11448 | 118666 | 0.254 | 0.096 | 2.637 (2.419-2.874) |
| Northeast | 1462 | 6118 | 11330 | 99222 | 0.239 | 0.114 | 2.093 (1.97-2.223) |
| Midwest | 286 | 1802 | 14325 | 151729 | 0.159 | 0.094 | 1.681 (1.482-1.907) |
| South | 732 | 3512 | 30401 | 292574 | 0.208 | 0.104 | 2.006 (1.851-2.174) |
| West | 691 | 2595 | 15876 | 152954 | 0.266 | 0.104 | 2.565 (2.355-2.794) |
| Metro | 1851 | 8079 | 58871 | 571297 | 0.229 | 0.103 | 2.223 (2.112-2.34) |
| Non metro | 256 | 1305 | 13061 | 125182 | 0.196 | 0.104 | 1.88 (1.642-2.152) |
| **25-34 Age Group** | | | | | | | |
| Overall | 2299 | 10308 | 197669 | 1043043 | 0.223 | 0.19 | 1.177 (1.125-1.232) |
| Female | 955 | 4199 | 73210 | 317873 | 0.227 | 0.23 | 0.988 (0.92-1.06) |
| Male | 1344 | 6109 | 124459 | 725170 | 0.22 | 0.172 | 1.282 (1.208-1.36) |
| NH American Indian | 20 | 101 | 3247 | 18799 | 0.198 | 0.173 | 1.146 (0.709-1.855) |
| NH Asian Pacific | 145 | 527 | 5837 | 25727 | 0.275 | 0.227 | 1.213 (1.007-1.461) |
| NH Black | 378 | 1536 | 54190 | 228840 | 0.246 | 0.237 | 1.039 (0.928-1.163) |
| NH White | 1174 | 5766 | 106559 | 613934 | 0.204 | 0.174 | 1.173 (1.101-1.249) |
| Hispanic | 578 | 2360 | 27267 | 152348 | 0.245 | 0.179 | 1.368 (1.248-1.5) |
| Northeast | 517 | 1722 | 30308 | 162275 | 0.3 | 0.187 | 1.608 (1.456-1.775) |
| Midwest | 320 | 2037 | 39020 | 218478 | 0.157 | 0.179 | 0.88 (0.781-0.99) |
| South | 805 | 3942 | 86053 | 442618 | 0.204 | 0.194 | 1.05 (0.973-1.133) |
| West | 657 | 2607 | 42288 | 219672 | 0.252 | 0.193 | 1.309 (1.201-1.427) |
| Metro | 1999 | 8839 | 163401 | 870520 | 0.226 | 0.188 | 1.205 (1.147-1.265) |
| Non metro | 300 | 1469 | 34268 | 172523 | 0.204 | 0.199 | 1.028 (0.908-1.165) |
| **35-44 Age Group** | | | | | | | |
| Overall | 3501 | 15495 | 591314 | 1783349 | 0.226 | 0.332 | 0.681 (0.657-0.707) |
| Female | 1586 | 6965 | 216042 | 657934 | 0.228 | 0.328 | 0.693 (0.657-0.732) |
| Male | 1915 | 8530 | 375272 | 1125415 | 0.225 | 0.333 | 0.673 (0.641-0.708) |
| NH American Indian | 24 | 102 | 8131 | 27471 | 0.235 | 0.296 | 0.795 (0.509-1.241) |
| NH Asian Pacific | 177 | 695 | 16065 | 42385 | 0.255 | 0.379 | 0.672 (0.569-0.793) |
| NH Black | 607 | 2352 | 153115 | 370955 | 0.258 | 0.413 | 0.625 (0.572-0.684) |
| NH White | 1978 | 9568 | 345816 | 1128801 | 0.207 | 0.306 | 0.675 (0.643-0.708) |
| Hispanic | 704 | 2735 | 66081 | 207045 | 0.257 | 0.319 | 0.806 (0.742-0.877) |
| Northeast | 825 | 2802 | 94303 | 285852 | 0.294 | 0.33 | 0.892 (0.826-0.965) |
| Midwest | 509 | 3134 | 118991 | 372149 | 0.162 | 0.32 | 0.508 (0.462-0.558) |
| South | 1257 | 5926 | 258328 | 760726 | 0.212 | 0.34 | 0.625 (0.588-0.664) |
| West | 910 | 3633 | 119692 | 364622 | 0.25 | 0.328 | 0.763 (0.709-0.821) |
| Metro | 3010 | 13167 | 485353 | 1475982 | 0.229 | 0.329 | 0.695 (0.668-0.723) |
| Non metro | 491 | 2328 | 105961 | 307367 | 0.211 | 0.345 | 0.612 (0.555-0.675) |
| **45-54 Age Group** | | | | | | | |
| Overall | 7591 | 31906 | 1688707 | 3869682 | 0.238 | 0.436 | 0.545 (0.532-0.559) |
| Female | 3116 | 13860 | 592787 | 1483779 | 0.225 | 0.4 | 0.563 (0.541-0.585) |
| Male | 4475 | 18046 | 1095920 | 2385903 | 0.248 | 0.459 | 0.54 (0.522-0.558) |
| NH American Indian | 37 | 189 | 17999 | 44101 | 0.196 | 0.408 | 0.48 (0.337-0.683) |
| NH Asian Pacific | 325 | 1128 | 39904 | 85699 | 0.288 | 0.466 | 0.619 (0.547-0.7) |
| NH Black | 1188 | 4485 | 388127 | 744429 | 0.265 | 0.521 | 0.508 (0.477-0.542) |
| NH White | 5078 | 22700 | 1085268 | 2640418 | 0.224 | 0.411 | 0.544 (0.528-0.561) |
| Hispanic | 933 | 3325 | 150497 | 339146 | 0.281 | 0.444 | 0.632 (0.588-0.68) |
| Northeast | 1705 | 5770 | 282998 | 638744 | 0.295 | 0.443 | 0.667 (0.632-0.704) |
| Midwest | 1316 | 7212 | 349657 | 837077 | 0.182 | 0.418 | 0.437 (0.412-0.463) |
| South | 2694 | 12065 | 719654 | 1623761 | 0.223 | 0.443 | 0.504 (0.483-0.525) |
| West | 1876 | 6859 | 336398 | 770100 | 0.274 | 0.437 | 0.626 (0.595-0.659) |
| Metro | 6456 | 26704 | 1379063 | 3175567 | 0.242 | 0.434 | 0.557 (0.542-0.572) |
| Non metro | 1135 | 5202 | 309644 | 694115 | 0.218 | 0.446 | 0.489 (0.459-0.522) |
| **55-64 Age Group** | | | | | | | |
| Overall | 18582 | 70270 | 3413956 | 6885039 | 0.264 | 0.496 | 0.533 (0.525-0.542) |
| Female | 6803 | 27251 | 1239376 | 2718374 | 0.25 | 0.456 | 0.548 (0.533-0.562) |
| Male | 11779 | 43019 | 2174580 | 4166665 | 0.274 | 0.522 | 0.525 (0.514-0.535) |
| NH American Indian | 93 | 349 | 29288 | 58938 | 0.266 | 0.497 | 0.536 (0.426-0.674) |
| NH Asian Pacific | 572 | 1792 | 76338 | 146387 | 0.319 | 0.521 | 0.612 (0.557-0.673) |
| NH Black | 2544 | 8186 | 666271 | 1157831 | 0.311 | 0.575 | 0.54 (0.517-0.565) |
| NH White | 13907 | 55157 | 2376345 | 5021170 | 0.252 | 0.473 | 0.533 (0.523-0.543) |
| Hispanic | 1406 | 4605 | 250791 | 473412 | 0.305 | 0.53 | 0.576 (0.543-0.612) |
| Northeast | 4105 | 13060 | 586151 | 1148457 | 0.314 | 0.51 | 0.616 (0.595-0.638) |
| Midwest | 3564 | 16399 | 718988 | 1529325 | 0.217 | 0.47 | 0.462 (0.446-0.479) |
| South | 6644 | 26592 | 1430197 | 2867979 | 0.25 | 0.499 | 0.501 (0.488-0.515) |
| West | 4269 | 14219 | 678620 | 1339278 | 0.3 | 0.507 | 0.593 (0.573-0.613) |
| Metro | 15303 | 57437 | 2749327 | 5556283 | 0.266 | 0.495 | 0.538 (0.529-0.548) |
| Non metro | 3279 | 12833 | 664629 | 1328756 | 0.256 | 0.5 | 0.511 (0.492-0.531) |
| **65-74 Age Group** | | | | | | | |
| Overall | 39803 | 133312 | 5436761 | 10034288 | 0.299 | 0.542 | 0.551 (0.545-0.557) |
| Female | 13622 | 49670 | 2244594 | 4348898 | 0.274 | 0.516 | 0.531 (0.521-0.542) |
| Male | 26181 | 83642 | 3192167 | 5685390 | 0.313 | 0.561 | 0.557 (0.55-0.565) |
| NH American Indian | 190 | 537 | 36239 | 64742 | 0.354 | 0.56 | 0.632 (0.535-0.746) |
| NH Asian Pacific | 914 | 2703 | 123773 | 211916 | 0.338 | 0.584 | 0.579 (0.537-0.624) |
| NH Black | 3894 | 11435 | 804351 | 1309819 | 0.341 | 0.614 | 0.555 (0.535-0.575) |
| NH White | 32603 | 112125 | 4109156 | 7835707 | 0.291 | 0.524 | 0.554 (0.548-0.561) |
| Hispanic | 2131 | 6256 | 344198 | 580953 | 0.341 | 0.592 | 0.575 (0.547-0.604) |
| Northeast | 8570 | 24666 | 991172 | 1754344 | 0.347 | 0.565 | 0.615 (0.6-0.63) |
| Midwest | 8372 | 32214 | 1176110 | 2280440 | 0.26 | 0.516 | 0.504 (0.492-0.516) |
| South | 13934 | 49838 | 2196329 | 4086490 | 0.28 | 0.537 | 0.52 (0.51-0.53) |
| West | 8927 | 26594 | 1073150 | 1913014 | 0.336 | 0.561 | 0.598 (0.584-0.613) |
| Metro | 32095 | 107144 | 4325213 | 7988242 | 0.3 | 0.541 | 0.553 (0.546-0.56) |
| Non metro | 7708 | 26168 | 1111548 | 2046046 | 0.295 | 0.543 | 0.542 (0.529-0.556) |
| **75-84 Age Group** | | | | | | | |
| Overall | 65287 | 186947 | 8811638 | 14723222 | 0.35 | 0.6 | 0.584 (0.578-0.589) |
| Female | 25392 | 76462 | 4374987 | 7382049 | 0.33 | 0.59 | 0.56 (0.552-0.568) |
| Male | 39895 | 110485 | 4436651 | 7341173 | 0.36 | 0.6 | 0.597 (0.591-0.604) |
| NH American Indian | 177 | 473 | 36771 | 61070 | 0.37 | 0.6 | 0.621 (0.523-0.739) |
| NH Asian Pacific | 1176 | 3092 | 191772 | 297725 | 0.38 | 0.64 | 0.59 (0.552-0.632) |
| NH Black | 4242 | 11292 | 884453 | 1367405 | 0.38 | 0.65 | 0.581 (0.561-0.602) |
| NH White | 56837 | 164719 | 7215497 | 12244092 | 0.35 | 0.59 | 0.586 (0.58-0.591) |
| Hispanic | 2728 | 7072 | 462615 | 721210 | 0.39 | 0.64 | 0.601 (0.575-0.629) |
| Northeast | 14369 | 36476 | 1775939 | 2839920 | 0.39 | 0.63 | 0.63 (0.618-0.642) |
| Midwest | 15111 | 47233 | 2007048 | 3472785 | 0.32 | 0.58 | 0.554 (0.543-0.564) |
| South | 21409 | 65890 | 3277507 | 5592404 | 0.32 | 0.59 | 0.554 (0.546-0.563) |
| West | 14398 | 37348 | 1751144 | 2818113 | 0.39 | 0.62 | 0.62 (0.609-0.633) |
| Metro | 52269 | 150707 | 7063713 | 11821779 | 0.35 | 0.6 | 0.58 (0.575-0.586) |
| Non metro | 13018 | 36240 | 1747925 | 2901443 | 0.36 | 0.6 | 0.596 (0.584-0.608) |
| **85+ Age Group** | | | | | | | |
| Overall | 56305 | 132383 | 11162674 | 16979000 | 0.425 | 0.657 | 0.647 (0.641-0.653) |
| Female | 29366 | 69301 | 7182135 | 10883756 | 0.424 | 0.66 | 0.642 (0.633-0.651) |
| Male | 26939 | 63082 | 3980539 | 6095244 | 0.427 | 0.653 | 0.654 (0.645-0.663) |
| NH American Indian | 94 | 200 | 26972 | 42672 | 0.47 | 0.632 | 0.744 (0.582-0.951) |
| NH Asian Pacific | 677 | 1620 | 226433 | 328516 | 0.418 | 0.689 | 0.606 (0.554-0.663) |
| NH Black | 2684 | 6373 | 785500 | 1169730 | 0.421 | 0.672 | 0.627 (0.599-0.656) |
| NH White | 51152 | 120210 | 9633475 | 14718613 | 0.426 | 0.655 | 0.65 (0.643-0.657) |
| Hispanic | 1611 | 3788 | 470114 | 689855 | 0.425 | 0.681 | 0.624 (0.589-0.662) |
| Northeast | 12786 | 27492 | 2484855 | 3627082 | 0.465 | 0.685 | 0.679 (0.665-0.693) |
| Midwest | 14206 | 34831 | 2696400 | 4199415 | 0.408 | 0.642 | 0.635 (0.623-0.648) |
| South | 16664 | 42789 | 3699313 | 5807840 | 0.389 | 0.637 | 0.611 (0.601-0.622) |
| West | 12649 | 27271 | 2282106 | 3344663 | 0.464 | 0.682 | 0.68 (0.666-0.694) |
| Metro | 45003 | 106816 | 9037541 | 13772027 | 0.421 | 0.656 | 0.642 (0.635-0.649) |
| Non metro | 11302 | 25567 | 2125133 | 3206973 | 0.442 | 0.663 | 0.667 (0.652-0.682) |

| **Supplementary Table 11: Average annual percent change (AAPC) in the Proportional Mortality Ratio for cardiovascular co-listing on leukaemia death certificates, by age group and demographic subgroup in the United States, 1999–2020** | | | | | |
| --- | --- | --- | --- | --- | --- |
| **Proportional Mortality Ratio AAPC (1999-2020)** | | | | | |
| **Age Group** | **AAPC (95% CI)** | **p-value** | **Age Group** | **AAPC (95% CI)** | **p-value** |
| **Overall** | | | | | |
| 15-24 | 0.92 (-0.03-1.88) | 0.053189 | 55-64 | 1.18* (0.96-1.38) | < 0.000001 |
| 25-34 | 0.57 (-0.41-1.65) | 0.223155 | 65-74 | 1.13* (0.86-1.36) | < 0.000001 |
| 35-44 | 0.77 (-0.14-1.59) | 0.096381 | 75-84 | 1.16* (1.01-1.29) | < 0.000001 |
| 45-54 | 0.96* (0.61-1.35) | < 0.000001 | 85+ | 0.89* (0.67-1.05) | < 0.000001 |
| **CVD Burden in Leukemia Deaths (A/B)** | | | | | |
| 15-24 | 0.84* (0.33-1.38) | 0.003199 | 55-64 | 1.30* (1.04-1.5) | < 0.000001 |
| 25-34 | 0.34 (-0.41-1.15) | 0.365527 | 65-74 | 0.91* (0.72-1.13) | < 0.000001 |
| 35-44 | 0.76* (0.23-1.33) | 0.007199 | 75-84 | 0.57* (0.41-0.68) | < 0.000001 |
| 45-54 | 1.21* (0.93-1.52) | < 0.000001 | 85+ | 0.21* (0.02-0.36) | 0.031994 |
| **CVD Burden All Cause Deaths (C/D)** | | | | | |
| 15-24 | -0.21 (-0.52-0.14) | 0.137173 | 55-64 | -0.01 (-0.03-0.02) | 0.713457 |
| 25-34 | -0.17 (-0.47-0.09) | 0.153169 | 65-74 | -0.18* (-0.21--0.16) | < 0.000001 |
| 35-44 | -0.03 (-0.12-0.09) | 0.492701 | 75-84 | -0.53* (-0.56--0.5) | < 0.000001 |
| 45-54 | 0.16* (0.09-0.25) | < 0.000001 | 85+ | -0.58* (-0.62--0.55) | < 0.000001 |
| **Female** | | | | | |
| 15-24 | -0.54 (-2.29-1.35) | 0.546291 | 55-64 | 1.51* (0.95-1.86) | < 0.000001 |
| 25-34 | 0.07 (-1.03-1.19) | 0.931814 | 65-74 | 1.11* (0.83-1.41) | < 0.000001 |
| 35-44 | -0.11 (-0.97-0.7) | 0.689462 | 75-84 | 0.94* (0.53-1.18) | < 0.000001 |
| 45-54 | 0.49 (-0.08-1.09) | 0.09878 | 85+ | 0.77* (0.49-0.94) | < 0.000001 |
| **Male** | | | | | |
| 15-24 | 1.19* (0.26-2.01) | 0.014397 | 55-64 | 1.02* (0.71-1.38) | < 0.000001 |
| 25-34 | 1.64 (-0.13-3.45) | 0.053989 | 65-74 | 0.76* (0.48-1.23) | < 0.000001 |
| 35-44 | 1.59* (0.59-2.43) | 0.002 | 75-84 | 1.20* (0.99-1.36) | < 0.000001 |
| 45-54 | 1.27* (0.94-1.64) | < 0.000001 | 85+ | 0.90* (0.53-1.15) | < 0.000001 |
| **NH Asian or Pacific Islander** | | | | | |
| 15-24 | - | - | 55-64 | 0.57 (-0.48-2.07) | 0.209958 |
| 25-34 | - | - | 65-74 | 0.84* (0.02-2.09) | 0.046391 |
| 35-44 | - | - | 75-84 | 0.71* (0.01-1.76) | 0.046391 |
| 45-54 | 0.27 (-1.37-2.26) | 0.637872 | 85+ | -0.64 (-1.69-0.84) | 0.482304 |
| **NH Black or African American** | | | | | |
| 15-24 |  |  | 55-64 | 0.97* (0.43-1.67) | 0.0016 |
| 25-34 | 0.41 (-1.02-2) | 0.539492 | 65-74 | 0.74* (0.14-1.49) | 0.021996 |
| 35-44 | -0.74 (-1.87-0.28) | 0.14797 | 75-84 | 0.51 (-0.22-1) | 0.117976 |
| 45-54 | 0.04 (-1.25-1.4) | 0.958208 | 85+ | 0.45 (-0.3-1.36) | 0.187562 |
| **NH White** | | | | | |
| 15-24 | 0.98* (0.06-1.88) | 0.034793 | 55-64 | 1.43* (1.09-1.64) | < 0.000001 |
| 25-34 | 0.42 (-0.52-1.35) | 0.380724 | 65-74 | 1.13* (0.89-1.37) | < 0.000001 |
| 35-44 | 1.26* (0.35-2.18) | 0.007598 | 75-84 | 1.24* (1.09-1.37) | < 0.000001 |
| 45-54 | 1.18* (0.73-1.68) | < 0.000001 | 85+ | 0.94* (0.77-1.08) | < 0.000001 |
| **Hispanic or Latino** | | | | | |
| 15-24 | -0.89 (-1.97-0.04) | 0.060388 | 55-64 | 0.3 (-0.73-1.76) | 0.406719 |
| 25-34 | -0.07 (-1.47-1.54) | 0.981004 | 65-74 | 0.88* (0.1-1.99) | 0.033193 |
| 35-44 | -1.21 (-2.56-0.09) | 0.064387 | 75-84 | 0.92* (0.46-1.36) | 0.002 |
| 45-54 | 0.23 (-0.81-1.6) | 0.506699 | 85+ | 0.49 (-0.03-1.26) | 0.060788 |
| **Northeast region** | | | | | |
| 15-24 | -1.11* (-2.2--0.22) | 0.019596 | 55-64 | 0.47 (-0.4-1.27) | 0.223555 |
| 25-34 | 0.11 (-1.48-1.36) | 0.910618 | 65-74 | 0.26 (-0.24-0.8) | 0.253149 |
| 35-44 | -0.05 (-2-1.27) | 0.838632 | 75-84 | 0.54* (0.18-1.05) | 0.031994 |
| 45-54 | -0.87* (-1.67--0.21) | 0.015197 | 85+ | 0.65* (0.18-0.94) | 0.005599 |
| **Midwest region** | | | | | |
| 15-24 | - | - | 55-64 | 1.58* (0.89-2.16) | < 0.000001 |
| 25-34 | - | - | 65-74 | 1.34* (1-1.7) | < 0.000001 |
| 35-44 | 1.48* (0.35-2.77) | 0.013197 | 75-84 | 0.93* (0.77-1.14) | < 0.000001 |
| 45-54 | 1.78* (1-2.76) | < 0.000001 | 85+ | 0.92* (0.53-1.14) | < 0.000001 |
| **South region** | | | | | |
| 15-24 | 1.72* (0.71-2.99) | 0.0016 | 55-64 | 1.39* (0.99-1.9) | < 0.000001 |
| 25-34 | 0.46 (-0.62-1.64) | 0.359528 | 65-74 | 1.30* (1.07-1.6) | < 0.000001 |
| 35-44 | 1.45* (0.63-2.46) | 0.0016 | 75-84 | 1.40* (1-1.66) | < 0.000001 |
| 45-54 | 1.84* (1.15-2.75) | < 0.000001 | 85+ | 0.99* (0.69-1.22) | < 0.000001 |
| **West region** | | | | | |
| 15-24 | -0.61 (-2.07-0.57) | 0.223155 | 55-64 | 1.38* (1.02-1.86) | < 0.000001 |
| 25-34 | 1.37* (0.05-3.08) | 0.041192 | 65-74 | 1.23* (0.86-1.69) | < 0.000001 |
| 35-44 | 0.54 (-0.57-1.71) | 0.294341 | 75-84 | 1.26* (1.03-1.55) | < 0.000001 |
| 45-54 | 0.94* (0.16-1.83) | 0.019196 | 85+ | 0.70* (0.31-1.23) | 0.0024 |
| **Metropolitan area** | | | | | |
| 15-24 | 0.75 (-0.46-1.91) | 0.175165 | 55-64 | 1.21* (0.9-1.45) | < 0.000001 |
| 25-34 | 0.38 (-0.58-1.42) | 0.429114 | 65-74 | 1.24* (0.87-1.47) | < 0.000001 |
| 35-44 | 0.41 (-0.34-1.14) | 0.260748 | 75-84 | 1.17* (1-1.31) | < 0.000001 |
| 45-54 | 0.95* (0.55-1.42) | < 0.000001 | 85+ | 0.84* (0.59-1.02) | < 0.000001 |
| **Nonmetropolitan areas** | | | | | |
| 15-24 | - | - | 55-64 | 2.02* (1.35-2.49) | < 0.000001 |
| 25-34 | 1.57* (0.25-3.35) | 0.024395 | 65-74 | 1.28* (0.91-1.75) | < 0.000001 |
| 35-44 | 1.55* (0.51-2.83) | 0.005599 | 75-84 | 1.12* (0.72-1.39) | < 0.000001 |
| 45-54 | 0.83 (-0.13-1.88) | 0.078784 | 85+ | 1.04* (0.61-1.36) | < 0.000001 |
| AAPC = average annual percent change; N.H. = non-Hispanic; * Indicates that the average annual percentage change (AAPC) is significantly different from zero at α = 0.05. | | | | | |

| **Supplementary Table 12:** Death counts and Proportional Mortality Ratio (95% CI) for cardiovascular co-listing on colon cancer death certificates, stratified by age group and demographic subgroup in the United States, 1999–2020 | | | | | | | |
| --- | --- | --- | --- | --- | --- | --- | --- |
| **Proportional Mortality Ratio (95% CI)** | | | | | | | |
| **Cohort** | **Colon Cancer and CVD Deaths (A)** | **Total Colon Cancer Deaths (B)** | **Total CVD Deaths (C)** | **All-Cause Deaths (D)** | **A/B** | **C/D** | **PMR (95% CI)** |
| **15-24 Age Group** | | | | | | | |
| Overall | 98 | 809 | 71932 | 696479 | 0.121 | 0.103 | 1.173 (0.951-1.447) |
| Female | 37 | 328 | 25714 | 182871 | 0.113 | 0.141 | 0.802 (0.571-1.127) |
| Male | 61 | 481 | 46218 | 513608 | 0.127 | 0.090 | 1.409 (1.08-1.84) |
| NH American Indian | 44 | 321 | 891 | 11460 | 0.137 | 0.078 | 1.763 (1.277-2.434) |
| NH Asian Pacific | 7 | 52 | 2440 | 18442 | 0.135 | 0.132 | 1.017 (0.462-2.242) |
| NH Black | 20 | 174 | 18505 | 156196 | 0.115 | 0.118 | 0.97 (0.611-1.542) |
| NH White | 34 | 433 | 38523 | 389760 | 0.079 | 0.099 | 0.794 (0.56-1.127) |
| Hispanic | 35 | 138 | 11448 | 118666 | 0.254 | 0.096 | 2.629 (1.813-3.812) |
| Northeast | 29 | 148 | 11330 | 99222 | 0.196 | 0.114 | 1.716 (1.152-2.556) |
| Midwest | 8 | 165 | 14325 | 151729 | 0.048 | 0.094 | 0.514 (0.253-1.044) |
| South | 31 | 320 | 30401 | 292574 | 0.097 | 0.104 | 0.932 (0.645-1.348) |
| West | 30 | 176 | 15876 | 152954 | 0.170 | 0.104 | 1.642 (1.115-2.419) |
| Metro | 89 | 665 | 58871 | 571297 | 0.134 | 0.103 | 1.299 (1.041-1.621) |
| Non metro | 9 | 144 | 13061 | 125182 | 0.063 | 0.104 | 0.599 (0.305-1.175) |
| **25-34 Age Group** | | | | | | | |
| Overall | 658 | 5334 | 197669 | 1043043 | 0.123 | 0.190 | 0.651 (0.6-0.706) |
| Female | 295 | 2451 | 73210 | 317873 | 0.120 | 0.230 | 0.523 (0.463-0.59) |
| Male | 363 | 2883 | 124459 | 725170 | 0.126 | 0.172 | 0.734 (0.658-0.818) |
| NH American Indian | 113 | 704 | 3134 | 18095 | 0.161 | 0.173 | 0.927 (0.757-1.134) |
| NH Asian Pacific | 47 | 299 | 5837 | 25727 | 0.157 | 0.227 | 0.693 (0.509-0.944) |
| NH Black | 146 | 991 | 54190 | 228840 | 0.147 | 0.237 | 0.622 (0.523-0.74) |
| NH White | 317 | 3154 | 106559 | 613934 | 0.101 | 0.174 | 0.579 (0.516-0.65) |
| Hispanic | 141 | 825 | 27267 | 152348 | 0.171 | 0.179 | 0.955 (0.798-1.142) |
| Northeast | 160 | 853 | 30308 | 162275 | 0.188 | 0.187 | 1.004 (0.848-1.19) |
| Midwest | 73 | 1057 | 39020 | 218478 | 0.069 | 0.179 | 0.387 (0.305-0.49) |
| South | 225 | 2206 | 86053 | 442618 | 0.102 | 0.194 | 0.525 (0.457-0.602) |
| West | 200 | 1218 | 42288 | 219672 | 0.164 | 0.193 | 0.853 (0.734-0.991) |
| Metro | 558 | 4460 | 163401 | 870520 | 0.125 | 0.188 | 0.667 (0.61-0.728) |
| Non metro | 100 | 874 | 34268 | 172523 | 0.114 | 0.199 | 0.576 (0.468-0.709) |
| **35-44 Age Group** | | | | | | | |
| Overall | 3135 | 22965 | 591314 | 1783349 | 0.137 | 0.332 | 0.412 (0.397-0.427) |
| Female | 1391 | 10677 | 216042 | 657934 | 0.130 | 0.328 | 0.397 (0.375-0.42) |
| Male | 1744 | 12288 | 375272 | 1125415 | 0.142 | 0.333 | 0.426 (0.405-0.448) |
| NH American Indian | 22 | 185 | 8131 | 27471 | 0.119 | 0.296 | 0.402(0.258-0.626) |
| NH Asian Pacific | 167 | 975 | 16065 | 42385 | 0.171 | 0.379 | 0.452 (0.383-0.533) |
| NH Black | 757 | 4528 | 153115 | 370955 | 0.167 | 0.413 | 0.405 (0.375-0.438) |
| NH White | 1669 | 14526 | 345816 | 1128801 | 0.115 | 0.306 | 0.375 (0.356-0.395) |
| Hispanic | 512 | 2707 | 66081 | 207045 | 0.189 | 0.319 | 0.593 (0.539-0.652) |
| Northeast | 736 | 3860 | 94303 | 285852 | 0.191 | 0.330 | 0.578 (0.534-0.626) |
| Midwest | 369 | 4747 | 118991 | 372149 | 0.078 | 0.320 | 0.243 (0.219-0.27) |
| South | 1183 | 9691 | 258328 | 760726 | 0.122 | 0.340 | 0.359 (0.338-0.382) |
| West | 847 | 4667 | 119692 | 364622 | 0.181 | 0.328 | 0.553 (0.514-0.595) |
| Metro | 2650 | 19021 | 485353 | 1475982 | 0.139 | 0.329 | 0.424 (0.407-0.441) |
| Non metro | 485 | 3944 | 105961 | 307367 | 0.123 | 0.345 | 0.357 (0.325-0.392) |
| **45-54 Age Group** | | | | | | | |
| Overall | 13217 | 80935 | 1688707 | 3869682 | 0.163 | 0.436 | 0.374 (0.367-0.381) |
| Female | 5590 | 35842 | 592787 | 1483779 | 0.156 | 0.400 | 0.39 (0.379-0.402) |
| Male | 7627 | 45093 | 1095920 | 2385903 | 0.169 | 0.459 | 0.368 (0.359-0.377) |
| NH American Indian | 118 | 661 | 17999 | 44101 | 0.179 | 0.408 | 0.437(0.359-0.532) |
| NH Asian Pacific | 553 | 2816 | 39904 | 85699 | 0.196 | 0.466 | 0.422 (0.385-0.462) |
| NH Black | 3210 | 16049 | 388127 | 744429 | 0.200 | 0.521 | 0.384 (0.369-0.399) |
| NH White | 7791 | 54001 | 1085268 | 2640418 | 0.144 | 0.411 | 0.351 (0.343-0.359) |
| Hispanic | 1502 | 7227 | 150497 | 339146 | 0.208 | 0.444 | 0.468 (0.443-0.495) |
| Northeast | 2918 | 13599 | 282998 | 638744 | 0.215 | 0.443 | 0.484 (0.465-0.504) |
| Midwest | 1960 | 17373 | 349657 | 837077 | 0.113 | 0.418 | 0.27 (0.258-0.283) |
| South | 5127 | 34343 | 719654 | 1623761 | 0.149 | 0.443 | 0.337 (0.327-0.347) |
| West | 3212 | 15620 | 336398 | 770100 | 0.206 | 0.437 | 0.471 (0.453-0.489) |
| Metro | 10941 | 66619 | 1379063 | 3175567 | 0.164 | 0.434 | 0.378 (0.371-0.386) |
| Non metro | 2276 | 14316 | 309644 | 694115 | 0.159 | 0.446 | 0.356 (0.341-0.373) |
| **55-64 Age Group** | | | | | | | |
| Overall | 34714 | 167298 | 3413956 | 6885039 | 0.207 | 0.496 | 0.418 (0.414-0.423) |
| Female | 13495 | 69829 | 1239376 | 2718374 | 0.193 | 0.456 | 0.424 (0.416-0.432) |
| Male | 21219 | 97469 | 2174580 | 4166665 | 0.218 | 0.522 | 0.417 (0.411-0.423) |
| NH American Indian | 280 | 1182 | 29288 | 58938 | 0.237 | 0.497 | 0.477(0.418-0.543) |
| NH Asian Pacific | 1159 | 4668 | 76338 | 146387 | 0.248 | 0.521 | 0.476 (0.446-0.508) |
| NH Black | 7483 | 30134 | 666271 | 1157831 | 0.248 | 0.575 | 0.432 (0.421-0.443) |
| NH White | 22670 | 118888 | 2376345 | 5021170 | 0.191 | 0.473 | 0.403 (0.397-0.409) |
| Hispanic | 3027 | 12026 | 250791 | 473412 | 0.252 | 0.530 | 0.475 (0.456-0.495) |
| Northeast | 7355 | 28558 | 586151 | 1148457 | 0.258 | 0.510 | 0.505 (0.492-0.518) |
| Midwest | 5944 | 36802 | 718988 | 1529325 | 0.162 | 0.470 | 0.344 (0.334-0.353) |
| South | 13536 | 70128 | 1430197 | 2867979 | 0.193 | 0.499 | 0.387 (0.38-0.394) |
| West | 7879 | 31810 | 678620 | 1339278 | 0.248 | 0.507 | 0.489 (0.477-0.501) |
| Metro | 27990 | 134615 | 2749327 | 5556283 | 0.208 | 0.495 | 0.42 (0.415-0.426) |
| Non metro | 6724 | 32683 | 664629 | 1328756 | 0.206 | 0.500 | 0.411 (0.401-0.422) |
| **65-74 Age Group** | | | | | | | |
| Overall | 68523 | 250989 | 5436761 | 10034288 | 0.273 | 0.542 | 0.504 (0.5-0.508) |
| Female | 27281 | 107833 | 2244594 | 4348898 | 0.253 | 0.516 | 0.49 (0.484-0.497) |
| Male | 41242 | 143156 | 3192167 | 5685390 | 0.288 | 0.561 | 0.513 (0.507-0.519) |
| NH American Indian | 394 | 1377 | 36239 | 64742 | 0.286 | 0.560 | 0.511(0.457-0.572) |
| NH Asian Pacific | 1793 | 6004 | 123773 | 211916 | 0.299 | 0.584 | 0.511 (0.485-0.539) |
| NH Black | 11089 | 35640 | 804351 | 1309819 | 0.311 | 0.614 | 0.507 (0.496-0.518) |
| NH White | 50399 | 192630 | 4109156 | 7835707 | 0.262 | 0.524 | 0.499 (0.494-0.504) |
| Hispanic | 4655 | 14751 | 344198 | 580953 | 0.316 | 0.592 | 0.533 (0.515-0.551) |
| Northeast | 15195 | 46925 | 991172 | 1754344 | 0.324 | 0.565 | 0.573 (0.563-0.584) |
| Midwest | 13508 | 58433 | 1176110 | 2280440 | 0.231 | 0.516 | 0.448 (0.44-0.457) |
| South | 24953 | 98158 | 2196329 | 4086490 | 0.254 | 0.537 | 0.473 (0.466-0.48) |
| West | 14867 | 47473 | 1073150 | 1913014 | 0.313 | 0.561 | 0.558 (0.548-0.569) |
| Metro | 54509 | 199305 | 4325213 | 7988242 | 0.273 | 0.541 | 0.505 (0.5-0.51) |
| Non metro | 14014 | 51684 | 1111548 | 2046046 | 0.271 | 0.543 | 0.499 (0.49-0.509) |
| **75-84 Age Group** | | | | | | | |
| Overall | 115427 | 329385 | 8811638 | 14723222 | 0.350 | 0.598 | 0.586 (0.582-0.59) |
| Female | 54383 | 162868 | 4374987 | 7382049 | 0.334 | 0.593 | 0.563 (0.558-0.569) |
| Male | 61044 | 166517 | 4436651 | 7341173 | 0.367 | 0.604 | 0.607 (0.601-0.612) |
| NH American Indian | 428 | 1261 | 36771 | 61070 | 0.339 | 0.602 | 0.564(0.505-0.630) |
| NH Asian Pacific | 2665 | 6895 | 191772 | 297725 | 0.387 | 0.644 | 0.6 (0.574-0.628) |
| NH Black | 12692 | 34570 | 884453 | 1367405 | 0.367 | 0.647 | 0.568 (0.556-0.579) |
| NH White | 93604 | 270533 | 7215497 | 12244092 | 0.346 | 0.589 | 0.587 (0.583-0.592) |
| Hispanic | 5795 | 15476 | 462615 | 721210 | 0.374 | 0.641 | 0.584 (0.566-0.602) |
| Northeast | 27777 | 70312 | 1775939 | 2839920 | 0.395 | 0.625 | 0.632 (0.623-0.641) |
| Midwest | 26190 | 81358 | 2007048 | 3472785 | 0.322 | 0.578 | 0.557 (0.549-0.565) |
| South | 37149 | 115630 | 3277507 | 5592404 | 0.321 | 0.586 | 0.548 (0.542-0.555) |
| West | 24311 | 62085 | 1751144 | 2818113 | 0.392 | 0.621 | 0.63 (0.621-0.64) |
| Metro | 92216 | 263466 | 7063713 | 11821779 | 0.350 | 0.598 | 0.586 (0.581-0.59) |
| Non metro | 23211 | 65919 | 1747925 | 2901443 | 0.352 | 0.602 | 0.584 (0.576-0.593) |
| **85+ Age Group** | | | | | | | |
| Overall | 116752 | 271534 | 11162674 | 16979000 | 0.430 | 0.657 | 0.654 (0.65-0.659) |
| Female | 70945 | 168043 | 7182135 | 10883756 | 0.422 | 0.660 | 0.64 (0.634-0.645) |
| Male | 45807 | 103491 | 3980539 | 6095244 | 0.443 | 0.653 | 0.678 (0.67-0.685) |
| NH American Indian | 268 | 658 | 26972 | 42672 | 0.407 | 0.632 | 0.644(0.559-0.743) |
| NH Asian Pacific | 2454 | 5285 | 226433 | 328516 | 0.464 | 0.689 | 0.674 (0.642-0.707) |
| NH Black | 8742 | 21263 | 785500 | 1169730 | 0.411 | 0.672 | 0.612 (0.597-0.628) |
| NH White | 100667 | 233597 | 9633475 | 14718613 | 0.431 | 0.655 | 0.658 (0.654-0.663) |
| Hispanic | 4404 | 10265 | 470114 | 689855 | 0.429 | 0.681 | 0.63 (0.608-0.652) |
| Northeast | 29583 | 63148 | 2484855 | 3627082 | 0.468 | 0.685 | 0.684 (0.674-0.693) |
| Midwest | 29274 | 70749 | 2696400 | 4199415 | 0.414 | 0.642 | 0.644 (0.636-0.653) |
| South | 33013 | 84994 | 3699313 | 5807840 | 0.388 | 0.637 | 0.61 (0.602-0.618) |
| West | 24882 | 52643 | 2282106 | 3344663 | 0.473 | 0.682 | 0.693 (0.682-0.703) |
| Metro | 93816 | 218902 | 9037541 | 13772027 | 0.429 | 0.656 | 0.653 (0.648-0.658) |
| Non metro | 22936 | 52632 | 2125133 | 3206973 | 0.436 | 0.663 | 0.658 (0.647-0.668) |

| **Supplementary Table 13: Average annual percent change (AAPC) in the Proportional Mortality Ratio for cardiovascular co-listing on colon cancer death certificates, by age group and demographic subgroup in the United States, 1999–2020** | | | | | |
| --- | --- | --- | --- | --- | --- |
| **Proportional Mortality Ratio AAPC (1999-2020)** | | | | | |
| **Age Group** | **AAPC (95% CI)** | **p-value** | **Age Group** | **AAPC (95% CI)** | **p-value** |
| **Overall** | | | | | |
| 15-24 |  |  | 55-64 | 0.58* (0.34-0.86) | < 0.000001 |
| 25-34 | 0.81 (-0.31-2.3) | 0.117576 | 65-74 | 0.77* (0.53-0.99) | < 0.000001 |
| 35-44 | 0.81* (0.28-1.43) | 0.004799 | 75-84 | 0.72* (0.57-0.83) | < 0.000001 |
| 45-54 | 0.63* (0.31-0.99) | 0.0012 | 85+ | 0.26* (0.1-0.4) | 0.003199 |
| **CVD Burden in Colon Deaths (A/B)** | | | | | |
| 15-24 | - | - | 55-64 | 0.8528* (0.5593-1.0497) | < 0.000001 |
| 25-34 | 0.5482 (-0.5707-1.9917) | 0.256349 | 65-74 | 0.5032* (0.3566-0.6585) | < 0.000001 |
| 35-44 | 0.9255* (0.3373-1.666) | 0.003599 | 75-84 | 0.1582* (0.0316-0.2401) | 0.027994 |
| 45-54 | 0.8829* (0.5336-1.3021) | < 0.000001 | 85+ | -0.3422* (-0.4687--0.228) | < 0.000001 |
| **CVD Burden All Cause Deaths (C/D)** | | | | | |
| 15-24 | -0.21 (-0.52-0.14) | 0.137173 | 55-64 | -0.01 (-0.03-0.02) | 0.713457 |
| 25-34 | -0.17 (-0.47-0.09) | 0.153169 | 65-74 | -0.18* (-0.21--0.16) | < 0.000001 |
| 35-44 | -0.03 (-0.12-0.09) | 0.492701 | 75-84 | -0.53* (-0.56--0.5) | < 0.000001 |
| 45-54 | 0.16* (0.09-0.25) | < 0.000001 | 85+ | -0.58* (-0.62--0.55) | < 0.000001 |
| **Female** | | | | | |
| 15-24 |  |  | 55-64 | 0.44* (0.13-0.82) | 0.011198 |
| 25-34 |  |  | 65-74 | 0.80* (0.46-1.1) | < 0.000001 |
| 35-44 | 0.25 (-0.49-1.06) | 0.473505 | 75-84 | 0.67* (0.46-0.87) | < 0.000001 |
| 45-54 | 0.41 (-0.05-0.94) | 0.080384 | 85+ | 0.21* (0.01-0.4) | 0.044391 |
| **Male** | | | | | |
| 15-24 |  |  | 55-64 | 0.67* (0.42-0.98) | < 0.000001 |
| 25-34 | 0.79 (-0.53-2.66) | 0.20156 | 65-74 | 0.66* (0.44-0.88) | < 0.000001 |
| 35-44 | 1.14* (0.55-1.89) | 0.0012 | 75-84 | 0.65* (0.49-0.77) | < 0.000001 |
| 45-54 | 0.74* (0.4-1.16) | < 0.000001 | 85+ | 0.32* (0.1-0.54) | 0.006799 |
| **NH Asian or Pacific Islander** | | | | | |
| 15-24 | - | - | 55-64 | -0.24 (-1.09-0.82) | 0.744251 |
| 25-34 | - | - | 65-74 | -0.33 (-1-0.44) | 0.405119 |
| 35-44 | -1.97* (-3.25--0.83) | 0.0016 | 75-84 | 0.52 (-0.05-1.57) | 0.057588 |
| 45-54 | -0.22 (-1.4-1.38) | 0.895021 | 85+ | -0.86* (-1.28--0.37) | 0.0008 |
| **NH Black or African American** | | | | | |
| 15-24 |  |  | 55-64 | 0.22 (-0.19-0.72) | 0.286743 |
| 25-34 |  |  | 65-74 | 0.34 (-0.02-0.68) | 0.055989 |
| 35-44 | 0.4 (-0.67-1.55) | 0.429914 | 75-84 | 0.13 (-0.09-0.37) | 0.24795 |
| 45-54 | 0.34 (-0.25-0.99) | 0.240352 | 85+ | 0.37 (-0.43-0.86) | 0.159968 |
| **NH White** | | | | | |
| 15-24 |  |  | 55-64 | 0.57* (0.35-0.81) | < 0.000001 |
| 25-34 |  |  | 65-74 | 0.80* (0.57-0.99) | < 0.000001 |
| 35-44 | 1.19* (0.11-2.59) | 0.033593 | 75-84 | 0.65* (0.52-0.78) | < 0.000001 |
| 45-54 | 0.72* (0.28-1.24) | 0.003999 | 85+ | 0.29* (0.17-0.41) | < 0.000001 |
| **Hispanic or Latino** | | | | | |
| 15-24 |  |  | 55-64 | 0.59 (-0.05-1.56) | 0.066787 |
| 25-34 |  |  | 65-74 | 0.50* (0.1-1.02) | 0.018396 |
| 35-44 | -0.32 (-1.28-0.91) | 0.681064 | 75-84 | 0.56* (0.07-0.85) | 0.015197 |
| 45-54 | -0.21 (-1.19-1.06) | 0.894221 | 85+ | 0.07 (-0.43-0.44) | 0.635473 |
| **Northeast region** | | | | | |
| 15-24 |  |  | 55-64 | -0.21 (-0.58-0.15) | 0.24835 |
| 25-34 |  |  | 65-74 | 0.29 (-0.18-0.64) | 0.125975 |
| 35-44 | -0.09 (-1.19-0.95) | 0.773445 | 75-84 | 0.05 (-0.24-0.3) | 0.745451 |
| 45-54 | -0.60* (-1.06--0.18) | 0.009198 | 85+ | 0.19 (-0.26-0.46) | 0.209558 |
| **Midwest region** | | | | | |
| 15-24 | - | - | 55-64 | 0.66* (0.3-1.07) | 0.0012 |
| 25-34 | - | - | 65-74 | 0.63* (0.16-1.15) | 0.010798 |
| 35-44 | 1.37 (-0.13-3.42) | 0.068786 | 75-84 | 0.72* (0.48-0.95) | < 0.000001 |
| 45-54 | 1.08* (0.4-1.93) | 0.004399 | 85+ | 0.44* (0.16-0.7) | 0.0008 |
| **South region** | | | | | |
| 15-24 |  |  | 55-64 | 0.95* (0.55-1.47) | < 0.000001 |
| 25-34 |  |  | 65-74 | 0.94* (0.47-1.28) | < 0.000001 |
| 35-44 | 0.62 (-0.52-2.09) | 0.227155 | 75-84 | 0.94* (0.72-1.11) | < 0.000001 |
| 45-54 | 1.12* (0.62-1.74) | 0.0004 | 85+ | 0.4 (-0.07-0.8) | 0.073985 |
| **West region** | | | | | |
| 15-24 |  |  | 55-64 | 0.82* (0.47-1.25) | < 0.000001 |
| 25-34 |  |  | 65-74 | 0.65* (0.36-1) | 0.0004 |
| 35-44 | 1.48* (0.88-2.36) | < 0.000001 | 75-84 | 0.86* (0.61-1.07) | < 0.000001 |
| 45-54 | 0.72* (0.1-1.46) | 0.020796 | 85+ | 0.13 (-0.08-0.36) | 0.223955 |
| **Metropolitan area** | | | | | |
| 15-24 |  |  | 55-64 | 0.49* (0.25-0.79) | 0.0016 |
| 25-34 | 0.82 (-0.16-2.18) | 0.088382 | 65-74 | 0.69* (0.43-0.94) | < 0.000001 |
| 35-44 | 0.98* (0.27-1.74) | 0.007598 | 75-84 | 0.62* (0.4-0.76) | < 0.000001 |
| 45-54 | 0.50* (0.19-0.84) | 0.004399 | 85+ | 0.23* (0.02-0.39) | 0.031994 |
| **Nonmetropolitan areas** | | | | | |
| 15-24 | - | - | 55-64 | 0.91* (0.39-1.54) | 0.002799 |
| 25-34 |  |  | 65-74 | 0.83* (0.36-1.11) | < 0.000001 |
| 35-44 | 0.34 (-1.24-2.16) | 0.647471 | 75-84 | 0.98* (0.7-1.23) | < 0.000001 |
| 45-54 | 1.19* (0.31-2.32) | 0.011998 | 85+ | 0.59* (0.39-0.83) | < 0.000001 |
| AAPC = average annual percent change; N.H. = non-Hispanic; * Indicates that the average annual percentage change (AAPC) is significantly different from zero at α = 0.05. | | | | | |
